# Supplementary material for: Systematic comparison of Mendelian randomisation studies and randomised controlled trials using electronic databases
Source: BMJ Open. 2023 Sep 26;13(9):e072087. doi: 10.1136/bmjopen-2023-072087 (PMC10533809; doi:10.1136/bmjopen-2023-072087)
Supplement: Supplementary data [file bmjopen-2023-072087supp001.pdf]

Supplementary Box

It is anticipated that the longer duration of exposure differences seen in MR studies will generate larger effect sizes than will relatively short-term modification of the exposure in RCTs. For example, the genetic variants related to non-HDL cholesterol (henceforth "cholesterol", the target of cholesterol lowering drugs such as the statins) have been shown to relate to relatively stable differences from early childhood to late adulthood, thus generating a lifetime of differential exposure to circulating cholesterol. The RCTs of cholesterol lowering drugs designed to show effects on coronary heart disease (CHD) events last ~5 years. Atherosclerosis is a disease process that develops from childhood onwards, and the CHD it generates would not be expected to be abolished by a few years of cholesterol lowering in middle age or older (the usual age included in the RCTs). As there are several cholesterol lowering drugs which target different genes it is possible to compare MR studies using genetic variants in those genes that are robustly related to cholesterol with RCTs of drugs that target those genes. Figure SB1 below combines data from such MR analyses with the RCTs.

As anticipated the RCTs produce about 40% of the risk reduction seen with a lifetime difference in exposure levels[1]. This scaling of the effects predicted from MR studies and seen in the matching trials can be applied to MR/RCT comparisons for other exposures, as the time course of effects being produced may be quicker than seen in the case of cholesterol, or take longer, or indeed there may be no effect in the RCTs if the effect of the exposure acts during a critical period in earlier life and sets in train a disease process that is not reversible by later modification of the exposure[2].

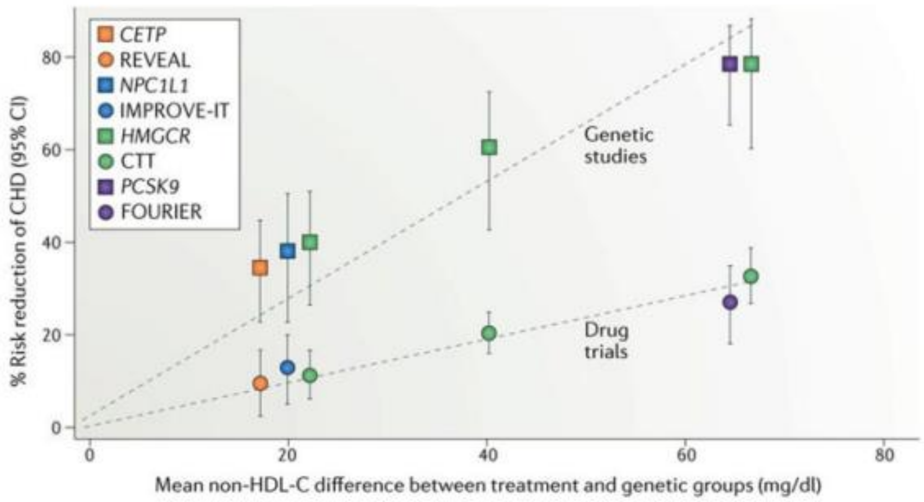

Figure SB1. Drug treatment (circles) and genetic proxy (squares) effects on reducing cholesterol levels and the corresponding reduction in risk of from matching drug RCTs and MR analyses. The colours indicate the gene from which variants are taken in the MR studies and the target of the drug used in the named RCTs. *Abbreviations: CETP, cholesteryl ester transfer protein; HMGCR, 3-hydroxy-3-methylglutaryl-CoA reductase; NPC1L1, Niemann-Pick C1-like protein 1; PCSK9, proprotein convertase subtilisin/kexin type 9.* Figure reproduced with permission from[3].

References

1. Smith GD, Ebrahim S. Mendelian randomization: prospects, potentials, and limitations. Int J Epidemiol. 2004;33: 30–42. doi:10.1093/ije/dyh132

2. Holmes M V, Ala-Korpela M, Smith GD. Mendelian randomization in cardiometabolic disease: challenges in evaluating causality. Nat Rev Cardiol. 2017;14: 577–590. doi:10.1038/nrcardio.2017.78

3. Holmes M V, Smith GD. Revealing the effect of CETP inhibition in cardiovascular disease. Nat Rev Cardiol. 2017;14: 635–636. doi:10.1038/nrcardio.2017.156

Supplementary Note

ClinicalTrials.Gov *Data filtering*

We only included studies with most common designs suitable for comparison with published MR studies: *Parallel Assignment* and *Crossover Assignment* (eliminating: *Single Group Assignment, Sequential Assignment, Factorial Assignment*) intervention model in the Designs table with *Randomized* allocation to allow for selection of RCT. We further used the study type field (=*Interventional*) with a minimum number of arms = *2* in the Studies table as an additional filtering criterion to arrive at a set of RCT studies. The next stage of filtering concerned background information and study results. We first filtered on the presence of a study description in the Brief Summaries table. The key criterion was then presence of results in the Outcome Analyses table, where we selected the variables: *param_type, param_value, p_value* and *method.* Next, we needed all conditions to have at least 1 Medical Subject Headings (MeSH) term assigned in all_conditions view to facilitate automatic comparison with external data sources. We dropped that requirement for interventions, as especially behavioural interventions could not be assigned a MeSH term (see Results). Finally, a range of basic reference and eligibility criteria were required: *brief_title, study_type, overall_status, phase, number_of_arms, enrolment* in the Studies table, *gender, criteria* in the Eligibilities table and *outcome title* and *type* in the Outcomes table.

Additionally, we extracted a subset of studies which did not supply any results in the Outcome Analyses table and therefore did not contribute to the *Main* dataset above but were RCT studies with published literature records in the Study References table (*reference type* = result).

Overview of top SemMed triples for MR and RCT studies

An overview of the top subjects in MR (Figure S4a) and RCTs (Figure S4b) revealed a high number of terms related to adiposity (*obesity, body mass index, adiponectin*), lipid biology (*lipoproteins, hydroxymethylglutaryl-CoA reductase inhibitors, PCKSK9, high density lipoproteins*) as well as *type 2 diabetes* and *vitamin D,* and recently *COVID-19*. Terms related to type 2 diabetes (*insulin, metformin, diabetes*) and *vitamin D* were also found in RCT triples. As expected by the preponderance of drug interventions in ClinicalTrials.Gov, there was a noticeable bias towards pharmaceutical preparations amongst the top 10 subjects in RCT triples with terms such as *methotrexate*, *aspirin*, *clomiphene citrate*, *dexamethasone*, *prednisolone* and *cyclosporine.* SemMedDB identified *associated with* as a top predicate in MR studies (Figure S4c), followed by *predisposes*, *coexists with* and *affects* which underlines the skew of current MR studies towards identifying risk factors for disease. On the other hand, RCT studies lean towards identifying treatments (Figure S4d) which is reflected in the top 1 predicate *treats* (*n*=7,791, second-best *coexists with* n=2,149). Among the top objects in MR studies (Figure S4e), we found cardiovascular diseases (*coronary arteriosclerosis*, *coronary heart disease*, *myocardial ischemia/infarction*, *hypertensive disease*, *ischemic stroke*). High frequency of a smaller number of cardiovascular disease terms (Figure S4f) was also found among RCT objects (*hypertensive disease, cardiovascular disease*). Both MR and

RCT showed type 2 diabetes, obesity, Alzheimer's disease and COVID-19 as commonly studied conditions. RCT objects included also two infectious diseases: malaria and hepatitis C.

Supplementary Figures

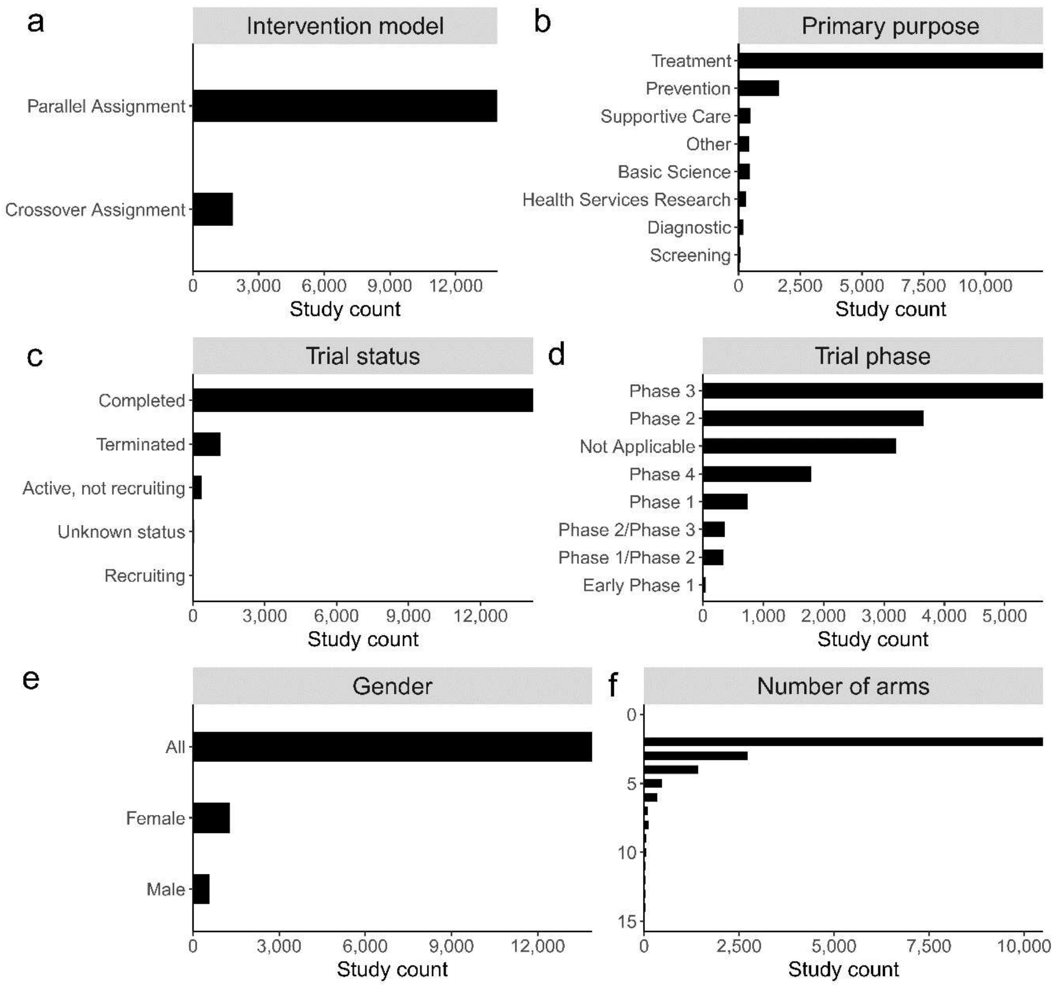

Figure S1. Overview of RCT general features in the Main dataset by their frequency: a) intervention model, b) primary purpose, c) trial status, d) trial phase, e) gender, f) number of arms.

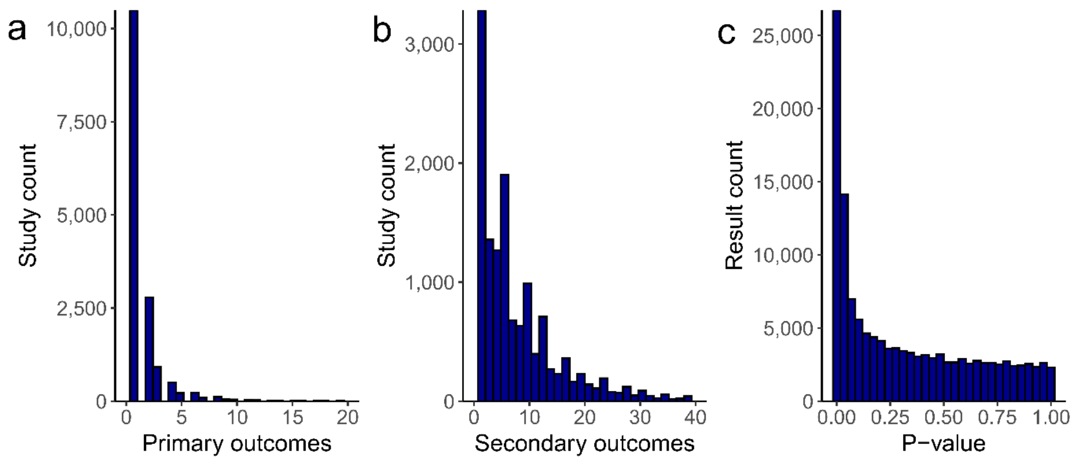

Figure S2. Distribution of: a) primary outcome number among RCT studies, b) secondary outcome number among RCT studies, c) P-value among study results.

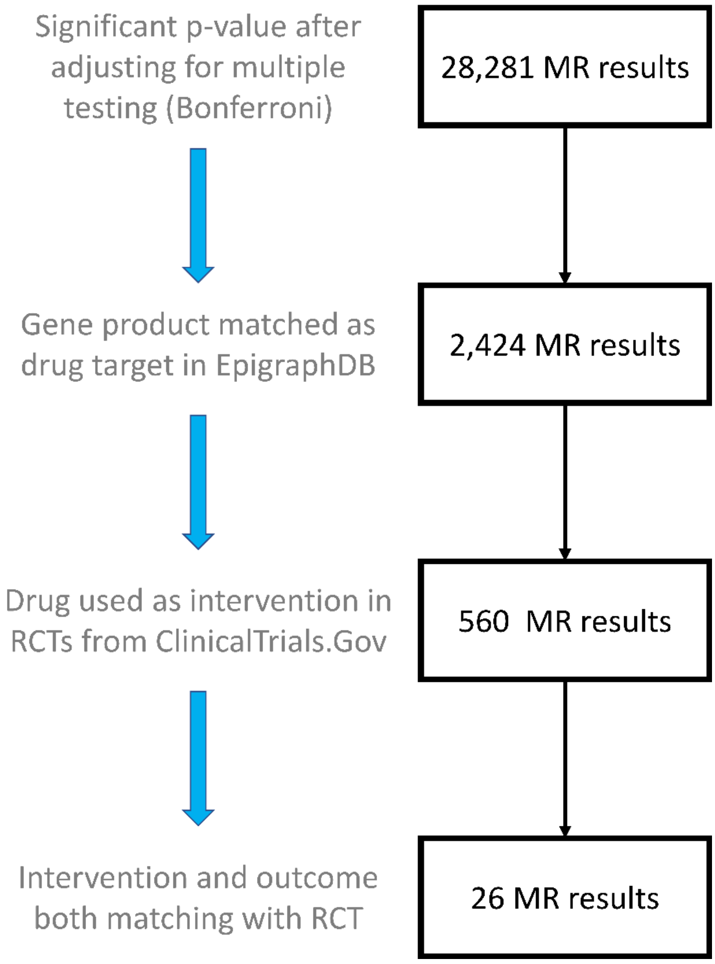

Figure S3. Filtering steps applied to EpigraphDB database. Filtering was designed to identify expression QTL MR studies with intervention and exposure matching those of RCT published on ClinicalTrials.Gov.

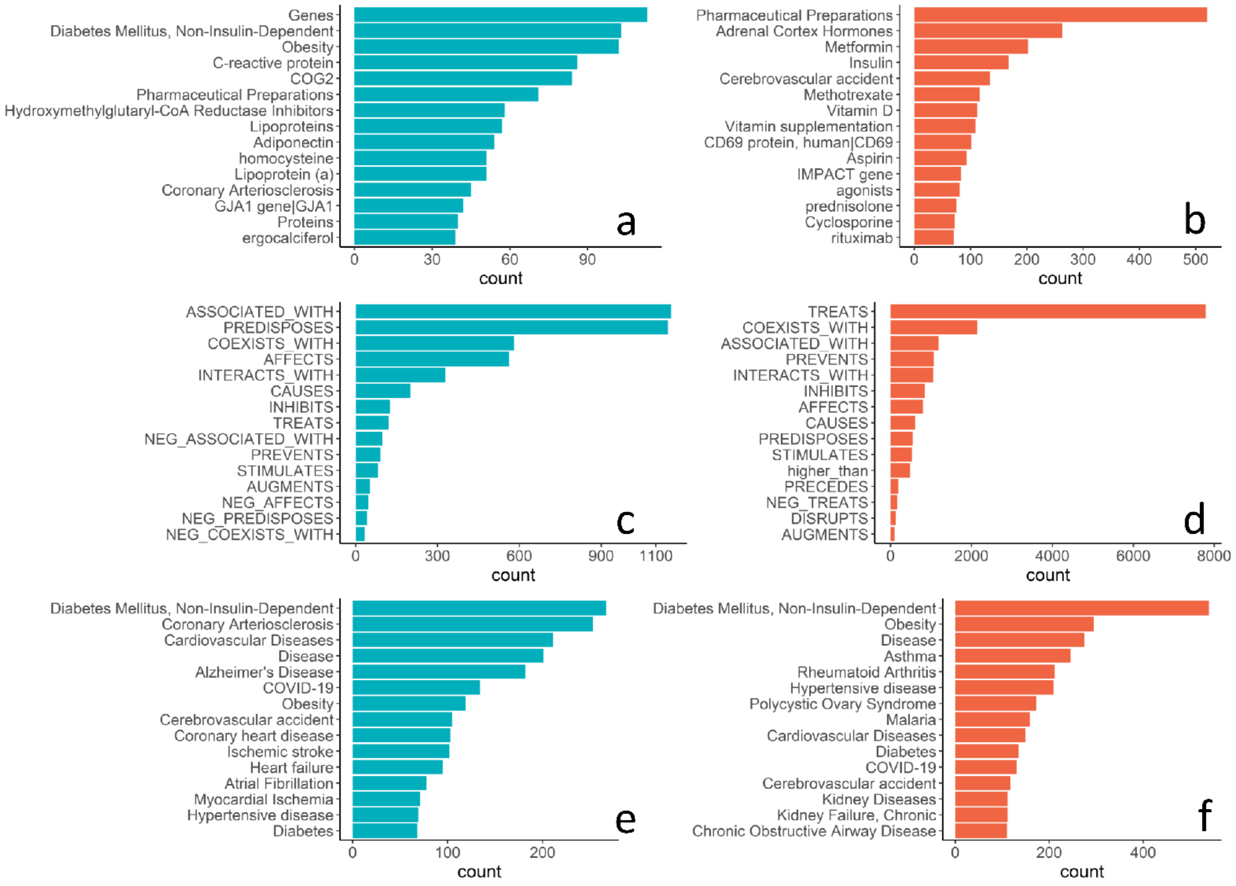

Figure S4. Overview of SemMedDb semantic triples derived from MR (blue, left-hand panels) and RCT (orange, right-hand panels) studies: a) & b) top 15 subjects, c) & d) top 15 predicates, d) & e) top 15 objects.

Supplementary Tables legends

Table S1. Comparison of the most common intervention types in the *Main* and *Literature* datasets.

Table S2. Drug target-disease matches supported by evidence from MR (blood eQTL instruments, https://epigraphdb.org/xqtl) and RCT studies (*Main dataset* from ClinicalTrials.Gov).

Table S3. Matching subject-object pairs between MR and RCT studies in semantic triples identified by SemMedDb.

Table S4. Matching subjects between MR and RCT studies in semantic triples identified by SemMedDb.

Table S5. Matching objects between MR and RCT studies in semantic triples identified by SemMedDb.

Table S6. Sample comparison of MR and RCT studies with matching exposures and outcomes across a range of criteria.

Supplementary Dataset 1. Select ClinicalTrials.Gov data fields for the RCT studies identified as the *main dataset*.

Supplementary Dataset 2. Select ClinicalTrials.Gov data fields for the RCT studies identified as the *literature dataset*.

Supplementary Dataset 3. Comparison of frequency of RCT general features between the *Main dataset* and background of all RCTs available in ClinicalTrials.Gov.

Supplementary Dataset 4. PubMed and SemMedDB data for published MR and RCT studies.

Supplementary Dataset 5. Case series of MR and RCT studies with matching exposures (interventions) and outcomes (conditions).

Table S1

Intervention	Main	Main %	Literature	Literature %
Drug	11,537	73.5%	10,927	38.3%
Other	2,054	13.1%	5,005	17.5%
Biological	1,497	9.5%	1,665	5.8%
Behavioral	1,239	7.9%	3,017	10.6%
Device	1,427	9.1%	3,242	11.4%
Procedure	481	3.1%	3,597	12.6%
Dietary Supplement	242	1.5%	830	2.9%
Radiation	134	0.9%	764	2.7%
Genetic	21	0.1%	145	0.5%
Combination Product	42	0.3%	135	0.5%
Diagnostic Test	15	0.1%	842	3.0%

Table S2

MR Exposure	RCT Drug Intervention	MR Outcomes	RCT Conditions	Matching trials	Concordant direction of effect?	xQTL
CETP	EVACETRAPIB, ANACETRAPIB	LDL cholesterol || id:300, HDL cholesterol || id:299, Total cholesterol || id:301, Triglycerides || id:302	Hyperlipidemia, Dyslipidemia, Hypercholesterolemia, Hyperlipoproteinemia Type II	7	Yes	eQTL
HMGCR	ATORVASTATIN, PRAVASTATIN, SIMVASTATIN	LDL cholesterol || id:300, Total cholesterol || id:301, Non-cancer illness code, self-reported: high cholesterol || id:UKB-b:10912	Hyperlipidemia, Dyslipidemia, Hypercholesterolemia, Mixed Dyslipidemia etc.	50	Yes	eQTL
GUCY1A3 and GUCY1B3	RIOCIGUAT	Diastolic blood pressure automated reading || id:UKB-a:359, Vascular/heart problems diagnosed by doctor: High blood pressure || id:UKB-b:14177	Pulmonary Hypertension, Pulmonary Arterial Hypertension	7	Yes	eQTL
IGF1R	MECASERMIN	Height || id:89	Growth Disorders|Insulin-Like Growth Factor-1 Deficiency	1	No	eQTL
IL2RA	DACLIZUMAB	Type 1 diabetes || id:285	Diabetes Mellitus, Type 1	1	No - no significant effect seen in RCT	eQTL
KCNJ11	GLIMEPIRIDE	Diabetes diagnosed by doctor || id:UKB-b:10753	Diabetes Mellitus, Type 2, Diabetes, Pre-diabetes	63	Yes	eQTL

ACE	ENALAPRIL, LISINOPRIL, PERINDOPRIL	Vascular/heart problems diagnosed by doctor: High blood pressure || id:UKB-b:14177	Hypertension, Essential Hypertension, Hypertension, Pulmonary|Ventricular Dysfunction, Left	3	Yes	eQTL
PDE3A	ANAGRELIDE	Platelet count || id:1008	Thrombocythemia	1	Yes	eQTL
CD86	ABATACEPT	Multiple sclerosis || id:1025	Multiple Sclerosis, Relapsing-Remitting	1	No - no significant effect seen in RCT	eQTL
ESR1	FULVESTRANT, TAMOXIFEN	Breast cancer (Combined Oncoarray; iCOGS; GWAS meta analysis) || id:1126	Breast Cancer, Estrogen Receptor-positive (ER+) Breast Cancer etc.	9	Yes*	eQTL
ESR1	ESTRADIOL, POLYESTRADIOL PHOSPHATE	Femoral neck bone mineral density || id:980, Lumbar spine bone mineral density || id:982, Heel bone mineral density (BMD) T-score automated || id:UKB-a:500	Bone Mineral Density	1	No*	eQTL
PARP3	OLAPARIB, VELIPARIB, RUCAPARIB	Ovarian cancer || id:1120	Ovarian Neoplasms, Ovarian Cancer etc.	7	No	eQTL

MS4A1	OCRELIZUMAB, OFATUMUMAB, RITUXIMAB	Rheumatoid arthritis || id:833, Type 1 Diabetes Mellitus	Rheumatoid Arthritis, Type 1 Diabetes Mellitus	10	No	eQTL
JAK2	TOFACITINIB	Crohn's disease || id:12, Inflammatory bowel disease || id:294	Ulcerative Colitis, Crohn's Disease	6	No	eQTL
TYK2	TOFACITINIB, PEFICITINIB, UPADACITINIB	Rheumatoid arthritis || id:833, Psoriasis || id:282	Rheumatoid arthritis, Psoriasis	34	No	eQTL

Table S3

subject	object	MR	RCT
Obesity	Disease	9	5
Lipoproteins	Cardiovascular Diseases	8	1
Diabetes Mellitus, Non-Insulin-Dependent	Disease	7	2
Obesity	Diabetes Mellitus, Non-Insulin-Dependent	7	6
Asthma	Obesity	6	1
Insulin	Obesity	6	8
Adiponectin	Diabetes Mellitus, Non-Insulin-Dependent	5	1
Coronary Arteriosclerosis	Cardiovascular Diseases	5	1
COVID-19	Disease	5	1
Diabetes Mellitus, Non-Insulin-Dependent	Diabetes	5	5
Disease	Diabetes Mellitus, Non-Insulin-Dependent	5	2
Myocardial Infarction	Cardiovascular Diseases	5	1
Obesity	Asthma	5	4
Pharmaceutical Preparations	Disease	5	14
C-reactive protein	Cardiovascular Diseases	4	1
Cerebrovascular accident	Cardiovascular Diseases	4	1
cytokine	Disease	4	1
ergocalciferol	COVID-19	4	1
Hypertensive disease	Cardiovascular Diseases	4	18
Insulin	Diabetes Mellitus, Non-Insulin-Dependent	4	36
Lipids	Pharmaceutical Preparations	4	1
Lipoproteins	Coronary heart disease	4	1
Pharmaceutical Preparations	Coronary Arteriosclerosis	4	4
Amino Acids, Branched-Chain	Diabetes Mellitus, Non-Insulin-Dependent	3	1
C-reactive protein	Diabetes Mellitus, Non-Insulin-Dependent	3	1
CD69 protein, human|CD69	Cerebrovascular accident	3	1
Diabetes Mellitus, Non-Insulin-Dependent	Cardiovascular Diseases	3	10
Genes	Pharmaceutical Preparations	3	1
High Density Lipoproteins	Coronary Arteriosclerosis	3	1
Hydroxymethylglutaryl-CoA Reductase Inhibitors	Coronary Arteriosclerosis	3	2
Hydroxymethylglutaryl-CoA Reductase Inhibitors	HMGCR gene|HMGCR	3	1
IMPACT gene	Cardiovascular Diseases	3	2
mullerian-inhibiting hormone	Polycystic Ovary Syndrome	3	4
Obesity	Cardiovascular Diseases	3	7
Obesity	COVID-19	3	2
Obesity	Hypertensive disease	3	3
Pharmaceutical Preparations	Cardiovascular Diseases	3	1
Polycystic Ovary Syndrome	Disease	3	1
Vitamin D Deficiency	Cardiovascular Diseases	3	1
Vitamin D	Disease	3	1
Apolipoprotein E|APOE	Alzheimer's Disease	2	2
Atrial Fibrillation	Ischemic stroke	2	1
Cardiovascular Diseases	Diabetes Mellitus, Non-Insulin-Dependent	2	3
Cardiovascular Diseases	Disease	2	1
Coronary Arteriosclerosis	Disease	2	1
Coronary Arteriosclerosis	Heart failure	2	2
COVID-19	Obesity	2	2

Diabetes Mellitus, Non-Insulin-Dependent	COVID-19	2	1
Diabetes Mellitus, Non-Insulin-Dependent	Heart failure	2	1
Diabetic Nephropathy	Diabetes Mellitus, Insulin-Dependent	2	1
Disease	Cerebrovascular accident	2	1
Disease	Multiple Sclerosis	2	1
Disease	Obesity	2	2
Dyslipidemias	Coronary Arteriosclerosis	2	1
ergocalciferol	Cardiovascular Diseases	2	1
ergocalciferol	Disease	2	1
Ethanol	Disease	2	2
GJA1 gene|GJA1	Obesity	2	1
Heart failure	Ischemic stroke	2	1
homocysteine	Alzheimer's Disease	2	2
homocysteine	Cardiovascular Diseases	2	6
Hyperandrogenism	Polycystic Ovary Syndrome	2	5
Hypertensive disease	Cerebrovascular accident	2	4
IMPACT gene	COVID-19	2	2
Myocardial Infarction	Heart failure	2	2
Obesity	Polycystic Ovary Syndrome	2	1
Obesity	Psoriasis	2	1
Pharmaceutical Preparations	Alzheimer's Disease	2	5
Pharmaceutical Preparations	Atherosclerosis	2	1
Pharmaceutical Preparations	Diabetes	2	14
Pharmaceutical Preparations	Parkinson Disease	2	1
Testosterone	Risk factor, cardiovascular	2	1
Vitamin B 12	Body mass index	2	1
Vitamin D	Vitamin D Deficiency	2	3
Vitamin supplementation	Cardiovascular Diseases	2	3
Vitamin supplementation	COVID-19	2	1
Vitamin supplementation	Diabetes Mellitus, Non-Insulin-Dependent	2	3
Vitamin supplementation	Vitamin D Deficiency	2	3
acylcarnitine	Fatty Acids	1	1
Adipokines	Diabetes Mellitus, Non-Insulin-Dependent	1	1
Adipokines	Obesity	1	2
Adiponectin	Obesity	1	1
Age related macular degeneration	Blind Vision	1	2
Alanine Transaminase	Alkaline Phosphatase	1	1
Analgesics	Degenerative polyarthritis	1	4
Angiotensin-Converting Enzyme Inhibitors	Angiotensin Receptor	1	2
Anti-Inflammatory Agents	Disease	1	2
Anticoagulants	Myocardial Ischemia	1	1
Antioxidants	Coronary Arteriosclerosis	1	1
Antioxidants	Rheumatoid Arthritis	1	1
Apolipoproteins B	Apolipoprotein A-I	1	1
Apolipoproteins	Alzheimer's Disease	1	1
ascorbic acid	COVID-19	1	1
Atherosclerosis	Cardiovascular Diseases	1	1
Atherosclerosis	Diabetes Mellitus, Non-Insulin-Dependent	1	1
Atrial Fibrillation	Sleep Apnea, Obstructive	1	1
bempedoic acid	Hypercholesterolemia	1	1

Beta Carotene	Diabetes Mellitus, Non-Insulin-Dependent	1	1
Biological Markers	Alzheimer's Disease	1	1
Body mass index	Diabetes	1	1
C-reactive protein	Disease	1	1
C-reactive protein	Obesity	1	3
Cardiovascular Diseases	Coronary heart disease	1	1
Cardiovascular Diseases	Polycystic Ovary Syndrome	1	1
Carotid Intima-Media Thickness	Arteriosclerosis	1	1
CD69 protein, human|CD69	Alzheimer's Disease	1	1
CD69 protein, human|CD69	Cardiovascular Diseases	1	1
CD69 protein, human|CD69	Hypertensive disease	1	1
CD69 protein, human|CD69	Obesity	1	2
Cerebrovascular accident	Carotid Atherosclerosis	1	1
Cerebrovascular accident	Disease	1	3
Cerebrovascular accident	Ischemic stroke	1	1
Cerebrovascular accident	Myocardial Infarction	1	1
Cigarette smoke (substance)	Tobacco	1	1
coenzyme Q10	Disease	1	1
Complement System Proteins	Disease	1	1
Coronary Arteriosclerosis	Periodontitis	1	1
Coronary heart disease	Obesity	1	1
COVID-19	Critical Illness	1	1
COVID-19	Respiratory Distress Syndrome, Adult	1	2
Crohn's disease	Disease	1	1
cytokine	Rheumatoid Arthritis	1	1
DEFA1A3	Periodontitis	1	1
dexamethasone	COVID-19	1	1
Diabetes Mellitus, Non-Insulin-Dependent	Alzheimer's Disease	1	1
Diabetes Mellitus, Non-Insulin-Dependent	Sleep Apnea, Obstructive	1	2
Diabetes	Obesity	1	4
Diabetes	Polycystic Ovary Syndrome	1	1
Disease	Fatty Liver	1	1
Disease	Liver diseases	1	1
Disease	Lupus Erythematosus, Systemic	1	1
Dyslipidemias	Cardiovascular Diseases	1	2
Enzymes	Vitamin D	1	1
ergocalciferol	calcifediol	1	2
ergocalciferol	Hypertensive disease	1	1
ergocalciferol	Vitamin D Deficiency	1	3
Ethanol	Cardiovascular Diseases	1	1
Folate	homocysteine	1	4
Free thyroxin	Thyrotropin	1	1
Galectin 3	Heart failure	1	1
Gastroesophageal reflux disease	Asthma	1	1
Genes	Dyslipidemias	1	1
Genes	Hypertensive disease	1	1
GJA1 gene|GJA1	Asthma	1	1
GJA1 gene|GJA1	Hypertensive disease	1	1
Glaucoma	Disease	1	1
Glycosylated hemoglobin A	Diabetes Mellitus, Non-Insulin-Dependent	1	2

Hemoglobin	Anemia	1	2
homocysteine	Cerebrovascular accident	1	1
Hydroxymethylglutaryl-CoA Reductase Inhibitors	Atherosclerosis	1	1
Hydroxymethylglutaryl-CoA Reductase Inhibitors	COG2	1	2
Hydroxymethylglutaryl-CoA Reductase Inhibitors	Coronary heart disease	1	1
Hydroxymethylglutaryl-CoA Reductase Inhibitors	Dyslipidemias	1	4
Hyperglycemia	Diabetes Mellitus, Non-Insulin-Dependent	1	1
Hyperlipidemia	Diabetes	1	1
Hyperlipidemia	Disease	1	1
Hypertensive disease	Cardiovascular morbidity	1	3
Hypertensive disease	Myocardial Ischemia	1	1
Hypertensive disease	Obesity	1	4
Hypertensive disease	Sleep Apnea, Obstructive	1	4
Hyperuricemia	Gout	1	1
icosapent ethyl	Hypertriglyceridemia	1	1
IGHE	Disease	1	3
IMPACT gene	Cerebrovascular accident	1	2
IMPACT gene	Disease	1	3
IMPACT gene	Non-alcoholic Fatty Liver Disease	1	1
Inflammatory Bowel Diseases	Crohn's disease	1	1
Insulin-Like Growth Factor Binding Protein 3	Insulin-Like Growth Factor I	1	1
Insulin	Heart failure	1	1
Insulin	Myocardial Infarction	1	1
Insulin	Pancreatitis	1	1
Interleukin-6	Hypertensive disease	1	1
Ischemic stroke	Atrial Fibrillation	1	1
LOC107984137	Periodontitis	1	1
Low-Density Lipoproteins	High Density Lipoproteins	1	1
Malnutrition	Disease	1	1
Metabolic syndrome	Diabetes	1	2
Metabolic syndrome	Obesity	1	3
Metformin	Diabetes	1	6
MTND1P5	Periodontitis	1	1
Myocardial Infarction	Venous Thromboembolism	1	1
Nitrous Oxide	Vitamin B 12	1	1
Non-alcoholic Fatty Liver Disease	Obesity	1	2
Non-alcoholic fatty liver	Diabetes Mellitus, Non-Insulin-Dependent	1	2
Non-alcoholic fatty liver	Fatty Liver	1	1
Obesity	Chronic Disease	1	3
Obesity	Diabetes	1	3
Obesity	Metabolic Diseases	1	1
Periodontitis	Cardiovascular Diseases	1	2
Pharmaceutical Preparations	Asthma	1	18
Pharmaceutical Preparations	Cerebrovascular accident	1	4
Pharmaceutical Preparations	COVID-19	1	2
Pharmaceutical Preparations	Heart failure	1	3
Pharmaceutical Preparations	Hypertensive disease	1	26
Pharmaceutical Preparations	Ischemic stroke	1	1
Pharmaceutical Preparations	Rheumatoid Arthritis	1	5
Proteins	Diabetes	1	1

Rheumatoid Arthritis	Osteoporosis	1	1
Risk factor, cardiovascular	Atherosclerosis	1	1
Risk factor, cardiovascular	Diabetes Mellitus, Non-Insulin-Dependent	1	5
Risk factor, cardiovascular	Hypertensive disease	1	2
Risk factor, cardiovascular	Obesity	1	2
SIGLEC5	Periodontitis	1	1
STN gene|EEF1A2	COG2	1	3
Testosterone	Obesity	1	1
Testosterone	Vitamin D	1	1
tocilizumab	Rheumatoid Arthritis	1	4
Virus Diseases	Common Cold	1	2
Vitamin B 12	homocysteine	1	3
vitamin B12	Vitamin B 12 Deficiency	1	1
Vitamin D	Adiponectin	1	1
Vitamin D	Alzheimer's Disease	1	1
Vitamin D	Asthma	1	1
Vitamin D	Cardiovascular Diseases	1	2
Vitamin D	Diabetes Mellitus, Non-Insulin-Dependent	1	2
Vitamin D	Genes	1	1
Vitamin D	Risk factor, cardiovascular	1	1
Vitamin E	Disease	1	3
Vitamin supplementation	Alzheimer's Disease	1	1
Vitamins	homocysteine	1	1
zinc	COVID-19	1	1

Table S4

subject	MR	RCT
Obesity	36	33
Pharmaceutical Preparations	26	92
Diabetes Mellitus, Non-Insulin-Dependent	20	21
Insulin	13	45
Disease	13	9
Lipoproteins	12	2
Vitamin D	11	13
ergocalciferol	11	8
Hypertensive disease	10	33
Hydroxymethylglutaryl-CoA Reductase Inhibitors	10	10
Coronary Arteriosclerosis	10	5
C-reactive protein	9	6
COVID-19	9	6
Vitamin supplementation	8	11
IMPACT gene	8	10
Cerebrovascular accident	8	7
Myocardial Infarction	7	4
CD69 protein, human|CD69	6	6
Adiponectin	6	2
Asthma	6	1
homocysteine	5	9
Cardiovascular Diseases	5	6
Genes	5	3
cytokine	5	2
GJA1 gene|GJA1	4	3
Testosterone	4	3
Lipids	4	1
mullerian-inhibiting hormone	3	4
Vitamin B 12	3	4
Dyslipidemias	3	3
Ethanol	3	3
Atrial Fibrillation	3	2
Amino Acids, Branched-Chain	3	1
High Density Lipoproteins	3	1
Polycystic Ovary Syndrome	3	1
Vitamin D Deficiency	3	1
Risk factor, cardiovascular	2	10
Diabetes	2	5
Hyperandrogenism	2	5
Metabolic syndrome	2	5
Non-alcoholic fatty liver	2	3
Antioxidants	2	2
Apolipoprotein E|APOE	2	2
Atherosclerosis	2	2
Hyperlipidemia	2	2
Diabetic Nephropathy	2	1
Heart failure	2	1

Metformin	1	6
Analgesics	1	4
Folate	1	4
tocilizumab	1	4
IGHE	1	3
STN gene|EEF1A2	1	3
Vitamin E	1	3
Adipokines	1	2
Age related macular degeneration	1	2
Angiotensin-Converting Enzyme Inhibitors	1	2
Anti-Inflammatory Agents	1	2
Glycosylated hemoglobin A	1	2
Hemoglobin	1	2
Non-alcoholic Fatty Liver Disease	1	2
Periodontitis	1	2
Virus Diseases	1	2
acylcarnitine	1	1
Alanine Transaminase	1	1
Anticoagulants	1	1
Apolipoproteins	1	1
Apolipoproteins B	1	1
ascorbic acid	1	1
bempedoic acid	1	1
Beta Carotene	1	1
Biological Markers	1	1
Body mass index	1	1
Carotid Intima-Media Thickness	1	1
Cigarette smoke (substance)	1	1
coenzyme Q10	1	1
Complement System Proteins	1	1
Coronary heart disease	1	1
Crohn's disease	1	1
DEFA1A3	1	1
dexamethasone	1	1
Enzymes	1	1
Free thyroxin	1	1
Galectin 3	1	1
Gastroesophageal reflux disease	1	1
Glaucoma	1	1
Hyperglycemia	1	1
Hyperuricemia	1	1
icosapent ethyl	1	1
Inflammatory Bowel Diseases	1	1
Insulin-Like Growth Factor Binding Protein 3	1	1
Interleukin-6	1	1
Ischemic stroke	1	1
LOC107984137	1	1
Low-Density Lipoproteins	1	1
Malnutrition	1	1
MTND1P5	1	1

Nitrous Oxide	1	1
Proteins	1	1
Rheumatoid Arthritis	1	1
SIGLEC5	1	1
vitamin B12	1	1
Vitamins	1	1
zinc	1	1

Table S5

object	MR	RCT
Disease	50	51
Cardiovascular Diseases	43	62
Diabetes Mellitus, Non-Insulin-Dependent	37	63
Obesity	29	36
COVID-19	16	12
Alzheimer's Disease	12	14
Coronary Arteriosclerosis	12	9
Cerebrovascular accident	10	13
Diabetes	10	33
Hypertensive disease	10	36
Asthma	8	25
Heart failure	8	9
Polycystic Ovary Syndrome	8	10
Pharmaceutical Preparations	7	2
Coronary heart disease	6	3
Ischemic stroke	6	4
Vitamin D Deficiency	5	9
Atherosclerosis	4	3
Rheumatoid Arthritis	4	11
HMGCR gene|HMGCR	3	1
Risk factor, cardiovascular	3	2
Body mass index	2	1
COG2	2	5
Diabetes Mellitus, Insulin-Dependent	2	1
Dyslipidemias	2	5
homocysteine	2	8
Multiple Sclerosis	2	1
Myocardial Infarction	2	2
Myocardial Ischemia	2	2
Parkinson Disease	2	1
Periodontitis	2	2
Psoriasis	2	1
Sleep Apnea, Obstructive	2	7
Vitamin D	2	2
Adiponectin	1	1
Alkaline Phosphatase	1	1
Anemia	1	2
Angiotensin Receptor	1	2
Apolipoprotein A-I	1	1
Arteriosclerosis	1	1
Atrial Fibrillation	1	1
Blind Vision	1	2
calcifediol	1	2
Cardiovascular morbidity	1	3
Carotid Atherosclerosis	1	1
Chronic Disease	1	3
Common Cold	1	2

Critical Illness	1	1
Crohn's disease	1	1
Degenerative polyarthritis	1	4
Fatty Acids	1	1
Fatty Liver	1	1
Genes	1	1
Gout	1	1
High Density Lipoproteins	1	1
Hypercholesterolemia	1	1
Hypertriglyceridemia	1	1
Insulin-Like Growth Factor I	1	1
Liver diseases	1	1
Lupus Erythematosus, Systemic	1	1
Metabolic Diseases	1	1
Non-alcoholic Fatty Liver Disease	1	1
Osteoporosis	1	1
Pancreatitis	1	1
Respiratory Distress Syndrome, Adult	1	2
Thyrotropin	1	1
Tobacco	1	1
Venous Thromboembolism	1	1
Vitamin B 12	1	1
Vitamin B 12 Deficiency	1	1

Table S6

	MR	RCT	
	**Mokry 2015**	**James 2013**	**Hempel 2017**
**Exposure:**	vitamin D	vitamin D	vitamin D
**Outcome(s):**	multiple sclerosis	multiple sclerosis relapse	multiple sclerosis as measured with EDSS score
**Population (Sex, ethnicity and age):**	mixed, European	mixed, European	mixed, European
**Population (health status):**	all	meta-analysis of 5 RCTs	meta-analysis of 5 RCTs
**Comparator group:**	NA	low dose vitamin D, placebo	low dose vitamin D, placebo
**Prevention or Treatment:**	prevention	treatment	treatment
**Direction of effect:**	negative	null	null
**Intervention duration:**	lifetime	range: 6 months-96 weeks	range: 6 months-24 months
**Statistic:**	odds ratio	odds ratio	standardized mean difference
**Citations no:**	340	122	38
