# Supplementary material for: Systematic comparison of Mendelian randomisation studies and randomised controlled trials using electronic databases
Source: BMJ Open. 2023 Sep 26;13(9):e072087. doi: 10.1136/bmjopen-2023-072087 (PMC10533809; doi:10.1136/bmjopen-2023-072087)
Supplement: Author's manuscript [file bmjopen-2023-072087.draft_revisions.pdf]

BMJ Open is committed to open peer review. As part of this commitment we make the peer review history of every article we publish publicly available.

When an article is published we post the peer reviewers' comments and the authors' responses online. We also post the versions of the paper that were used during peer review. These are the versions that the peer review comments apply to.

The versions of the paper that follow are the versions that were submitted during the peer review process. They are not the versions of record or the final published versions. They should not be cited or distributed as the published version of this manuscript.

BMJ Open is an open access journal and the full, final, typeset and author-corrected version of record of the manuscript is available on our site with no access controls, subscription charges or pay-per-view fees (http://bmjopen.bmj.com).

If you have any questions on BMJ Open's open peer review process please email info.bmjopen@bmj.com

BMJ Open

Systematic comparison of Mendelian randomization studies and randomized controlled trials using electronic databases

Journal:	*BMJ Open*
Manuscript ID	bmjopen-2023-072087
Article Type:	Original research
Date Submitted by the Author:	23-Jan-2023

Complete List of Authors:	Sobczyk, Maria; MRC Integrative Epidemiology Unit,<br>Zheng, Jie; MRC Integrative Epidemiology Unit<br>Davey Smith, George; MRC Integrative Epidemiology Unit<br>Gaunt, Tom; MRC Integrative Epidemiology Unit

Keywords:	EPIDEMIOLOGIC STUDIES, EPIDEMIOLOGY, Clinical trials < THERAPEUTICS

SCHOLARONE™
Manuscripts

I, the Submitting Author has the right to grant and does grant on behalf of all authors of the Work (as defined in the below author licence), an exclusive licence and/or a non-exclusive licence for contributions from authors who are: i) UK Crown employees; ii) where BMJ has agreed a CC-BY licence shall apply, and/or iii) in accordance with the terms applicable for US Federal Government officers or employees acting as part of their official duties; on a worldwide, perpetual, irrevocable, royalty-free basis to BMJ Publishing Group Ltd ("BMJ") its licensees and where the relevant Journal is co-owned by BMJ to the co-owners of the Journal, to publish the Work in this journal and any other BMJ products and to exploit all rights, as set out in our licence.

The Submitting Author accepts and understands that any supply made under these terms is made by BMJ to the Submitting Author unless you are acting as an employee on behalf of your employer or a postgraduate student of an affiliated institution which is paying any applicable article publishing charge ("APC") for Open Access articles. Where the Submitting Author wishes to make the Work available on an Open Access basis (and intends to pay the relevant APC), the terms of reuse of such Open Access shall be governed by a Creative Commons licence – details of these licences and which Creative Commons licence will apply to this Work are set out in our licence referred to above.

Other than as permitted in any relevant BMJ Author's Self Archiving Policies, I confirm this Work has not been accepted for publication elsewhere, is not being considered for publication elsewhere and does not duplicate material already published. I confirm all authors consent to publication of this Work and authorise the granting of this licence.

For peer review only

Systematic comparison of Mendelian randomization studies and randomized controlled trials using electronic databases

Maria K. Sobczyk[1]*, Jie Zheng[1,2,3], George Davey Smith[1], Tom R. Gaunt[1]

*To whom correspondence should be addressed.

1) MRC Integrative Epidemiology Unit, Bristol Medical School, University of Bristol, Bristol BS8 2BN, UK

2) Department of Endocrine and Metabolic Diseases, Shanghai Institute of Endocrine and Metabolic Diseases, Ruijin Hospital, Shanghai Jiao Tong University School of Medicine, Shanghai, China

3) Shanghai National Clinical Research Center for Metabolic Diseases, Key Laboratory for Endocrine and Metabolic Diseases of the National Health Commission of the PR China, Shanghai Key Laboratory for Endocrine Tumor, State Key Laboratory of Medical Genomics, Ruijin Hospital, Shanghai Jiao Tong University School of Medicine, Shanghai, China

Corresponding author: Maria K. Sobczyk (maria.sobczyk@bristol.ac.uk)

Keywords:

Mendelian randomization

Randomized Controlled Trials

Data mining

Literature mining

For peer review only

Abstract

Objective: To scope the potential for (semi-)-automated triangulation of Mendelian Randomization (MR) and randomized controlled trials (RCT) evidence since the two methods have distinct assumptions that make comparisons between their results invaluable.

Methods: We mined ClinicalTrials.Gov, PubMed and EpigraphDB databases and carried out a series of 26 manual literature comparisons among 54 MR and 77 RCT publications.

Results: We found that only 11% of completed RCTs identified in ClinicalTrials.Gov submitted their results to the database. Similarly low coverage was revealed for Semantic Medline (SemMedDB) semantic triples derived from MR and RCT publications –25% and 12%, respectively. Among intervention types that can be mimicked by MR, only trials of pharmaceutical interventions could be automatically matched to MR results due to insufficient annotation with MeSH ontology. A manual survey of the literature highlighted the potential for triangulation across a number of exposure/outcome pairs if these challenges can be addressed.

Conclusions: We conclude that careful triangulation of MR with RCT evidence should involve consideration of similarity of phenotypes across study designs, intervention intensity and duration, study population demography and health status, comparator group, intervention goal and quality of evidence.

For peer review only - http://bmjopen.bmj.com/site/about/guidelines.xhtml

WHAT IS ALREADY KNOWN ON THIS TOPIC

Mendelian randomisation (MR) has become a popular method in causal inference in genetic epidemiology, and while often used as proxy for to randomized clinical trials (RCT), little is known about scope for automatic comparison between MR and RCT results.

Previous research has established conceptual similarities and differences between MR and RCT methodology, however, without focus on applied cases.

WHAT THIS STUDY ADDS

The study found that a low percentage of completed RCTs were submitted to ClinicalTrials.gov and that a similarly low coverage was found for MR and RCT publications in Semantic Medline. Only trials of pharmaceutical interventions could be automatically matched to MR results due to insufficient annotation with MeSH ontology among other interventions.

Following manual extraction of MR and RCT literature, we assessed result concordance across the two methods and discussed multiple possible reasons for discrepancies.

HOW THIS STUDY MIGHT AFFECT RESEARCH, PRACTICE OR POLICY

Sparsity of data in electronic databases hinders the ability to automatically compare results of MR and RCT studies. In the absence of retrospective manual extraction of MR and RCT results from publications, more research effort needs to be spend developing machine-learning approaches to aid systematic comparisons. Our study helps identify study design features which need to be captured by such methods.

For peer review only - http://bmjopen.bmj.com/site/about/guidelines.xhtml

Background

Randomized controlled trials (RCTs) are deemed the “gold standard” in evaluating the efficacy of interventions and guiding practice in clinical research, with well-established methodology[1]. In RCTs, a selection of individuals intended to represent the target population is randomly assigned to a treatment or control group, allowing estimation of the intervention’s effectiveness in the absence of confounding variables and reverse causality that are present in observational studies. In the past two decades an approach to causal inference using natural genetic variation, known as Mendelian Randomization (MR) – usually implemented as an instrumental variable (IV) analyses – has gained popularity[2,3]. This approach has been referred to as “nature’s randomized trials”[4], and is based on the randomization from parents to offspring of genetic variants encapsulated in Mendel’s laws of segregation and independent assortment[2,5]. At a population level the randomization is approximate, but still allows genetic variants that are robustly associated with the measured exposure to be used to estimate the unbiased causal effect of an exposure (generally acting across life) on health outcomes, as long as certain assumptions, discussed in detail elsewhere[2,3,6], are met.

Despite drawing on observational data, the MR approach broadly aligns with that of a randomized controlled trial, where the goal is to estimate the causal effect of an intervention on the given endpoint based on groups (arms) which do not differ with respect to confounding variables (Figure 1). However, since in MR randomization takes place at conception, the time lag to the start of outcome recoding is longer[7] compared to RCTs, where median duration of phase 3 trials is 40 months[8]. Similarly to RCTs, most MR

For peer review only - http://bmjopen.bmj.com/site/about/guidelines.xhtml

analyses should be free of confounding and reverse causation bias due to variants being allocated randomly before birth and outcome condition onset.

Previous research has shown examples of evidence triangulation where MR results predicted the overall RCT results based on totally orthogonal data with an unrelated set of systematic errors and biases[9]. For instance, MR demonstrated the lack of effect of genetically-predicted concentrations of HDL-C on cardiovascular events[10–12] as well as selenium in prostate cancer prevention trials[13,14]. On the other hand, MR showed the beneficial effect of lifelong endogenous low LDL-C levels[15], HMG-CoA reductase inhibition (statin drug target) and PCSK9 inhibition on cardiovascular disease[15,16], while predicting also the increased risk of type 2 diabetes (T2D) as a side-effect of statin usage. However, three independent MR studies were at odds with later RCTs by predicting increased risk of T2D also as a side-effect of PCK9 inhibition[17].

There are several possible explanations for apparent or real discordance in the results of RCT and MR studies. These range from different durations, magnitude and time-varying nature of the exposure, origin of the study populations, and natural genetic variation imperfectly mimicking the molecular action of the drug, some of which we explore. The direct comparison of MR and RCT findings is facilitated by the use of a precisely-defined estimand[18], for example, the effect on incident coronary heart disease risk of lowering LDL cholesterol by 1 mmol/l for 5 years. Whilst RCTs will estimate something close to this, and be scalable to it, with MR studies the exposure difference associated with the genetic instruments will often exist from birth (or before) and may change in magnitude over time[19]. This is discussed further in the Supplementary Box.

For peer review only - http://bmjopen.bmj.com/site/about/guidelines.xhtml

Whilst RCTs can provide the highest-quality evidence, they may have limitations. They are often expensive to carry out, can be of small size and lack external validity[20], have short follow-up and typically take place after disease onset[21,22]. As in other study types, RCT results may be flawed due to poor design and execution, e.g. imperfect randomization, unblinding and differential loss to follow-up between study arms.

Unlike RCTs, MR studies are inexpensive and quick to perform when suitable genetic instruments are available. Therefore, they can potentially prioritize intervention-condition pairs to assess in RCTs. Moreover, it has been proposed that MR also guide the design of RCTs, improving eligibility criteria to prioritise groups most likely to benefit, suggesting diseases for composite endpoint construction, and alerting to potential side-effects[1,23]. Since MR analyses suffer a different set of biases than RCTs, MR evidence can be used to complement RCTs and other study designs in the triangulation framework to guide therapeutic development and clinical practice[24–26]. Finally, the extensive use of existing observational data for MR enables intervention targets to be evaluated in a wider range of sub-populations than is feasible for RCTs (improving generalizability), and allows comparisons to be made that might be unethical in experimental studies, for example when there is strong evidence in favour of a particular treatment.

The goal of this research is to survey the extent of concordance between MR and RCT studies to date and identify possible factors for disparities in the direction of effect, which limit the ability to extrapolate from MR results to RCTs and increase the complexity of the triangulation process. In this study, we aimed to carry out a systematic analysis of MR and RCT results using automated mining of data in the public domain, including the ClinicalTrials.Gov[27], EpigraphDB[28] and PubMed databases. We evaluate the

For peer review only - http://bmjopen.bmj.com/site/about/guidelines.xhtml

comprehensiveness and scope of the data available and potential for comparative analyses between MR and RCTs. We then go on to develop a series of case-studies looking in detail at MR and RCT comparisons across 26 exposure-outcome pairs. Throughout, we use the term “intervention” as synonymous with “exposure” and “condition” as synonymous with “outcome”.

Methods

ClinicalTrials.Gov *Data sources*

All ClinicalTrials.Gov study data is available for download as PostgreSQL database from The Database for Aggregate Analysis of ClinicalTrials.gov (AACT)[29] released by The Clinical Trials Transformation Initiative[30]. We downloaded its static release from 1st August 2021. Processing of the database files was carried out using custom Python and R scripts.

ClinicalTrials.Gov *Data filtering*

We filtered the ClinicalTrials.Gov studies using a number of criteria to identify RCTs with submitted results allowing direct comparison with MR studies. These are depicted in Figure 2 and provided in detail in Supplementary Note.

EpigraphDB *queries*

EpigraphDB[28] was used to collect information about confirmed drug-target associations which were initially sourced from the Open Targets Platform[31] and verified in DrugBank[32]. We also used EpigraphDB[28] to source SemMedDB[33] semantic triples associated with select MR and RCT publications identified by PubMed. SemMedDB triples in EpigraphDB are prefiltered for annotation of epidemiological studies as described previously[34].

For peer review only - http://bmjopen.bmj.com/site/about/guidelines.xhtml

PubMed *Data harvesting*

We searched PubMed for all RCT and MR studies published before 2021 on 1st October 2021. For RCTs, we searched titles and abstracts for keywords: "randomized controlled trial" or "RCT" and we used PubMed's in-built *Randomized Controlled Trial* label filter to obtain more specific hits, reducing the number of hits from 112,015 to 63,187. In order to retrieve potential MR studies, we used the keywords: "mendelian randomization" or "mendelian randomisation". We also considered using MeSH labels "Randomized Controlled Trial" and "Mendelian Randomization Analysis" but both in case of RCT and MR studies they returned unrealistically low number of hits (7,999 and 1,926, accordingly), a consequence of manual indexing.

Literature searches

We used Semantic Scholar and Google Scholar to survey MR and RCT literature indexed before 1st August 2021. We queried the databases with the following search terms: "[exposure] [condition] Mendelian Randomization" and "[exposure] [condition] Randomized Controlled Trial". The articles were initially screened by title and abstract. We included original research MR, RCT studies as well as meta-analyses. As exposures, we chose common lifestyle risk factors for chronic disease[35] – body mass index (BMI), smoking, alcohol intake, blood pressure. Examples of dietary intervention (vitamin D/E, coffee) and behavioural intervention (exercise) were also selected. Outcomes included a number of cardiovascular disease and risk biomarkers, glycemic traits, neuropsychiatric disease, bone mineral density, atopic dermatitis and prostate cancer, amongst others (Supplementary Dataset 4[36]). MR and RCT studies were compared across: population characteristics (sex, ethnicity, age, health status), comparator group, goal of intervention (prevention or

For peer review only

For peer review only - http://bmjopen.bmj.com/site/about/guidelines.xhtml

treatment/slowing progression), direction of effect, length of follow-up, main test statistic in the study and its impact as judged by citation number.

Patient and Public Involvement

Patients or the public were not involved in the design, or conduct, or reporting, or dissemination plans of our research.

Results

ClinicalTrials.Gov data overview

In total, we found 379,094 individual studies were registered with a unique ClinicalTrials.gov identifier. We filtered them using a number of steps to identify RCTs and facilitate comparison with MR (Figure 2). In our analysis, we identified 166,954 RCT studies (44% of the total). To allow semi-automated comparison with MR studies, we focussed on the study subset which submitted their statistical analysis results to the database (referred to as the **main dataset,** Supplementary Dataset 1[36]). However, we found that only 4% of studies – 13,807 met this criterion, along with including background information on the trial. To expand that number, we also considered an additional 23,080 RCT studies which did not publish their results in ClinicalTrials.gov but instead linked to a peer-reviewed publication (referred to as the **literature dataset,** Supplementary Dataset 2[36]).

The majority of RCTs in the main dataset followed parallel assignment of participants to treatment (Figure S1a), most were designed for treatment (n=10,812, Figure S1b), rather than prevention (n=1,422) and the vast majority of them had been completed (Figure S1c). More trials were observed to be in phase 3 than 4 (Figure S1d), most trials included both males and females (Figure S1e), and a great majority had 2 arms (Figure S1f). The median

For peer review only - http://bmjopen.bmj.com/site/about/guidelines.xhtml

For peer review only

number of primary outcomes was 1 (Figure S2a), with a median of 5 secondary outcomes (Figure S2b). Over half of studies report at least 1 result with *p*-value less than 0.05 (Figure S2c). Comparison with features of all RCTs in the database showed that our selection was broadly representative (Supplementary Dataset 3[36]), although our dataset was enriched for completed and late-phase trials.

Suitability of MeSH annotation

In order to attempt automated matching of RCTs and MRs involving similar interventions and outcomes for RCTs and MR, we needed to first establish the quality of annotation of RCTs with MeSH (Medical Subject Headings) in ClinicalTrials.Gov. The most common intervention was Drug (Table S1). Since we were only interested in the intervention types which can be instrumented by MR, we also focussed on the 4th and 7th most popular types of interventions: Behavioral and Dietary Supplement. We found that MeSH intervention annotations were missing for only 19% and 16% of Drug interventions in the Main and Literature datasets, accordingly (Table 1). However, the overwhelming majority of RCTs in the Behavioral and Dietary Supplement category did not contain a MeSH intervention term. Due to well standardised disease taxonomy, a much lower level of missing data was found for MeSH condition terms. This allowed us to proceed with automated analysis of Drug RCT data, however, for Behavioral and Dietary Supplement we were only able to do a manual screening for RCTs with corresponding MR studies.

For peer review only

Table 1. Completeness of MeSH term annotation amongst the chosen intervention types in the *Main* (RCT results available in ClinicalTrials.Gov) and *Literature* (RCT results unavailable in ClinicalTrials.Gov but study linked to a publication with results) datasets.

Dataset	Intervention type	Total	intervention MeSH missing	% missing	condition MeSH missing	% missing
Main	Drug	10,187	1,915	18.8%	936	9.2%
Literature	Drug	9,554	1,526	16.0%	914	9.6%
Main	Behavioral	1,013	872	86.1%	203	20.0%
Literature	Behavioral	2,427	2,248	92.6%	496	20.4%
Main	Dietary Supplement	215	102	47.4%	30	14.0%
Literature	Dietary Supplement	662	449	67.8%	118	17.8%

For peer review only

Pharmaceutical interventions in RCTs and MR

Genetic instrumental variables in MR can be used as proxies for pharmaceutical interventions in RCTs. Protein (pQTL) or expression (eQTL) quantitative trait loci (QTL), i.e. variants associated with expression of protein drug targets are used to directly proxy the action of a drug. Here we use the biggest MR dataset for drug target protein-disease associations, examined in whole blood, from Zheng et al. (2020)[37]. We focussed on *cis*-acting instruments as a more specific marker for drug efficacy as *trans*- instruments are more likely to be pleiotropic, potentially leading to spurious results[23].

We matched the drug target proteins in Zheng et al. (2020) with drug-gene associations sourced from EpigraphDB[28]. This allowed us to merge the Zheng et al. (2020) dataset with the main and literature RCT dataset via the drug listed in EpigraphDB and MeSH drug intervention term, accordingly. For the outcome, we were then able to match RCTs and MR manually due to the reasonably low number of hits. The results, displayed in Table 2 show overlap of the RCT and MR datasets. We found 4 drugs: evolocumab/alirocumab, ustekinumab and mipomersen that share support from both MR and RCT studies. evolocumab/alirocumab and mipomersen inhibit key players (PCSK9 and apoB) in lipid transport helping to lower plasma LDL-C levels[38]. The Zheng et al. (2020) MR study showed a negative effect of reduced PCSK9 levels on high cholesterol in the UK Biobank, while in 25 RCT studies drug-induced abrogation of PCSK9 activity led to positive outcomes in the treatment of hyperlipidemia, hypercholesterolemia and dyslipidemias in general. Similarly, reduced expression/activity of apoB in MR and 6 RCT studies resulted in genetically predicted lower levels of LDL cholesterol and total cholesterol in the UK Biobank as well as improved outcomes in the treatment of dyslipidemias, respectively. The third

For peer review only

Table 2. Drug target-disease matches supported by evidence from MR (blood pQTL instruments, Zheng et al. 2020) and RCT studies (*Main dataset* from ClinicalTrials.Gov).

MR Exposure	RCT Drug Intervention	MR Outcomes	RCT Conditions	Matching trials	Concordant direction of effect?	xQTL
PCSK9	EVOLOCUMAB, ALIROCUMAB	Non-cancer illness code self-reported: high cholesterol || id:UKB-a:108	Hyperlipidemia, Dyslipidemia, Hypercholesterolemia, Mixed Dyslipidemia	25	Yes	pQTL
APOB	MIPOMERSEN	LDL cholesterol || id:300, HDL cholesterol || id:299, Triglycerides || id:302, Non-cancer illness code self-reported: high cholesterol || id:UKB-a:108, Total cholesterol || id:301,	Hyperlipidemia, Dyslipidemia, Hypercholesterolemia, Mixed Dyslipidemia	6	Yes	pQTL
IL12B	USTEKINUMAB	Non-cancer illness code self-reported: psoriasis || id:UKB-a:100; Ulcerative colitis || id:970; Crohn's disease || id:12; Inflammatory bowel disease || id:294	Psoriasis, Psoriatic Arthritis, Crohn's Disease, Colitis, Inflammatory Bowel Disease	21	Yes	pQTL

For peer review only

example of a good match between RCT and MR studies concerns inhibition of the p40 subunit of interleukin 12 and 23 (IL12B)[39]. Both MR and 21 RCTs show benefit of inhibition of p40 on immune-mediated disease: psoriasis and inflammatory bowel disease.

In general, pQTL MR-based prediction of drug target-condition pairs offered good recall when compared with the pairs in the Open Targets Platform for the proteins with MR evidence. The only drug target indications missing included conditions not analysed in the MR study, such as CD33 protein being the drug target for treatment of leukemia, with the exception of ACHE (acetylocholinoesterase) whose inhibitors (galantamine, donepezil, rivastigmine) are used for treatment of cognitive decline in Alzheimer's disease[40].

We also compared the RCT dataset with Zheng et al. blood transcript expression (eQTL)-derived MR analysis (available in EpigraphDB: https://epigraphdb.org/xqtl). In total, we identified 15 drug target-disease matches in the eQTL dataset (Table S2), although unlike in the pQTL matches, the direction of effect in MR was incorrect in 8 cases. Nevertheless, the eQTL MR results agreed with some well-known drug effects: HDL-C and LDL-C lowering action of CETP and HMGCR inhibitors[41], respectively, and blood pressure-lowering action of ACE inhibitors[42].

PubMed-sourced MR and RCT studies

In addition to searching through the ClincalTrials.Gov database, we also queried PubMed for RCT and MR publications. In total, we found 3,538 MR studies published since mid-2000s and 63,187 RCTs published since 1970 until 2020 (Figure 3).

Semantic analysis with SemMedDB

For peer review only

We subsequently wanted to establish the thematic overlap between MR and RCT studies using an alternative method involving semantic analysis. SemMedDB[33] provides a vast repository of semantic predications (subject-predicate-object triple, e.g. LDL-C causes ischemic heart disease). We linked the MR and RCT publications identified by our PubMed search to their corresponding SemMed triples in EpigraphDB using PubMed ID. Overall, only 12% and 25% of RCT and MR papers, respectively, had a semantic triple associated with them (Figure 3). When ignoring the predicate, and focusing only on the subject and object, we found a total of 10,875 unique exposure-outcome pairs, discussed in detail in the Supplementary Note. However, only 125 of these were found to be shared across MR and RCT studies.

We then investigated the 125 matching subject-object pairs between MR and RCT studies (Table S3), as well as individual top counts among subjects (Table S4) and objects (Table S5). T2D, insulin and obesity were found among the top shared risk factors, along with lipids and vitamin D. Top outcomes included T2D, cardiovascular disease, asthma and Alzheimer's disease.

Case studies of matching MR and RCTs

Since our semi-automatic mining of MR and RCT literature brought limited results for behavioural and nutritional interventions, we selected 26 intervention-outcome case-studies by manual mining of the literature representing common lifestyle risk factors, dietary and behavioural exposures, paired with common cardiovascular, glycemic, neuropsychiatric, musculoskeletal, autoimmune and cancer outcome phenotypes. In total, we surveyed 54 MR and 77 randomized controlled trial publications (RCTs and meta-analysis of RCTs, Supplementary Dataset 4[36], Figure 4) which were systematically compared across

For peer review only - http://bmjopen.bmj.com/site/about/guidelines.xhtml

several criteria shown in sample Table S6, and encompass those in the popular PICO (Population, Intervention, Comparison, and Outcome) framework[43].

There, we compare an MR study and two RCT meta-analyses on the effect of vitamin D supplementation in multiple sclerosis (MS)[44–46]. Whilst the RCTs looked at potential therapeutic effect of vitamin D in diagnosed MS patients over 6 months-2 years: measured disability (EDSS score) and recorded relapses as outcomes, the MR study took place in the general population and measured the causal effect of genetically predicted lifetime circulating vitamin D concentrations on prevention of MS. The conclusions of MR and RCT studies did not align well, with MR analyses providing evidence for reduced risk of MS conferred by higher vitamin D levels, but no significant therapeutic effect of vitamin D in existing MS was found in the 5 small meta-analyzed trials. Differences which may impact on the ability for MR to complement RCT studies are summarised in Table 3 and discussed below based on this series of case-studies.

For peer review only - http://bmjopen.bmj.com/site/about/guidelines.xhtml

Table 3. Overview of discussed criteria for assessment of alignment of MR and RCT study features.

Match criterion	Issues to consider	Example
Exposure (intervention)	similarity between analyzed exposures	different types of exercise
	intervention intensity	vitamin D dosage
Intervention goal	prevention or treatment	schizophrenia onset or treatment
Outcome (condition)	single or composite outcome	single or composite cardiovascular outcomes
	binary versus categorical outcome	depression or rating on depression assesment scale, such as Hamilton Depression Rating Scale
	similarity between analyzed outcomes	different measures of adiposity
Source population	demographics	young adults or elderly
	health status	diabetic or healthy
Comparator group	exposure-naïve or previously exposed	ex-smoker or never-smoker
	active intervention or placebo	statin as comparator or placebo
Duration of intervention/follow-up	length of intensive intervention and follow-up	short duration of intervention (< 6 months) or long duration and follow-up (>3 years) in RCT and MR
	not uniform intervention intensity or duration	weekly counselling during the initial phase of the trial or throughout

1) Exposure/Intervention

We found that overlapping MR and RCT interventions are often not perfectly identical which may impact on the estimated direction of effect. For example, MR exercise exposures are based on genetic variants associated with self-reported physical activity (moderate-to-vigorous and vigorous)[47,48] in studies assessing the effect on both lipids and bone mineral density (BMD). However, the corresponding RCTs used particular types of exercise, such as walking[49], aerobic exercise[50,51], progressive resistance training[52] and maximal strength training[53] as interventions. While a MR study[48] and two trials[53,54] showed concordant (Figure 4), positive effect of exercise on BMD, we found that the effect of exercise on lipids did not match between MR and RCTs, with MR study[47] reporting null effect and trials generally finding positive effects on HDL-C concentration and negative on LDL-C, total cholesterol and triglycerides blood levels[50–52,55].

Furthermore, intensity of intervention can affect the comparative value of MR and RCT study conclusions. The MR study of vitamin D levels on bone fractures[56] was only able to assess linear effects of the normal range of circulating 25-hydroxyvitamin D concentrations. Consequently, the positive effect of high-concentration vitamin D (≥700 IU daily) on bone fractures in the elderly seen in RCTs[57–59] may not have been accessible in the MR study.

2) Intervention goal

The intervention goal between MR and RCT studies can match (both prevention or treatment) or be misaligned which can potentially impact the ultimate conclusions of the study. We found the latter to be the case for the effect of exercise on schizophrenia. Two MR studies found a null preventative effect of exercise on schizophrenia[60,61], while 3

For peer review only - http://bmjopen.bmj.com/site/about/guidelines.xhtml

meta-analyses of RCTs found a consistent effect of a variety of exercise types on improving total and negative symptoms of schizophrenia[62–64] (Figure 4).

3) Outcome

The short duration of RCTs mean some outcomes (e.g. myocardial infarction) do not accumulate enough events to detect a significant effect, therefore composite measures grouping related diseases are often used. When comparing the effect of systolic blood pressure (SBP) on cardiovascular disease (CVD) outcomes, we found matching conclusions with elevated SBP increasing the risk of CVD both in RCT[65–67] and MR[68–70] studies (Figure 4), with MR studies using both single disease outcomes and a composite outcome. However, MR studies analysing the impact of BMI on cardiovascular disease found reduced adiposity led to reduction in arterial hypertension, CVD and stroke[71,72], which contrasted with the results of one of the biggest RCTs to date. The Look AHEAD RCT in older type 2 diabetics found no preventative effect of weight loss on a composite outcome relating to mortality from cardiovascular causes, non-fatal myocardial infarction, non-fatal stroke or hospitalization for angina[73].

Secondly, RCT outcomes, are often on a quantitative scale measuring symptom strength according to established metrics, (e.g. depressive symptoms on Hamilton Depression Rating Scale[74]). However, the best disease GWAS used to identify MR instruments often represent binary disease outcomes, which could potentially lead to differential conclusions due to reduced power to detect subtler therapeutic effects. While exercise is causally associated with reduced depression and depressive symptoms both in MR[75,76] and RCTs[62,74,77–80], the differences in outcome phenotypes could potentially contribute to

For peer review only - http://bmjopen.bmj.com/site/about/guidelines.xhtml

null MR results[81,82] and positive effect of vitamin D on attenuating eczema symptoms in RCTs[83–86].

4) Source population

MR studies are likely to draw from a wider demographic than RCTs due to use of biobanks and GWAS consortia, while RCTs focus on high risk groups[23]. For example, while in the MR study conducted in general population, there was no strong significant effect of exercise on glycemic markers: HbA1c, fasting glucose and HOMA-IR[87], a significant reduction was found in the meta-analysis of 32 RCTs involving patients with T2D[88].

On the other hand, 5 Mendelian randomization[89–93] studies along with 3 large RCTs[94–96] consistently provide evidence that weight loss is causally associated with reduced risk of T2D (Figure 4), despite MR including the general population and RCTs focussing on at-risk individuals with impaired glucose tolerance.

As another example of possible demographics-driven differences in trial and MR results, MR studies on the relationship between vitamin D levels and atopic dermatitis were conducted in the general population[81,82], while RCTs were conducted separately in children in Mongolia[83] and Boston, USA[84] with winter atopic dermatitis and in adolescent and adult Iranians[85,86].

5) Comparator group

Firstly, due to ethical considerations, trials of harmful behaviours such as alcohol drinking and smoking focus on cessation or reduction in existing users, and do not include never-smokers or never-drinkers as controls, unlike MR studies, which can potentially lead to differences in effect[23]. Nevertheless, the two outcomes analysed here: hypertension for

For peer review only - http://bmjopen.bmj.com/site/about/guidelines.xhtml

alcohol intake and lipids for smoking showed generally congruent results across study types (Supplementary Dataset 4[36]).

Secondly, where it would be unethical to withhold already available efficacious treatments, trials will often include another active intervention in the comparator group[23], for example statins in the trials of effect of PCSK9 inhibitors[97,98] on LDL-C and cardiovascular events. Such RCT design can be mimicked by factorial MR estimating the interaction of multiple exposures, as shown in matching results of the equivalent MR study[16].

6) *Duration of intervention*

While the magnitude of effect seen in trials with long (> 3 years: weight loss to treat hypertension[99,100]/type 2 diabetes[94–96], blood pressure reduction to lower cardiovascular disease risk[65–67]) and short (< 6 months: alcohol intake reduction to lower blood pressure[101–104], exercise to benefit bone mineral density[53]/depression[62,74,78–80,105]) intervention may vary, we find both can result in directional effects consistent with MR results (Figure 4), although with exceptions[73,106].

Triangulation of MR and RCT results

Combining RCT and MR results can offer complimentary evidence on the effectiveness of interventions. Powerful examples include congruence of positive effect of high BMI on hypertension across MR[107–110] and RCT[99,100] studies, high BMI on T2D risk in MR[89–93,111] and RCTs[94–96], and the null effect of vitamin D on various glycemic markers in diverse populations in MR[112], RCTs[113,114] and RCT systematic review[115].

We also found cases, where the majority of studies pointed to one direction of effect, with one MR or RCT identified as an outlier study. In these cases, having a wide array of MR and

For peer review only - http://bmjopen.bmj.com/site/about/guidelines.xhtml

RCT studies (ideally meta-analysed) can be helpful in establishing the likely true causal direction of effect. For instance, 2 MR studies[116,117], a meta-analysis of 5 RCT studies[118] and two RCTs[119,120] indicate no effect of vitamin E on prostate cancer incidence with one outlier RCT[121] showing benefit of vitamin E supplementation in older smokers. Similar contrary findings were found for 1 RCT[122] showing beneficial effect of vitamin D on preventing depressive symptoms, as opposed to null effect in 4 MR studies[123–126] and 2 RCTs[127,128].

On the other hand, MR analyses can show spurious disagreement with the rest of the evidence base. For instance, 2 MR papers[129,130] and a meta-analysis of 16 RCT[130] studies reveal no significant effect of vitamin D on blood pressure in the general population, with the exception of one MR study[131] that indicated a blood pressure-lowering effect of higher vitamin D status. Similarly, a range of study types: one MR analysis[132], one RCT[133] and a meta-analysis of 27 prospective cohorts[134] (only some of them RCTs) confirm a negative impact of smoking on HDL-C levels, bar one MR study showing no significant effect[135]. A series of RCT meta-analyses[136–138] support an effect of coffee consumption (especially unfiltered) on unfavourable blood profile, although this is likely explained by diterpenes[139,140] rather than caffeine, as the latter shows evidence of cardioprotective effects[141]. However, only the recent biggest MR study[140] to date found a significant effect of coffee consumption on LDL-C and total-C levels, unlike two previous smaller MR analyses[142,143], which found a non-significant directionally-consistent relationship.

Discussion

For peer review only - http://bmjopen.bmj.com/site/about/guidelines.xhtml

Our study highlighted that sparsity of data in the electronic databases seriously hampers the ability to automatically parse and compare results of MR and RCT studies. Released for the first time in 2000, ClinicalTrials.Gov is the most comprehensive resource for modern RCT (only <1,000 studies, out of ~167,000 analysed RCTs were started before 2000). Nonetheless, we found that only 11% of all completed RCTs submitted their results to ClinicalTrials.Gov, with median trial start date in 2012. Despite 2007 legislation requiring submission of RCT results to ClinicalTrials.gov within 1 year of completion (with exceptions)[144], only 38% of eligible trials for 2008-2012 submitted their results at any time[145] which rose to 64% for 2018-2019[146]. Furthermore, 60% of studies for failed agents are reported not to be published in peer reviewed journals[147], and in the work presented here we found MeSH annotations were missing from the majority of complex, behavioural and dietary interventions. These factors significantly hamper efforts to systematically triangulate RCT evidence with other studies.

Next, semantic triples describing conclusions of MR and RCT studies automatically extracted from literature abstracts using rule-based methods also had low coverage, with only 25% of MR and 12% of RCT studies associated with >=1 triples. Consequently, we instead decided to focus on a detailed qualitative investigation of a series of case studies to identify the issues associated with triangulating MR and RCT studies

Combining RCT and MR results can offer complimentary evidence on the effectiveness of interventions. Powerful examples include congruence of positive effect of high BMI on hypertension across MR[107–110] and RCT[99,100] studies, high BMI on T2D risk in MR[89–93,111] and RCTs[94–96], and the null effect of vitamin D on various glycemic markers in diverse populations in MR[112], RCTs[113,114] and RCT systematic review[115]. We also

For peer review only - http://bmjopen.bmj.com/site/about/guidelines.xhtml

found cases, where the majority of studies pointed to one direction of effect, with one MR or RCT identified as an outlier study. In these cases, having a wide array of MR and RCT studies (ideally meta-analysed) can be helpful in establishing the likely true causal direction of effect.

Our analysis of genetically predicted effects of perturbation of drug target protein expression on a number of conditions with trials submitted to ClinicalTrials.Gov revealed good concordance with established therapeutics for pQTLs. However, due to the limited number of proteins (n=1,002) and phenotypes (n=225, many non-diseases *per se*) in Zheng et al. (2020)[148], the comparison is necessarily very preliminary. We identify only true positive cases, as false positives and true negatives are difficult to evaluate due to sparsity of drug clinical trial results in ClinicalTrials.Gov/literature[147] and inclusion of non-disease phenotypes in MR analysis. Anecdotally, we found no MR evidence that decreased expression of PLA2G2A leads to reduced cardiovascular disease, which agrees with lack of efficacy of PLA2G2A inhibitor in clinical trials[149–151].

The mixed reliability of eQTL instruments in predicting direction of effect on the outcome could be due to a number of factors such as: less than perfect correlation between mRNA and protein levels[152], hidden pleiotropy in single instruments used in the MR analysis (directly observed for IL2RA)[153], presence of negative feedback loop involved in the drug mechanism[154], translation into protein isoforms with distinct biological effects[155] and differential cell-type specific drug effect[156].

The duration of intervention varies between RCTs and MR studies, with the former spanning no more than the duration of the trial, whilst the latter can represent durations as long as the entire lifetime (although many exposures, such as alcohol intake, will be over a shorter

For peer review only - http://bmjopen.bmj.com/site/about/guidelines.xhtml

time period)[7]. Moreover, intervention in RCTs with long follow-up is not necessarily similarly intensive throughout its duration, or may cease altogether after some time[74,99], i.e. duration of follow-up is longer than duration of intervention in order to allow accumulation of enough events and/or confirm durability of intervention effect. Examples include lifestyle interventions, such as exercise[74] or weight loss programmes[94] like the Look AHEAD trial, with median follow-up of 9.6 years, where group and individual counselling sessions took place weekly in the first 6 months and tapered off over time[73]. That is why our analysis focussed on comparing direction of effect, while ignoring magnitude of effect[157]. However, in certain cases when enough reference data is available, it is feasible to compare MR and RCT effects on the same exposure difference scale[158].

Further impediments to direct comparison between MR and RCTs include differences in outcome definition (composite[65] versus single conditions[70]). Access to rare subpopulations with existing conditions, such as cancer patients receiving specific therapy[159] which are routinely exclusively enrolled into RCTs can be difficult in MR due to the size of GWAS biobanks relative to *N* required for good power.

There are also a number of interventions and outcomes with no single phenotype which could be instrumented with GWAS variants, making MR approaches difficult, although sometimes possible with innovative MR approaches[160]. This is especially true of lifestyle interventions – such as different forms of psychological therapy, complex diet regimens[161] and fasting. Absent or limited heritability of a number of interventions and conditions, such as rehabilitation and traumatic injury makes MR approaches inaccessible.

For peer review only - http://bmjopen.bmj.com/site/about/guidelines.xhtml

For peer review only

The majority of MR studies track the onset rather than progression of disease due to availability of GWAS phenotypes[162] which are often a (binary) single measurement, as opposed to multiple quantitative outcomes frequently measured in RCTs[163]. For that reason, triangulation of MR of onset with RCTs whose intervention is targeting progression of disease, may or may not result in agreement, as seen in our comparison of the effect of exercise on schizophrenia onset/progression (discordant) or depression (concordant) and vitamin D effect on atopic dermatitis onset/progression (discordant).

Many MR studies may be underpowered due to large sample required in indirect estimation[164] as these studies are typically studies of convenience. This bias is less common in RCTs due to pre-registration of study design including power analysis[1], uncommon in MR[165]. Null effect in MR studies may be therefore spurious and not predictive of RCTs for that reason, as seen in two smaller MR studies[142,143] out of three[140] investigating the effect of coffee intake on blood lipids, contrasting with strong clinical trial[136–138] and biochemical evidence[139,140,166].

Furthermore, the presented literature survey used a simple heuristic of reported statistically significant evidence (p-value < 0.05 after multiple testing correction) to compare conclusions across MR and RCT studies, which has well-known limitations[167,168]. Inclusion of the full-spectrum of scaled point-estimates along with their confidence intervals will reveal a more detailed picture in triangulation of MR and RCT evidence (Supplementary Box).

Conclusions

Our research highlights the challenges and benefits of triangulation of MR with RCT evidence. Future efforts, outside of the scope of this work, will focus on fully quantitative

For peer review only - http://bmjopen.bmj.com/site/about/guidelines.xhtml

approaches towards triangulation, involving magnitude of effect size and not just its presence and direction[25]. Developers of such methods will need to be mindful of discrepancies in research hypothesis, duration and intensity of exposure, outcome measures, intervention aim, underlying population characteristics, violations of test assumptions as well as statistical power of the analysis. Furthermore, automated triangulation based on electronic databases requires intensive effort towards structured capture of both MR and RCT study results and associated meta-data, as well as annotation with shared ontologies, which is still challenging using current natural language processing methods, despite constant progress[43,169,170].

List of abbreviations

BMD – Bone mineral density

BMI – Body mass index

CVD – Cardiovascular disease

eQTL – Expression quantitative trait loci

IV – Instrumental variable

MR – Mendelian randomization

MS – Multiple sclerosis

pQTL – Protein quantitative trait loci

SBP – Systolic blood pressure

RCT – Randomized controlled trial

For peer review only - http://bmjopen.bmj.com/site/about/guidelines.xhtml

For peer review only

T2D – Type 2 diabetes

Declarations

Ethics approval statement

This study does not involve human participants and ethical approval was not required.

Data sharing statement

All data generated or analysed during this study are included in this published article and its supplementary information files. Code used to carry out the analysis is available on GitHub: https://github.com/marynias/mr-rct. ClinicalTrials.Gov data was accessed via AACT: https://aact.ctti-clinicaltrials.org/snapshots and analysed data subset is available in Supplementary Datasets 1 & 2. pQTL and eQTL MR analysis results are available via EpigraphDB: https://epigraphdb.org/xqtl

Competing interests

JZ, GDS and TRG receive funding from Biogen for unrelated research. MKS has no competing interests.

Funding information

We would like to acknowledge UK Medical Research Council [mc_uu_00011/4] which provided funding for our work carried out at the University of Bristol MRC Integrative Epidemiology Unit. Our funder played no role in the design of the study, analysis, and interpretation of data and in writing of the manuscript.

Contributorship

For peer review only

TRG conceived and supervised the study as well as acquired funding. MKS curated the data and performed the main analyses. JZ inspired the study and supplied xQTL analyses. MKS, GDS, JZ and TRG wrote the manuscript.

Acknowledgements

Not applicable

For peer review only

References

1 Ference BA, Holmes M V, Smith GD. Using Mendelian Randomization to Improve the Design of Randomized Trials. *Cold Spring Harb Perspect Med* 2021;**11**.http://perspectivesinmedicine.cshlp.org/content/11/7/a040980.abstract

2 Davey Smith G, Ebrahim S. 'Mendelian randomization': can genetic epidemiology contribute to understanding environmental determinants of disease?*. *Int J Epidemiol* 2003;**32**:1–22. doi:10.1093/ije/dyg070

3 Sanderson E, Glymour MM, Holmes M V, *et al.* Mendelian randomization. *Nat Rev Methods Prim* 2022;**2**:6. doi:10.1038/s43586-021-00092-5

4 Hingorani A, Humphries S. Nature's randomised trials. *Lancet* 2005;**366**:1906–8. doi:10.1016/S0140-6736(05)67767-7

5 Davey Smith G, Holmes M V, Davies NM, *et al.* Mendel's laws, Mendelian randomization and causal inference in observational data: substantive and nomenclatural issues. *Eur J Epidemiol* 2020;**35**:99–111. doi:10.1007/s10654-020-00622-7

6 Davies NM, Holmes M V., Davey Smith G. Reading Mendelian randomisation studies: A guide, glossary, and checklist for clinicians. *BMJ* 2018;**362**. doi:10.1136/bmj.k601

7 Swanson SA, Tiemeier H, Ikram MA, *et al.* Nature as a Trialist?: Deconstructing the Analogy Between Mendelian Randomization and Randomized Trials. *Epidemiology* 2017;**28**:653–9. doi:10.1097/EDE.0000000000000699

8 Martin L, Hutchens M, Hawkins C. Trial watch: Clinical trial cycle times continue to increase despite industry efforts. *Nat Rev Drug Discov* 2017;**16**:157. doi:10.1038/nrd.2017.21

9 Egger M, Buitrago-Garcia D, Smith GD. Systematic Reviews of Epidemiological Studies of Etiology and Prevalence. In: Egger M, Higgins JPT, Smith GD, eds. *Systematic Reviews in Health Care: Meta-analyses in Context*. Wiley-Blackwell 2022. Chapter 19.

10 Voight BF, Peloso GM, Orho-Melander M, *et al.* Plasma HDL cholesterol and risk of myocardial infarction: a mendelian randomisation study. *Lancet* 2012;**380**:572–80. doi:https://doi.org/10.1016/S0140-6736(12)60312-2

11 Verdoia M, Schaffer A, Suryapranata H, *et al.* Effects of HDL-modifiers on cardiovascular outcomes: A meta-analysis of randomized trials. *Nutr Metab Cardiovasc Dis* 2015;**25**:9–23. doi:https://doi.org/10.1016/j.numecd.2014.09.003

12 Davey Smith G, Phillips AN. Correlation without a cause: an epidemiological odyssey. *Int J Epidemiol* 2020;**49**:4–14. doi:10.1093/ije/dyaa016

13 Yarmolinsky J, Bonilla C, Haycock PC, *et al.* Circulating Selenium and Prostate Cancer Risk: A Mendelian Randomization Analysis. *JNCI J Natl Cancer Inst* 2018;**110**:1035–8. doi:10.1093/jnci/djy081

14 Lippman SM, Klein EA, Goodman PJ, *et al.* Effect of Selenium and Vitamin E on Risk of Prostate Cancer and Other Cancers: The Selenium and Vitamin E Cancer Prevention Trial (SELECT). *JAMA* 2009;**301**:39–51.

For peer review only - http://bmjopen.bmj.com/site/about/guidelines.xhtml

doi:10.1001/jama.2008.864

15 A. FB, Wonsuk Y, Issa A, *et al.* Effect of Long-Term Exposure to Lower Low-Density Lipoprotein Cholesterol Beginning Early in Life on the Risk of Coronary Heart Disease. *J Am Coll Cardiol* 2012;**60**:2631–9. doi:10.1016/j.jacc.2012.09.017

16 Ference BA, Robinson JG, Brook RD, *et al.* Variation in PCSK9 and HMGCR and Risk of Cardiovascular Disease and Diabetes. *N Engl J Med* 2016;**375**:2144–53. doi:10.1056/NEJMoa1604304

17 Hegele RA. Editorial comment: when Mendelian randomization goes astray. *Curr Opin Lipidol* 2021;**32**.https://journals.lww.com/co-lipidology/Fulltext/2021/04000/Editorial_comment__when_Mendelian_randomization.2.aspx

18 Turner RM, Spiegelhalter DJ, Smith GCS, *et al.* Bias modelling in evidence synthesis. *J R Stat Soc Ser A (Statistics Soc* 2009;**172**:21–47. doi:https://doi.org/10.1111/j.1467-985X.2008.00547.x

19 Morris TT, Heron J, Sanderson E, *et al.* Interpretation of mendelian randomization using one measure of an exposure that varies over time. *medRxiv* Published Online First: 2021. doi:10.1101/2021.11.18.21266515

20 Victora CG, Habicht J-P, Bryce J. Evidence-Based Public Health: Moving Beyond Randomized Trials. *Am J Public Health* 2004;**94**:400–5. doi:10.2105/AJPH.94.3.400

21 Chekroud AM. A Mendelian Randomization Approach for Assessing the Relationship Between Physical Activity and Depression. *JAMA Psychiatry* 2019;**76**:361–2. doi:10.1001/jamapsychiatry.2018.3870

22 Harrison S, Dixon P, Jones HE, *et al.* Long-term cost-effectiveness of interventions for obesity: A mendelian randomisation study. *PLOS Med* 2021;**18**:e1003725.https://doi.org/10.1371/journal.pmed.1003725

23 Holmes M V, Richardson TG, Ference BA, *et al.* Integrating genomics with biomarkers and therapeutic targets to invigorate cardiovascular drug development. *Nat Rev Cardiol* 2021;**18**:435–53. doi:10.1038/s41569-020-00493-1

24 Borén J, Chapman MJ, Krauss RM, *et al.* Low-density lipoproteins cause atherosclerotic cardiovascular disease: pathophysiological, genetic, and therapeutic insights: a consensus statement from the European Atherosclerosis Society Consensus Panel. *Eur Heart J* 2020;**41**:2313–30. doi:10.1093/eurheartj/ehz962

25 Munafò MR, Higgins JPT, Smith GD. Triangulating Evidence through the Inclusion of Genetically Informed Designs. *Cold Spring Harb Perspect Med* 2021;**11**. doi:10.1101/cshperspect.a040659

26 Lawlor DA, Tilling K, Smith GD. Triangulation in aetiological epidemiology. *Int J Epidemiol* 2016;**45**:1866–86. doi:10.1093/ije/dyw314

27 Zarin DA, Tse T, Williams RJ, *et al.* The ClinicalTrials.gov Results Database — Update and Key Issues. *N Engl J Med* 2011;**364**:852–60. doi:10.1056/NEJMsa1012065

28 Liu Y, Elsworth B, Erola P, *et al.* EpiGraphDB: a database and data mining platform for health data science. *Bioinformatics* 2020;:0–0.https://academic.oup.com/bioinformatics/advance-article/doi/10.1093/bioinformatics/btaa961/5962087

29 Tasneem A, Aberle L, Ananth H, *et al.* The Database for Aggregate Analysis of ClinicalTrials.gov (AACT) and Subsequent Regrouping by Clinical Specialty. *PLoS One* 2012;**7**:e33677.https://doi.org/10.1371/journal.pone.0033677

30 Tenaerts P, Madre L, Archdeacon P, *et al.* The Clinical Trials Transformation Initiative: innovation through collaboration. *Nat Rev Drug Discov* 2014;**13**:797–8. doi:10.1038/nrd4442

31 Ochoa D, Hercules A, Carmona M, *et al.* Open Targets Platform: supporting systematic drug–target identification and prioritisation. *Nucleic Acids Res* 2020;**49**:D1302–10. doi:10.1093/nar/gkaa1027

32 Wishart DS, Feunang YD, Guo AC, *et al.* DrugBank 5.0: a major update to the DrugBank database for 2018. *Nucleic Acids Res* 2017;**46**:D1074–82. doi:10.1093/nar/gkx1037

33 Kilicoglu H, Shin D, Fiszman M, *et al.* SemMedDB: a PubMed-scale repository of biomedical semantic predications. *Bioinformatics* 2012;**28**:3158–60. doi:10.1093/bioinformatics/bts591

34 Elsworth B, Gaunt TR. MELODI Presto: a fast and agile tool to explore semantic triples derived from biomedical literature. *Bioinformatics* 2020;**37**:583–5. doi:10.1093/bioinformatics/btaa726

35 Fine LJ, Philogene GS, Gramling R, *et al.* Prevalence of multiple chronic disease risk factors: 2001 National Health Interview Survey. *Am J Prev Med* 2004;**27**:18–24. doi:https://doi.org/10.1016/j.amepre.2004.04.017

For peer review only

For peer review only - http://bmjopen.bmj.com/site/about/guidelines.xhtml

[dataset] 36 Sobczyk MK, Zhang J, Davey Smith G, *et al.* Supplementary Datasets for Systematic comparison of Mendelian randomization studies and randomized controlled trials using electronic databases. Zenodo. 2022. doi:10.5281/zenodo.7437032

37 Zheng J, Haberland V, Baird D, *et al.* Phenome-wide Mendelian randomization mapping the influence of the plasma proteome on complex diseases. *Nat Genet* 2020;**52**:1122–31. doi:10.1038/s41588-020-0682-6

38 Cicero AFG, Tartagni E, Ertek S. Safety and tolerability of injectable lipid-lowering drugs: a review of available clinical data. *Expert Opin Drug Saf* 2014;**13**:1023–30. doi:10.1517/14740338.2014.932348

39 Luo J, Wu S-J, Lacy ER, *et al.* Structural Basis for the Dual Recognition of IL-12 and IL-23 by Ustekinumab. *J Mol Biol* 2010;**402**:797–812. doi:https://doi.org/10.1016/j.jmb.2010.07.046

40 Hansen RA, Gartlehner G, Webb AP, *et al.* Efficacy and safety of donepezil, galantamine, and rivastigmine for the treatment of Alzheimer's disease: a systematic review and meta-analysis. *Clin Interv Aging* 2008;**3**:211–25.https://pubmed.ncbi.nlm.nih.gov/18686744

41 Ference BA, Kastelein JJP, Ginsberg HN, *et al.* Association of Genetic Variants Related to CETP Inhibitors and Statins With Lipoprotein Levels and Cardiovascular Risk. *JAMA* 2017;**318**:947–56. doi:10.1001/jama.2017.11467

42 Piepho RW. Overview of the angiotensin-converting-enzyme inhibitors. *Am J Heal Pharm* 2000;**57**:S3–7. doi:10.1093/ajhp/57.suppl_1.S3

43 Marshall IJ, Nye B, Kuiper J, *et al.* Trialstreamer: A living, automatically updated database of clinical trial reports. *J Am Med Informatics Assoc* 2020;**27**:1903–12. doi:10.1093/jamia/ocaa163

44 Mokry LE, Ross S, Ahmad OS, *et al.* Vitamin D and Risk of Multiple Sclerosis: A Mendelian Randomization Study. *PLOS Med* 2015;**12**:e1001866.https://doi.org/10.1371/journal.pmed.1001866

45 James E, Dobson R, Kuhle J, *et al.* The effect of vitamin D-related interventions on multiple sclerosis relapses: a meta-analysis. *Mult Scler J* 2013;**19**:1571–9. doi:10.1177/1352458513489756

46 Hempel S, Graham GD, Fu N, *et al.* A systematic review of the effects of modifiable risk factor interventions on the progression of multiple sclerosis. *Mult Scler J* 2017;**23**:513–24. doi:10.1177/1352458517690271

47 Zhuang Z, Gao M, Yang R, *et al.* Association of physical activity, sedentary behaviours and sleep duration with cardiovascular diseases and lipid profiles: a Mendelian randomization analysis. *Lipids Health Dis* 2020;**19**:86. doi:10.1186/s12944-020-01257-z

48 Xu F, Zhang Q, Wang L-K, *et al.* Estimates of the effects of physical activity on osteoporosis using multivariable Mendelian randomization analysis. *Osteoporos Int* 2021;**32**:1359–67. doi:10.1007/s00198-020-05786-2

49 Kelley GA, Kelley KS, Tran ZV. Walking, lipids, and lipoproteins: a meta-analysis of randomized controlled trials. *Prev Med (Baltim)* 2004;**38**:651–61. doi:https://doi.org/10.1016/j.ypmed.2003.12.012

50 Kelley GA, Kelley KS. Aerobic exercise and lipids and lipoproteins in men: a meta-analysis of randomized controlled trials. *J Men's Heal Gend* 2006;**3**:61–70. doi:10.1016/j.jmhg.2005.09.003

51 Kelley GA, Kelley KS, Tran ZV. Aerobic Exercise and Lipids and Lipoproteins in Women: A Meta-Analysis of Randomized Controlled Trials. *J Women's Heal* 2004;**13**:1148–64. doi:10.1089/jwh.2004.13.1148

52 Kelley GA, Kelley KS. Impact of progressive resistance training on lipids and lipoproteins in adults: A meta-analysis of randomized controlled trials. *Prev Med (Baltim)* 2009;**48**:9–19. doi:https://doi.org/10.1016/j.ypmed.2008.10.010

53 Mosti MP, Carlsen T, Aas E, *et al.* Maximal Strength Training Improves Bone Mineral Density and Neuromuscular Performance in Young Adult Women. *J Strength Cond Res* 2014;**28**.https://journals.lww.com/nsca-jscr/Fulltext/2014/10000/Maximal_Strength_Training_Improves_Bone_Mineral.30.aspx

54 Allison SJ, Folland JP, Rennie WJ, *et al.* High impact exercise increased femoral neck bone mineral density in older men: A randomised unilateral intervention. *Bone* 2013;**53**:321–8. doi:https://doi.org/10.1016/j.bone.2012.12.045

55 Katzel LI, Bleecker ER, Colman EG, *et al.* Effects of weight loss vs aerobic exercise training on risk factors for coronary disease in healthy, obese, middle-aged and older men. A randomized controlled trial. *JAMA* 1995;**274**:1915–21. doi:10.1001/jama.1995.03530240025035

56 Çolak Y, Afzal S, Nordestgaard BG. 25-Hydroxyvitamin D and Risk of Osteoporotic Fractures: Mendelian Randomization Analysis in 2 Large Population-Based Cohorts. *Clin Chem* 2020;**66**:676–85.

For peer review only - http://bmjopen.bmj.com/site/about/guidelines.xhtml

doi:10.1093/clinchem/hvaa049

57 Bischoff-Ferrari HA, Willett WC, Orav EJ, *et al.* A Pooled Analysis of Vitamin D Dose Requirements for Fracture Prevention. *N Engl J Med* 2012;**367**:40–9. doi:10.1056/NEJMoa1109617

58 Bischoff-Ferrari HA, Willett WC, Wong JB, *et al.* Prevention of Nonvertebral Fractures With Oral Vitamin D and Dose Dependency: A Meta-analysis of Randomized Controlled Trials. *Arch Intern Med* 2009;**169**:551–61. doi:10.1001/archinternmed.2008.600

59 Bischoff-Ferrari HA, Willett WC, Wong JB, *et al.* Fracture Prevention With Vitamin D SupplementationA Meta-analysis of Randomized Controlled Trials. *JAMA* 2005;**293**:2257–64. doi:10.1001/jama.293.18.2257

60 Papiol S, Schmitt A, Maurus I, *et al.* Association Between Physical Activity and Schizophrenia: Results of a 2-Sample Mendelian Randomization Analysis. *JAMA Psychiatry* 2021;**78**:441–4. doi:10.1001/jamapsychiatry.2020.3946

61 Sun H, Gao X, Que X, *et al.* The causal relationships of device-measured physical activity with bipolar disorder and schizophrenia in adults: A 2-Sample mendelian randomization study. *J Affect Disord* 2020;**263**:598–604. doi:https://doi.org/10.1016/j.jad.2019.11.034

62 Rosenbaum S, Tiedemann A, Sherrington C, *et al.* Physical activity interventions for people with mental illness: A systematic review and meta-analysis. J. Clin. Psychiatry. 2014;**75**:964–74. doi:10.4088/JCP.13r08765

63 Firth J, Cotter J, Elliott R, *et al.* A systematic review and meta-analysis of exercise interventions in schizophrenia patients. *Psychol Med* 2015;**45**:1343–1361. doi:10.1017/S0033291714003110

64 Sabe M, Kaiser S, Sentissi O. Physical exercise for negative symptoms of schizophrenia: Systematic review of randomized controlled trials and meta-analysis. *Gen Hosp Psychiatry* 2020;**62**:13–20. doi:https://doi.org/10.1016/j.genhosppsych.2019.11.002

65 Williamson JD, Supiano MA, Applegate WB, *et al.* Intensive vs Standard Blood Pressure Control and Cardiovascular Disease Outcomes in Adults Aged ≥75 Years: A Randomized Clinical Trial. *JAMA* 2016;**315**:2673–82. doi:10.1001/jama.2016.7050

66 Brunström M, Carlberg B. Association of Blood Pressure Lowering With Mortality and Cardiovascular Disease Across Blood Pressure Levels: A Systematic Review and Meta-analysis. *JAMA Intern Med* 2018;**178**:28–36. doi:10.1001/jamainternmed.2017.6015

67 Rahimi K, Bidel Z, Nazarzadeh M, *et al.* Age-stratified and blood-pressure-stratified effects of blood-pressure-lowering pharmacotherapy for the prevention of cardiovascular disease and death: an individual participant-level data meta-analysis. *Lancet* 2021;**398**:1053–64. doi:10.1016/S0140-6736(21)01921-8

68 Gill D, Georgakis MK, Zuber V, *et al.* Genetically Predicted Midlife Blood Pressure and Coronary Artery Disease Risk: Mendelian Randomization Analysis. *J Am Heart Assoc* 2020;**9**:e016773. doi:10.1161/JAHA.120.016773

69 Malik R, Georgakis MK, Vujkovic M, *et al.* Relationship Between Blood Pressure and Incident Cardiovascular Disease: Linear and Nonlinear Mendelian Randomization Analyses. *Hypertension* 2021;**77**:2004–13. doi:10.1161/HYPERTENSIONAHA.120.16534

70 Wan EYF, Fung WT, Schooling CM, *et al.* Blood Pressure and Risk of Cardiovascular Disease in UK Biobank. *Hypertension* 2021;**77**:367–75. doi:10.1161/HYPERTENSIONAHA.120.16138

71 Riaz H, Khan MS, Siddiqi TJ, *et al.* Association Between Obesity and Cardiovascular Outcomes: A Systematic Review and Meta-analysis of Mendelian Randomization Studies. *JAMA Netw Open* 2018;**1**:e183788–e183788. doi:10.1001/jamanetworkopen.2018.3788

72 Larsson SC, Bäck M, Rees JMB, *et al.* Body mass index and body composition in relation to 14 cardiovascular conditions in UK Biobank: a Mendelian randomization study. *Eur Heart J* 2020;**41**:221–6. doi:10.1093/eurheartj/ehz388

73 Cardiovascular Effects of Intensive Lifestyle Intervention in Type 2 Diabetes. *N Engl J Med* 2013;**369**:145–54. doi:10.1056/NEJMoa1212914

74 Mather AS, Rodriguez C, Guthrie MF, *et al.* Effects of exercise on depressive symptoms in older adults with poorly responsive depressive disorder: Randomised controlled trial. *Br J Psychiatry* 2002;**180**:411–415. doi:10.1192/bjp.180.5.411

75 Choi KW, Chen C-Y, Stein MB, *et al.* Assessment of Bidirectional Relationships Between Physical Activity and Depression Among Adults: A 2-Sample Mendelian Randomization Study. *JAMA Psychiatry* 2019;**76**:399–408.

For peer review only

For peer review only - http://bmjopen.bmj.com/site/about/guidelines.xhtml

doi:10.1001/jamapsychiatry.2018.4175

76 Choi KW, Stein MB, Nishimi KM, *et al.* An Exposure-Wide and Mendelian Randomization Approach to Identifying Modifiable Factors for the Prevention of Depression. *Am J Psychiatry* 2020;**177**:944–54. doi:10.1176/appi.ajp.2020.19111158

77 Veale D, Le Fevre K, Pantelis C, *et al.* Aerobic exercise in the adjunctive treatment of depression: a randomized controlled trial. *J R Soc Med* 1992;**85**:541–4.https://pubmed.ncbi.nlm.nih.gov/1433121

78 Schuch FB, Vancampfort D, Richards J, *et al.* Exercise as a treatment for depression: A meta-analysis adjusting for publication bias. *J Psychiatr Res* 2016;**77**:42–51. doi:https://doi.org/10.1016/j.jpsychires.2016.02.023

79 Kvam S, Kleppe CL, Nordhus IH, *et al.* Exercise as a treatment for depression: A meta-analysis. *J Affect Disord* 2016;**202**:67–86. doi:https://doi.org/10.1016/j.jad.2016.03.063

80 Gordon BR, McDowell CP, Hallgren M, *et al.* Association of Efficacy of Resistance Exercise Training With Depressive Symptoms: Meta-analysis and Meta-regression Analysis of Randomized Clinical Trials. *JAMA Psychiatry* 2018;**75**:566–76. doi:10.1001/jamapsychiatry.2018.0572

81 Manousaki D, Paternoster L, Standl M, *et al.* Vitamin D levels and susceptibility to asthma, elevated immunoglobulin E levels, and atopic dermatitis: A Mendelian randomization study. *PLOS Med* 2017;**14**:1–16. doi:10.1371/journal.pmed.1002294

82 Drodge DR, Budu-Aggrey A, Paternoster L. Causal Analysis Shows Evidence of Atopic Dermatitis Leading to an Increase in Vitamin D Levels. *J Invest Dermatol* 2021;**141**:1339–41. doi:https://doi.org/10.1016/j.jid.2020.09.013

83 Camargo CA, Ganmaa D, Sidbury R, *et al.* Randomized trial of vitamin D supplementation for winter-related atopic dermatitis in children. *J Allergy Clin Immunol* 2014;**134**:831-835.e1. doi:https://doi.org/10.1016/j.jaci.2014.08.002

84 Sidbury R, Sullivan AF, Thadhani RI, *et al.* Randomized controlled trial of vitamin D supplementation for winter-related atopic dermatitis in Boston: a pilot study. *Br J Dermatol* 2008;**159**:245—247. doi:10.1111/j.1365-2133.2008.08601.x

85 Amestejani M, Salehi BS, Vasigh M, *et al.* Vitamin D supplementation in the treatment of atopic dermatitis: a clinical trial study. *J Drugs Dermatology* 2012;**11**.

86 Javanbakht MH, Keshavarz SA, Djalali M, *et al.* Randomized controlled trial using vitamins E and D supplementation in atopic dermatitis. *J Dermatolog Treat* 2011;**22**:144–50. doi:10.3109/09546630903578566

87 Meisinger C, Linseisen J, Leitzmann M, *et al.* Association of physical activity and sedentary behavior with type 2 diabetes and glycemic traits: a two-sample Mendelian randomization study. *BMJ Open Diabetes Res Care* 2020;**8**. doi:10.1136/bmjdrc-2020-001896

88 Shah SZA, Karam JA, Zeb A, *et al.* Movement is Improvement: The Therapeutic Effects of Exercise and General Physical Activity on Glycemic Control in Patients with Type 2 Diabetes Mellitus: A Systematic Review and Meta-Analysis of Randomized Controlled Trials. *Diabetes Ther* 2021;**12**:707–32. doi:10.1007/s13300-021-01005-1

89 Corbin LJ, Richmond RC, Wade KH, *et al.* BMI as a Modifiable Risk Factor for Type 2 Diabetes: Refining and Understanding Causal Estimates Using Mendelian Randomization. *Diabetes* 2016;**65**:3002–7. doi:10.2337/db16-0418

90 Dale CE, Fatemifar G, Palmer TM, *et al.* Causal Associations of Adiposity and Body Fat Distribution With Coronary Heart Disease, Stroke Subtypes, and Type 2 Diabetes Mellitus. *Circulation* 2017;**135**:2373–88. doi:10.1161/CIRCULATIONAHA.116.026560

91 Cheng L, Zhuang H, Ju H, *et al.* Exposing the causal effect of body mass index on the risk of type 2 diabetes mellitus: a mendelian randomization study. *Front Genet* 2019;**10**:94.

92 Wainberg M, Mahajan A, Kundaje A, *et al.* Homogeneity in the association of body mass index with type 2 diabetes across the UK Biobank: A Mendelian randomization study. *PLOS Med* 2019;**16**:e1002982.https://doi.org/10.1371/journal.pmed.1002982

93 Xu H, Jin C, Guan Q. Causal Effects of Overall and Abdominal Obesity on Insulin Resistance and the Risk of Type 2 Diabetes Mellitus: A Two-Sample Mendelian Randomization Study. *Front Genet* 2020;**11**:603. doi:10.3389/fgene.2020.00603

94 Pan X-R, Li G-W, Hu Y-H, *et al.* Effects of Diet and Exercise in Preventing NIDDM in People With Impaired Glucose Tolerance: The Da Qing IGT and Diabetes Study. *Diabetes Care* 1997;**20**:537–44. doi:10.2337/diacare.20.4.537

For peer review only - http://bmjopen.bmj.com/site/about/guidelines.xhtml

95 Tuomilehto J, Lindström J, Eriksson JG, *et al.* Prevention of Type 2 Diabetes Mellitus by Changes in Lifestyle among Subjects with Impaired Glucose Tolerance. *N Engl J Med* 2001;**344**:1343–50. doi:10.1056/NEJM200105033441801

96 Reduction in the Incidence of Type 2 Diabetes with Lifestyle Intervention or Metformin. *N Engl J Med* 2002;**346**:393–403. doi:10.1056/NEJMoa012512

97 Sabatine MS, Giugliano RP, Wiviott SD, *et al.* Efficacy and Safety of Evolocumab in Reducing Lipids and Cardiovascular Events. *N Engl J Med* 2015;**372**:1500–9. doi:10.1056/NEJMoa1500858

98 Sabatine MS, Giugliano RP, Keech AC, *et al.* Evolocumab and Clinical Outcomes in Patients with Cardiovascular Disease. *N Engl J Med* 2017;**376**:1713–22. doi:10.1056/NEJMoa1615664

99 He J, Whelton PK, Appel LJ, *et al.* Long-Term Effects of Weight Loss and Dietary Sodium Reduction on Incidence of Hypertension. *Hypertension* 2000;**35**:544–9. doi:10.1161/01.HYP.35.2.544

100 Long-Term Weight Loss and Changes in Blood Pressure: Results of the Trials of Hypertension Prevention, Phase II. *Ann Intern Med* 2001;**134**:1–11. doi:10.7326/0003-4819-134-1-200101020-00007

101 Puddey IB, Beilin LJ, Vandongen R, *et al.* Evidence for a direct effect of alcohol consumption on blood pressure in normotensive men. A randomized controlled trial. *Hypertension* 1985;**7**:707–13. doi:10.1161/01.HYP.7.5.707

102 Puddey IB, Beilin LJ, Vandongen R. REGULAR ALCOHOL USE RAISES BLOOD PRESSURE IN TREATED HYPERTENSIVE SUBJECTS: A Randomised Controlled Trial. *Lancet* 1987;**329**:647–51. doi:https://doi.org/10.1016/S0140-6736(87)90413-2

103 Xin X, He J, Frontini MG, *et al.* Effects of Alcohol Reduction on Blood Pressure. *Hypertension* 2001;**38**:1112–7. doi:10.1161/hy1101.093424

104 Puddey IB, Parker M, Beilin LJ, *et al.* Effects of alcohol and caloric restrictions on blood pressure and serum lipids in overweight men. *Hypertension* 1992;**20**:533–41. doi:10.1161/01.HYP.20.4.533

105 Helgadóttir B, Hallgren M, Ekblom Ö, *et al.* Training fast or slow? Exercise for depression: A randomized controlled trial. *Prev Med (Baltim)* 2016;**91**:123–31. doi:https://doi.org/10.1016/j.ypmed.2016.08.011

106 Ma C, Avenell A, Bolland M, *et al.* Effects of weight loss interventions for adults who are obese on mortality, cardiovascular disease, and cancer: systematic review and meta-analysis. *BMJ* 2017;**359**. doi:10.1136/bmj.j4849

107 Timpson NJ, Harbord R, Davey Smith G, *et al.* Does Greater Adiposity Increase Blood Pressure and Hypertension Risk? *Hypertension* 2009;**54**:84–90. doi:10.1161/HYPERTENSIONAHA.109.130005

108 Lyall DM, Celis-Morales C, Ward J, *et al.* Association of Body Mass Index With Cardiometabolic Disease in the UK Biobank: A Mendelian Randomization Study. *JAMA Cardiol* 2017;**2**:882–9. doi:10.1001/jamacardio.2016.5804

109 Lee M-R, Lim Y-H, Hong Y-C. Causal association of body mass index with hypertension using a Mendelian randomization design. *Medicine (Baltimore)* 2018;**97**:e11252–e11252. doi:10.1097/MD.0000000000011252

110 van Oort S, Beulens JWJ, van Ballegooijen AJ, *et al.* Association of Cardiovascular Risk Factors and Lifestyle Behaviors With Hypertension. *Hypertension* 2020;**76**:1971–9. doi:10.1161/HYPERTENSIONAHA.120.15761

111 Rivera CM, Ren B. Mapping human epigenomes. *Cell* 2013;**155**:39–55. doi:10.1016/j.cell.2013.09.011

112 Wang N, Wang C, Chen X, *et al.* Vitamin D, prediabetes and type 2 diabetes: bidirectional Mendelian randomization analysis. *Eur J Nutr* 2020;**59**:1379–88. doi:10.1007/s00394-019-01990-x

113 Salehpour A, Shidfar F, Hosseinpanah F, *et al.* Does vitamin D3 supplementation improve glucose homeostasis in overweight or obese women? A double-blind, randomized, placebo-controlled clinical trial. *Diabet Med* 2013;**30**:1477–81. doi:https://doi.org/10.1111/dme.12273

114 Sollid ST, Hutchinson MYS, Fuskev\r ag OM, *et al.* No Effect of High-Dose Vitamin D Supplementation on Glycemic Status or Cardiovascular Risk Factors in Subjects With Prediabetes. *Diabetes Care* 2014;**37**:2123–31. doi:10.2337/dc14-0218

115 Zuk A, Fitzpatrick T, Rosella LC. Effect of Vitamin D3 Supplementation on Inflammatory Markers and Glycemic Measures among Overweight or Obese Adults: A Systematic Review of Randomized Controlled Trials. *PLoS One* 2016;**11**:e0154215.https://doi.org/10.1371/journal.pone.0154215

116 Wright ME, Peters U, Gunter MJ, *et al.* Association of Variants in Two Vitamin E Transport Genes with Circulating Vitamin E Concentrations and Prostate Cancer Risk. *Cancer Res* 2009;**69**:1429–38. doi:10.1158/0008-5472.CAN-08-2343

For peer review only - http://bmjopen.bmj.com/site/about/guidelines.xhtml

For peer review only

117 Cheng W-W, Wang Z-K, Shangguan H-F, *et al.* Are vitamins relevant to cancer risks? A Mendelian randomization investigation. *Nutrition* 2020;**78**:110870. doi:https://doi.org/10.1016/j.nut.2020.110870

118 Jiang L, Yang K, Tian J, *et al.* Efficacy of Antioxidant Vitamins and Selenium Supplement in Prostate Cancer Prevention: A Meta-Analysis of Randomized Controlled Trials. *Nutr Cancer* 2010;**62**:719–27. doi:10.1080/01635581.2010.494335

119 Gaziano JM, Glynn RJ, Christen WG, *et al.* Vitamins E and C in the Prevention of Prostate and Total Cancer in Men: The Physicians' Health Study II Randomized Controlled Trial. *JAMA* 2009;**301**:52–62. doi:10.1001/jama.2008.862

120 Wang L, Sesso HD, Glynn RJ, *et al.* Vitamin E and C supplementation and risk of cancer in men: posttrial follow-up in the Physicians' Health Study II randomized trial. *Am J Clin Nutr* 2014;**100**:915–23. doi:10.3945/ajcn.114.085480

121 Heinonen OP, Koss L, Albanes D, *et al.* Prostate Cancer and Supplementation With α-Tocopherol and β-Carotene: Incidence and Mortality in a Controlled Trial. *JNCI J Natl Cancer Inst* 1998;**90**:440–6. doi:10.1093/jnci/90.6.440

122 Vieth R, Kimball S, Hu A, *et al.* Randomized comparison of the effects of the vitamin D3adequate intake versus 100 mcg (4000 IU) per day on biochemical responses and the wellbeing of patients. *Nutr J* 2004;**3**:8. doi:10.1186/1475-2891-3-8

123 Michaëlsson K, Melhus H, Larsson SC. Serum 25-Hydroxyvitamin D Concentrations and Major Depression: A Mendelian Randomization Study. *Nutrients* 2018;**10**. doi:10.3390/nu10121987

124 Libuda L, Laabs B-H, Ludwig C, *et al.* Vitamin D and the Risk of Depression: A Causal Relationship? Findings from a Mendelian Randomization Study. *Nutrients* 2019;**11**. doi:10.3390/nu11051085

125 Mulugeta A, Lumsden A, Hyppönen E. Relationship between Serum 25(OH)D and Depression: Causal Evidence from a Bi-Directional Mendelian Randomization Study. *Nutrients* 2021;**13**. doi:10.3390/nu13010109

126 Milaneschi Y, Peyrot WJ, Nivard MG, *et al.* A role for vitamin D and omega-3 fatty acids in major depression? An exploration using genomics. *Transl Psychiatry* 2019;**9**:219. doi:10.1038/s41398-019-0554-y

127 Bertone-Johnson ER, Powers SI, Spangler L, *et al.* Vitamin D Supplementation and Depression in the Women's Health Initiative Calcium and Vitamin D Trial. *Am J Epidemiol* 2012;**176**:1–13. doi:10.1093/aje/kwr482

128 Kjærgaard M, Waterloo K, Wang CEA, *et al.* Effect of vitamin D supplement on depression scores in people with low levels of serum 25-hydroxyvitamin D: nested case—control study and randomised clinical trial. *Br J Psychiatry* 2012;**201**:360–368. doi:10.1192/bjp.bp.111.104349

129 Skaaby T, Husemoen LLN, Martinussen T, *et al.* Vitamin D Status, Filaggrin Genotype, and Cardiovascular Risk Factors: A Mendelian Randomization Approach. *PLoS One* 2013;**8**:e57647.https://doi.org/10.1371/journal.pone.0057647

130 Kunutsor SK, Burgess S, Munroe PB, *et al.* Vitamin D and high blood pressure: causal association or epiphenomenon? *Eur J Epidemiol* 2014;**29**:1–14. doi:10.1007/s10654-013-9874-z

131 Vimaleswaran KS, Cavadino A, Berry DJ, *et al.* Association of vitamin D status with arterial blood pressure and hypertension risk: a mendelian randomisation study. *Lancet Diabetes Endocrinol* 2014;**2**:719–29. doi:https://doi.org/10.1016/S2213-8587(14)70113-5

132 Rosoff DB, Davey Smith G, Mehta N, *et al.* Evaluating the relationship between alcohol consumption, tobacco use, and cardiovascular disease: A multivariable Mendelian randomization study. *PLOS Med* 2020;**17**:e1003410.https://doi.org/10.1371/journal.pmed.1003410

133 Gepner AD, Piper ME, Johnson HM, *et al.* Effects of smoking and smoking cessation on lipids and lipoproteins: Outcomes from a randomized clinical trial. *Am Heart J* 2011;**161**:145–51. doi:https://doi.org/10.1016/j.ahj.2010.09.023

134 Maeda K, Noguchi Y, Fukui T. The effects of cessation from cigarette smoking on the lipid and lipoprotein profiles: a meta-analysis. *Prev Med (Baltim)* 2003;**37**:283–90. doi:https://doi.org/10.1016/S0091-7435(03)00110-5

135 Åsvold BO, Bjørngaard JH, Carslake D, *et al.* Causal associations of tobacco smoking with cardiovascular risk factors: a Mendelian randomization analysis of the HUNT Study in Norway. *Int J Epidemiol* 2014;**43**:1458–70. doi:10.1093/ije/dyu113

136 Jee SH, He J, Appel LJ, *et al.* Coffee Consumption and Serum Lipids: A Meta-Analysis of Randomized Controlled Clinical Trials. *Am J Epidemiol* 2001;**153**:353–62. doi:10.1093/aje/153.4.353

For peer review only - http://bmjopen.bmj.com/site/about/guidelines.xhtml

137 Cai L, Ma D, Zhang Y, *et al.* The effect of coffee consumption on serum lipids: a meta-analysis of randomized controlled trials. *Eur J Clin Nutr* 2012;**66**:872–7. doi:10.1038/ejcn.2012.68

138 Du Y, Lv Y, Zha W, *et al.* Effect of coffee consumption on dyslipidemia: A meta-analysis of randomized controlled trials. *Nutr Metab Cardiovasc Dis* 2020;**30**:2159–70. doi:https://doi.org/10.1016/j.numecd.2020.08.017

139 Urgert R, Katan MB. THE CHOLESTEROL-RAISING FACTOR FROM COFFEE BEANS. *Annu Rev Nutr* 1997;**17**:305–24. doi:10.1146/annurev.nutr.17.1.305

140 Zhou A, Hyppönen E. Habitual coffee intake and plasma lipid profile: Evidence from UK Biobank. *Clin Nutr* 2021;**40**:4404–13. doi:https://doi.org/10.1016/j.clnu.2020.12.042

141 Lebeau PF, Byun JH, Platko K, *et al.* Caffeine blocks SREBP2-induced hepatic PCSK9 expression to enhance LDLR-mediated cholesterol clearance. *Nat Commun* 2022;**13**:770. doi:10.1038/s41467-022-28240-9

142 Kwok MK, Leung GM, Schooling CM. Habitual coffee consumption and risk of type 2 diabetes, ischemic heart disease, depression and Alzheimer's disease: a Mendelian randomization study. *Sci Rep* 2016;**6**:36500. doi:10.1038/srep36500

143 Nordestgaard AT, Thomsen M, Nordestgaard BG. Coffee intake and risk of obesity, metabolic syndrome and type 2 diabetes: a Mendelian randomization study. *Int J Epidemiol* 2015;**44**:551–65. doi:10.1093/ije/dyv083

144 Zarin DA, Tse T, Williams RJ, *et al.* Trial Reporting in ClinicalTrials.gov — The Final Rule. *N Engl J Med* 2016;**375**:1998–2004. doi:10.1056/NEJMsr1611785

145 Anderson ML, Chiswell K, Peterson ED, *et al.* Compliance with Results Reporting at ClinicalTrials.gov. *N Engl J Med* 2015;**372**:1031–9. doi:10.1056/NEJMsa1409364

146 DeVito NJ, Bacon S, Goldacre B. Compliance with legal requirement to report clinical trial results on ClinicalTrials.gov: a cohort study. *Lancet* 2020;**395**:361–9. doi:10.1016/S0140-6736(19)33220-9

147 Hwang TJ, Carpenter D, Lauffenburger JC, *et al.* Failure of Investigational Drugs in Late-Stage Clinical Development and Publication of Trial Results. *JAMA Intern Med* 2016;**176**:1826–33. doi:10.1001/jamainternmed.2016.6008

148 Giri A, Hellwege JN, Keaton JM, *et al.* Trans-ethnic association study of blood pressure determinants in over 750,000 individuals. *Nat Genet* 2019;**51**:51–62. doi:10.1038/s41588-018-0303-9

149 Holmes M V, Simon T, Exeter HJ, *et al.* Secretory Phospholipase A_2-IIA and Cardiovascular Disease. *J Am Coll Cardiol* 2013;**62**:1966–76. doi:10.1016/j.jacc.2013.06.044

150 Rosenson RS, Hislop C, Elliott M, *et al.* Effects of varespladib methyl on biomarkers and major cardiovascular events in acute coronary syndrome patients. *J Am Coll Cardiol* 2010;**56**:1079–88. doi:10.1016/j.jacc.2010.06.015

151 Nicholls SJ, Kastelein JJP, Schwartz GG, *et al.* Varespladib and cardiovascular events in patients with an acute coronary syndrome: the VISTA-16 randomized clinical trial. *JAMA* 2014;**311**:252–62. doi:10.1001/jama.2013.282836

152 Liu Y, Beyer A, Aebersold R. On the Dependency of Cellular Protein Levels on mRNA Abundance. *Cell* 2016;**165**:535–50. doi:https://doi.org/10.1016/j.cell.2016.03.014

153 Verbanck M, Chen C-Y, Neale B, *et al.* Detection of widespread horizontal pleiotropy in causal relationships inferred from Mendelian randomization between complex traits and diseases. *Nat Genet* 2018;**50**:693–8. doi:10.1038/s41588-018-0099-7

154 Foulds CE. Disrupting a negative feedback loop drives endocrine therapy-resistant breast cancer. *Proc Natl Acad Sci* 2018;**115**:8236–8. doi:10.1073/pnas.1811263115

155 Cupido AJ, Asselbergs FW, Natarajan P, *et al.* Dissecting the IL-6 pathway in cardiometabolic disease: A Mendelian randomization study on both IL6 and IL6R. *Br J Clin Pharmacol*;**n/a**. doi:https://doi.org/10.1111/bcp.15191

156 Gallo MA, Kaufman D. Antagonistic and agonistic effects of tamoxifen: significance in human cancer. *Semin Oncol* 1997;**24**:S1—71—S1—80.http://europepmc.org/abstract/MED/9045319

157 Ference BA. How to use Mendelian randomization to anticipate the results of randomized trials. *Eur Heart J* 2017;**39**:360–2. doi:10.1093/eurheartj/ehx462

158 Holmes M V, Smith GD. Revealing the effect of CETP inhibition in cardiovascular disease. *Nat Rev Cardiol* 2017;**14**:635–6. doi:10.1038/nrcardio.2017.156

For peer review only - http://bmjopen.bmj.com/site/about/guidelines.xhtml

159 Kearns AE, Northfelt DW, Dueck AC, *et al.* Osteoporosis prevention in prostate cancer patients receiving androgen ablation therapy: placebo-controlled double-blind study of estradiol and risedronate: N01C8. *Support Care Cancer* 2010;**18**:321–8. doi:10.1007/s00520-009-0655-x

160 Li J, Guasch-Ferré M, Chung W, *et al.* The Mediterranean diet, plasma metabolome, and cardiovascular disease risk. *Eur Heart J* 2020;**41**:2645–56. doi:10.1093/eurheartj/ehaa209

161 Verfürden ML, Gilbert R, Lucas A, *et al.* Effect of nutritionally modified infant formula on academic performance: linkage of seven dormant randomised controlled trials to national education data. *BMJ* 2021;**375**:e065805. doi:10.1136/bmj-2021-065805

162 Paternoster L, Tilling K, Davey Smith G. Genetic epidemiology and Mendelian randomization for informing disease therapeutics: Conceptual and methodological challenges. *PLOS Genet* 2017;**13**:e1006944.https://doi.org/10.1371/journal.pgen.1006944

163 Stubbs B, Vancampfort D, Hallgren M, *et al.* EPA guidance on physical activity as a treatment for severe mental illness: a meta-review of the evidence and Position Statement from the European Psychiatric Association (EPA), supported by the International Organization of Physical Therapists in Mental . *Eur Psychiatry* 2018;**54**:124–44. doi:DOI: 10.1016/j.eurpsy.2018.07.004

164 Schooling CM, Freeman G, Cowling BJ. Mendelian Randomization and Estimation of Treatment Efficacy for Chronic Diseases. *Am J Epidemiol* 2013;**177**:1128–33. doi:10.1093/aje/kws344

165 Lor GCY, Risch HA, Fung WT, *et al.* Reporting and guidelines for mendelian randomization analysis: A systematic review of oncological studies. *Cancer Epidemiol* 2019;**62**:101577. doi:https://doi.org/10.1016/j.canep.2019.101577

166 Poole R, Kennedy OJ, Roderick P, *et al.* Coffee consumption and health: umbrella review of meta-analyses of multiple health outcomes. *BMJ* 2017;**359**. doi:10.1136/bmj.j5024

167 Altman DG, Bland JM. Statistics notes: Absence of evidence is not evidence of absence. *BMJ* 1995;**311**:485. doi:10.1136/bmj.311.7003.485

168 Sterne JAC, Smith GD. Sifting the evidence—what's wrong with significance tests? *Phys Ther* 2001;**81**:1464–9. doi:10.1093/ptj/81.8.1464

169 Nye BE, DeYoung J, Lehman E, *et al.* Understanding Clinical Trial Reports: Extracting Medical Entities and Their Relations. *AMIA Jt Summits Transl Sci proceedings AMIA Jt Summits Transl Sci* 2021;**2021**:485–94.https://pubmed.ncbi.nlm.nih.gov/34457164

170 Whitton J, Hunter A. Automated tabulation of clinical trial results: A joint entity and relation extraction approach with transformer-based language representations. 2021.

For peer review only

For peer review only - http://bmjopen.bmj.com/site/about/guidelines.xhtml

Figure legends

Figure 1. **Comparison of Mendelian Randomization and Randomized Controlled Trial design.** After: Nitsch *et al.* (2006), Ebrahim & Smith (2008), Ference (2018).

Figure 2. Filtering steps applied to ClinicalTrials.Gov database. Filtering was designed to identify RCT trials whose final results statistics were uploaded to the database (*Main dataset*). In addition, other RCTs which published their findings in scientific journals were identified (*Literature dataset*).

Figure 3. Popularity of MR and RCT studies over time. We compare counts of MR and RCT papers indexed by PubMed (solid lines) with number of semantic triples derived from them using SemMedDb (dashed lines).

Figure 4. Summary of case series of MR and RCT studies with matching exposures (interventions) and outcomes (conditions). The values correspond to the number of analysed studies in a given category, while the cell background colour indicates summary direction of effect on the outcome when exposure is increased – we report direction of effect found either in all analysed studies or their majority (>50%).

For peer review only - http://bmjopen.bmj.com/site/about/guidelines.xhtml

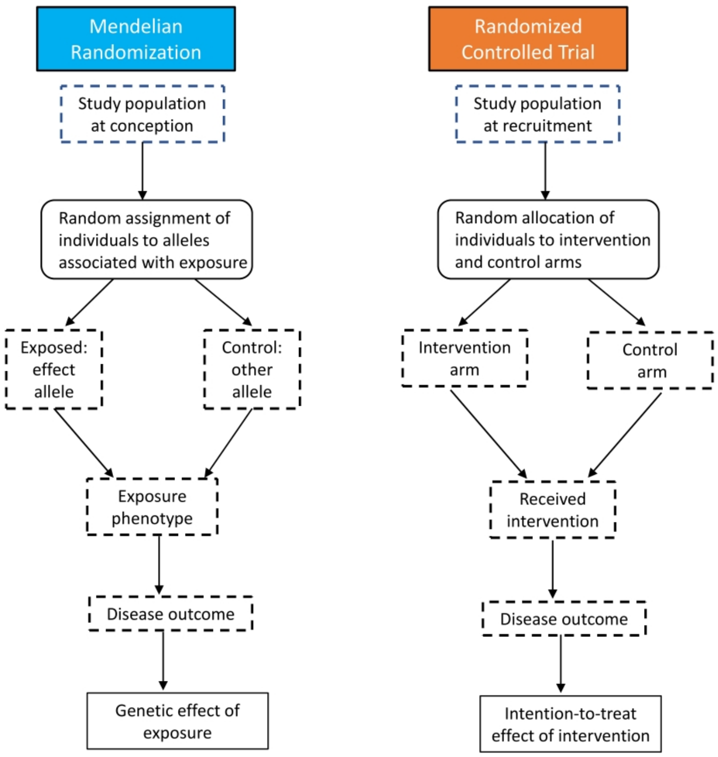

Comparison of Mendelian Randomization and Randomized Controlled Trial design. After: Nitsch et al. (2006), Ebrahim & Smith (2008), Ference (2018).

484x699mm (236 x 236 DPI)

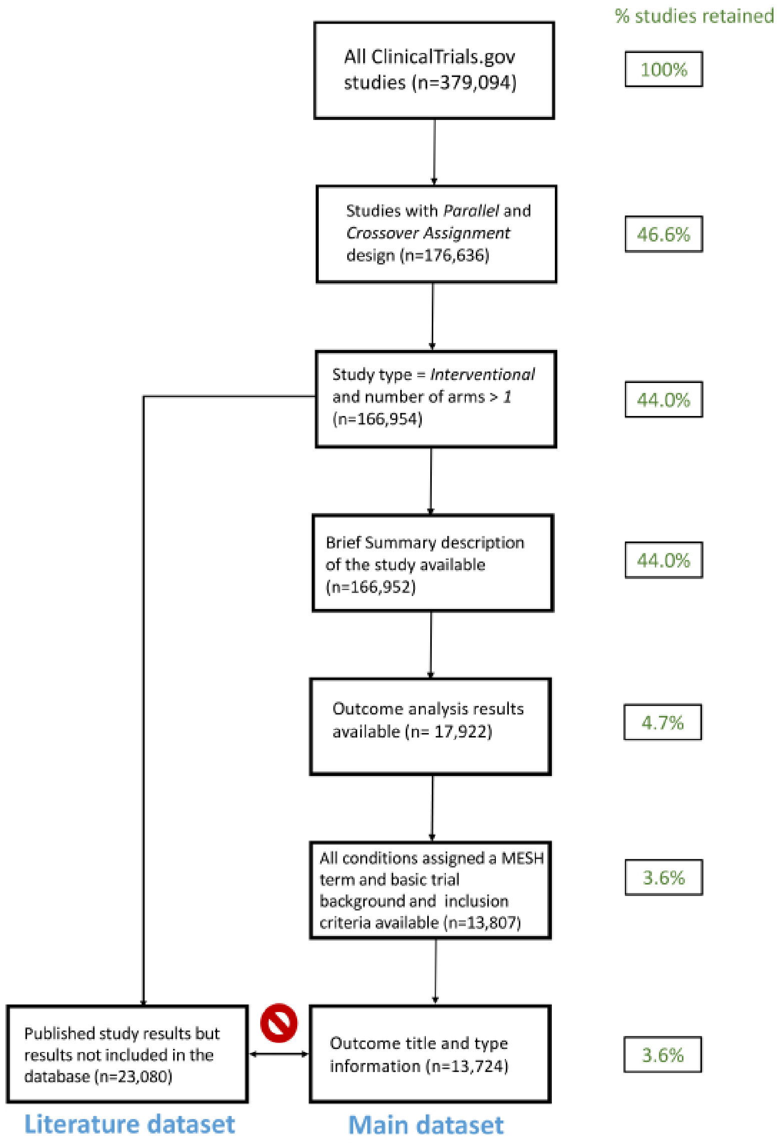

Filtering steps applied to ClinicalTrials.Gov database. Filtering was designed to identify RCT trials whose final results statistics were uploaded to the database (Main dataset). In addition, other RCTs which published their findings in scientific journals were identified (Literature dataset).

67x90mm (236 x 236 DPI)

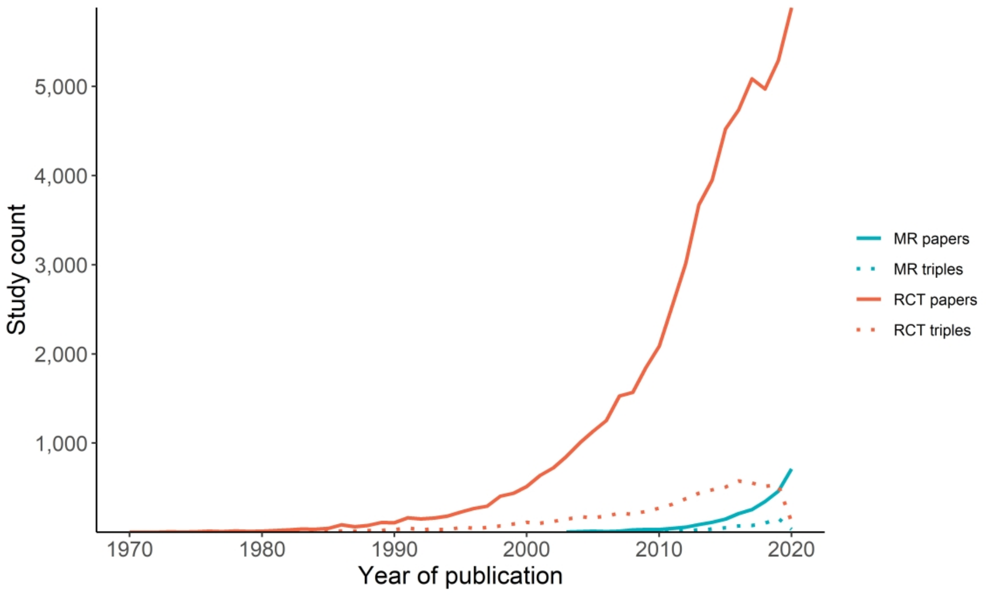

Popularity of MR and RCT studies over time. We compare counts of MR and RCT papers indexed by PubMed (solid lines) with number of semantic triples derived from them using SemMedDb (dashed lines).

508x305mm (236 x 236 DPI)

exposure	outcome	MR	RCT
alcohol	hypertension	2	4
blood pressure	cardiovascular disease	3	3
body mass index	cardiovascular disease	2	2
body mass index	hypertension	4	3
body mass index	type 2 diabetes	5	3
coffee	glycemic biomarkers	1	2
coffee	lipids	3	3
diary intake	blood pressure	1	1
exercise	bone mineral density	1	2
exercise	depression	2	7
exercise	glycemic biomarkers	1	2
exercise	lipids	1	6
exercise	pain	1	1
exercise	schizophrenia	2	3
smoking	lipids	2	2
vitamin D	atopic dermatitis	2	4
vitamin D	blood pressure	3	2
vitamin D	body mass index	2	2
vitamin D	bone fractures	1	5
vitamin D	bone mineral density	3	2
vitamin D	cardiovascular disease	1	2
vitamin D	depression	4	3
vitamin D	glycemic biomarkers	1	3
vitamin D	lipids	3	4
vitamin D	multiple sclerosis	1	2
vitamin E	prostate cancer	2	4

Legend

- all positive effect size
- majority positive effect size
- all null
- majority null
- all negative effect size
- majority negative effect size

Summary of case series of MR and RCT studies with matching exposures (interventions) and outcomes (conditions). The values correspond to the number of analysed studies in a given category, while the cell background colour indicates summary direction of effect on the outcome when exposure is increased – we report direction of effect found either in all analysed studies or their majority (>50%).

484x861mm (236 x 236 DPI)

Supplementary Box

It is anticipated that the longer duration of exposure differences seen in MR studies will generate larger effect sizes than will relatively short-term modification of the exposure in RCTs. For example, the genetic variants related to non-HDL cholesterol (henceforth "cholesterol", the target of cholesterol lowering drugs such as the statins) have been shown to relate to relatively stable differences from early childhood to late adulthood, thus generating a lifetime of differential exposure to circulating cholesterol. The RCTs of cholesterol lowering drugs designed to show effects on coronary heart disease (CHD) events last ~5 years. Atherosclerosis is a disease process that develops from childhood onwards, and the CHD it generates would not be expected to be abolished by a few years of cholesterol lowering in middle age or older (the usual age included in the RCTs). As there are several cholesterol lowering drugs which target different genes it is possible to compare MR studies using genetic variants in those genes that are robustly related to cholesterol with RCTs of drugs that target those genes. Figure SB1 below combines data from such MR analyses with the RCTs.

As anticipated the RCTs produce about 40% of the risk reduction seen with a lifetime difference in exposure levels[1]. This scaling of the effects predicted from MR studies and seen in the matching trials can be applied to MR/RCT comparisons for other exposures, as the time course of effects being produced may be quicker than seen in the case of cholesterol, or take longer, or indeed there may be no effect in the RCTs if the effect of the exposure acts during a critical period in earlier life and sets in train a disease process that is not reversible by later modification of the exposure[2].

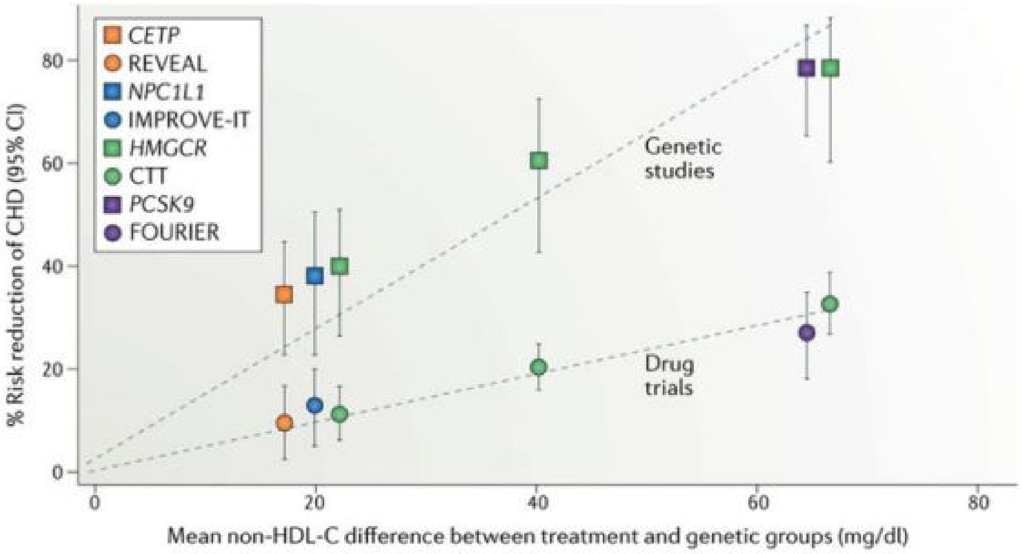

Figure SB1. Drug treatment (circles) and genetic proxy (squares) effects on reducing cholesterol levels and the corresponding reduction in risk of from matching drug RCTs and MR analyses. The colours indicate the gene from which variants are taken in the MR studies and the target of the drug used in the named RCTs. *Abbreviations: CETP, cholesteryl ester transfer protein; HMGCR, 3-hydroxy-3-methylglutaryl-CoA reductase; NPC1L1, Niemann-Pick C1-like protein 1; PCSK9, proprotein convertase subtilisin/kexin type 9.* Figure reproduced with permission from[3].

References

1. Smith GD, Ebrahim S. Mendelian randomization: prospects, potentials, and limitations. Int J Epidemiol. 2004;33: 30–42. doi:10.1093/ije/dyh132

2. Holmes M V, Ala-Korpela M, Smith GD. Mendelian randomization in cardiometabolic disease: challenges in evaluating causality. Nat Rev Cardiol. 2017;14: 577–590. doi:10.1038/nrcardio.2017.78

3. Holmes M V, Smith GD. Revealing the effect of CETP inhibition in cardiovascular disease. Nat Rev Cardiol. 2017;14: 635–636. doi:10.1038/nrcardio.2017.156

For peer review only

Supplementary Note

ClinicalTrials.Gov *Data filtering*

We only included studies with most common designs suitable for comparison with published MR studies: *Parallel Assignment* and *Crossover Assignment* (eliminating: *Single Group Assignment, Sequential Assignment, Factorial Assignment*) intervention model in the Designs table with *Randomized* allocation to allow for selection of RCT. We further used the study type field (=*Interventional*) with a minimum number of arms = *2* in the Studies table as an additional filtering criterion to arrive at a set of RCT studies. The next stage of filtering concerned background information and study results. We first filtered on the presence of a study description in the Brief Summaries table. The key criterion was then presence of results in the Outcome Analyses table, where we selected the variables: *param_type, param_value, p_value* and *method.* Next, we needed all conditions to have at least 1 Medical Subject Headings (MeSH) term assigned in all_conditions view to facilitate automatic comparison with external data sources. We dropped that requirement for interventions, as especially behavioural interventions could not be assigned a MeSH term (see Results). Finally, a range of basic reference and eligibility criteria were required: *brief_title, study_type, overall_status, phase, number_of_arms, enrolment* in the Studies table, *gender, criteria* in the Eligibilities table and *outcome title* and *type* in the Outcomes table.

Additionally, we extracted a subset of studies which did not supply any results in the Outcome Analyses table and therefore did not contribute to the *Main* dataset above but were RCT studies with published literature records in the Study References table (*reference type* = result).

Overview of top SemMed triples for MR and RCT studies

An overview of the top subjects in MR (Figure S3a) and RCTs (Figure S3b) revealed a high number of terms related to adiposity (*obesity, body mass index, adiponectin*), lipid biology (*lipoproteins, hydroxymethylglutaryl-CoA reductase inhibitors, PCKSK9, high density lipoproteins*) as well as *type 2 diabetes* and *vitamin D*. Terms related to type 2 diabetes (*insulin, metformin*) and *vitamin D* were also found in RCT triples. As expected by the preponderance of drug interventions in ClinicalTrials.Gov, there was a noticeable bias towards pharmaceutical preparations amongst the top 10 subjects in RCT triples with terms such as *methotrexate*, *aspirin*, *clomiphene citrate*, *dexamethasone*, *prednisolone* and *cyclosporine.* SemMedDb identified *associated with* as a top predicate in MR studies (Figure S3c), followed by *predisposes*, *coexists with* and *affects* which underlines the skew of current MR studies towards identifying risk factors for disease. On the other hand, RCT studies lean towards identifying treatments (Figure S3d) which is reflected in the top 1 predicate *treats* (*n*= 4,732, second-best *coexists with* n= 1,495). Among the top objects in MR studies (Figure S3e), we found cardiovascular diseases (*coronary arteriosclerosis*, *coronary heart disease*, *myocardial ischemia/infarction*, *hypertensive disease*, *ischemic stroke*). High frequency of a smaller number of cardiovascular disease terms (Figure S3f) was also found among RCT objects (*hypertensive disease, cardiovascular disease*). Both MR and

For peer review only

RCT showed type 2 diabetes and obesity as commonly studied conditions. RCT objects included also two infectious diseases: malaria and hepatitis C.

For peer review only

Supplementary Figures

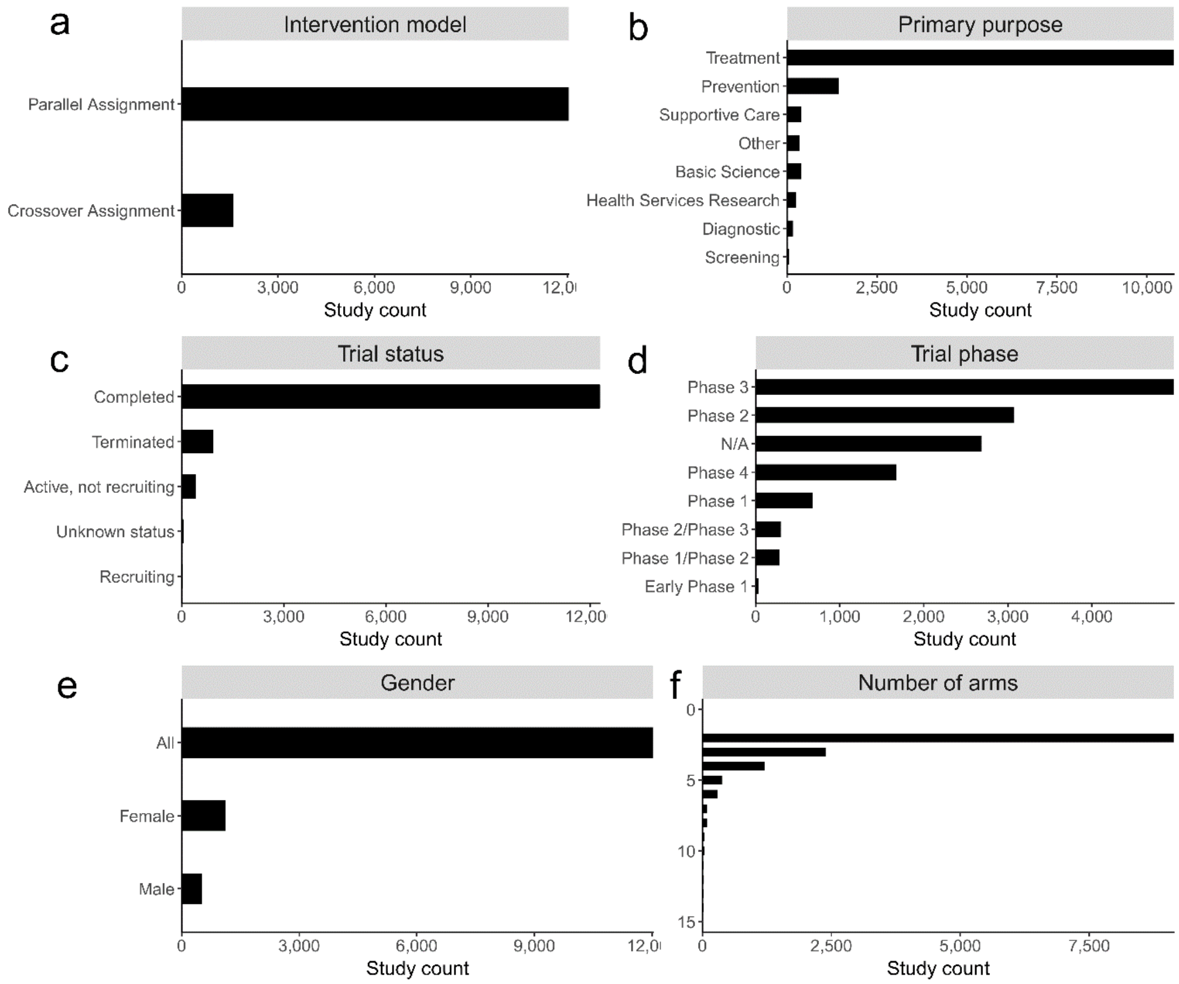

Figure S1. Overview of RCT general features in the Main dataset by their frequency: a) intervention model, b) primary purpose, c) trial status, d) trial phase, e) gender, f) number of arms.

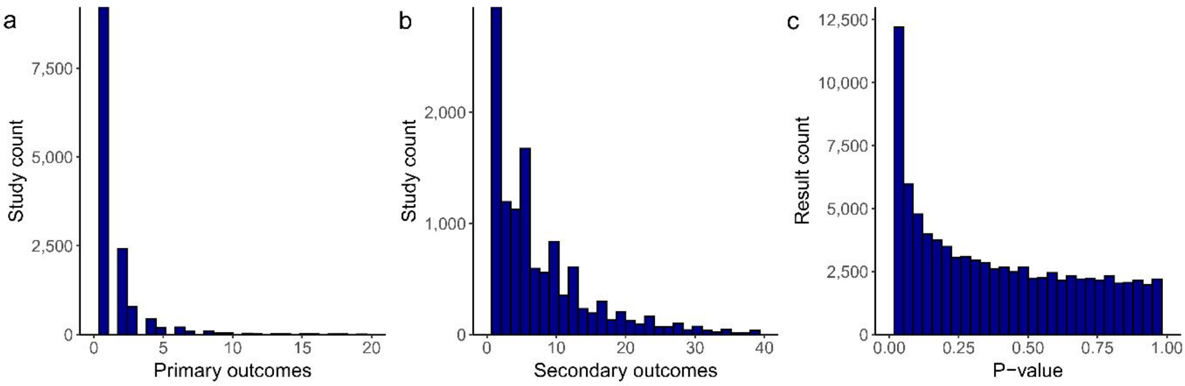

Figure S2. Distribution of: a) primary outcome number among RCT studies, b) secondary outcome number among RCT studies, c) P-value among study results.

For peer review only

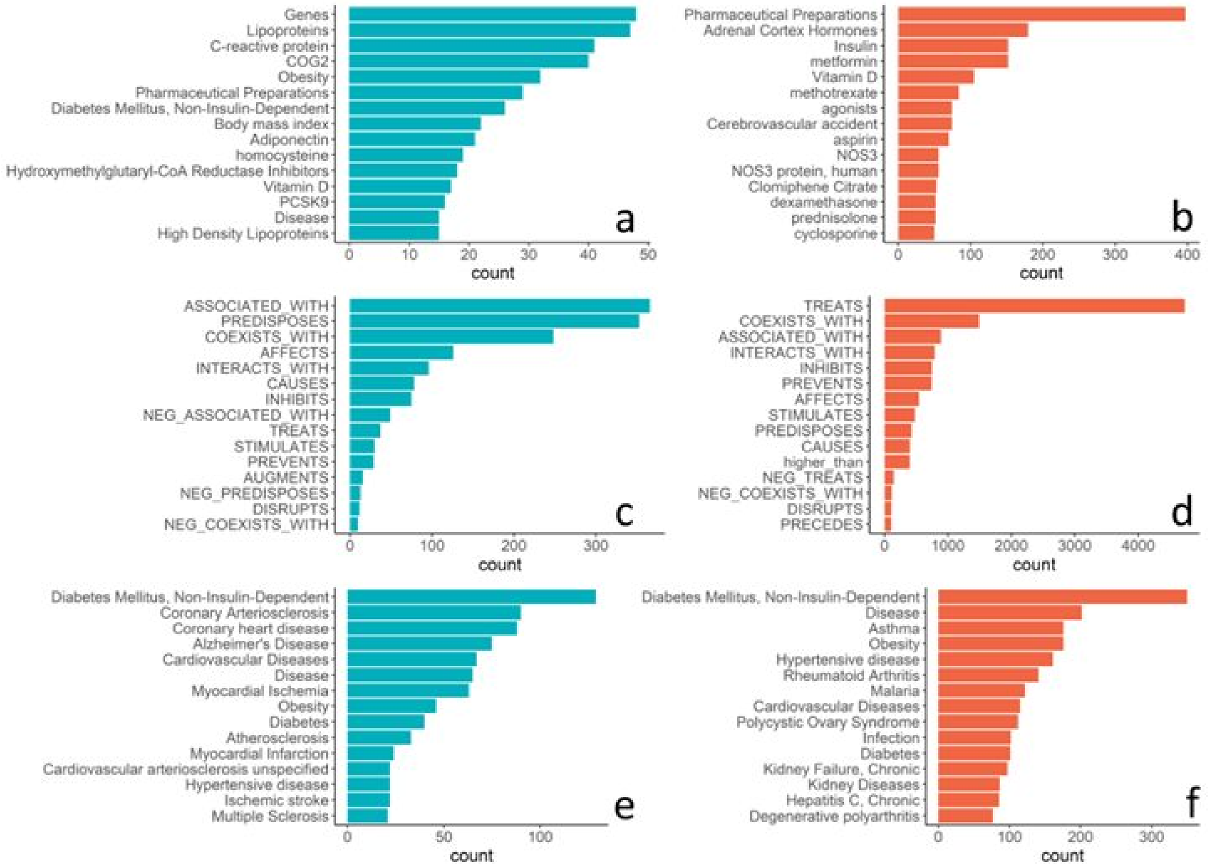

Figure S3. Overview of SemMedDb semantic triples derived from MR (blue, left-hand panels) and RCT (orange, right-hand panels) studies: a) & b) top 15 subjects, c) & d) top 15 predicates, d) & e) top 15 objects.

Supplementary Materials legends

Table S2. Drug target-disease matches supported by evidence from MR (blood eQTL instruments, https://epigraphdb.org/xqtl) and RCT studies (*Main dataset* from ClinicalTrials.Gov).

Table S3. Matching subject-object pairs between MR and RCT studies in semantic triples identified by SemMedDb.

Table S4. Matching subjects between MR and RCT studies in semantic triples identified by SemMedDb.

Table S5. Matching objects between MR and RCT studies in semantic triples identified by SemMedDb.

Table S6. Sample comparison of MR and RCT studies with matching exposures and outcomes across a range of criteria.

Supplementary Dataset 1. Select ClinicalTrials.Gov data fields for the RCT studies identified as the *main dataset*.

Supplementary Dataset 2. Select ClinicalTrials.Gov data fields for the RCT studies identified as the *literature dataset*.

Supplementary Dataset 3. Comparison of frequency of RCT general features between the *Main dataset* and background of all RCTs available in ClinicalTrials.Gov.

Supplementary Dataset 4. Case series of MR and RCT studies with matching exposures (interventions) and outcomes (conditions).

For peer review only

Intervention	Main	Main %	Literature	Literature %
Drug	10,187	62.8%	9,554	38.9%
Other	1,705	10.5%	3,668	14.9%
Biological	1,289	7.9%	1,468	6.0%
Behavioral	1,013	6.2%	2,427	9.9%
Device	1,209	7.5%	2,472	10.1%
Procedure	436	2.7%	2,952	12.0%
Dietary Supplement	215	1.3%	662	2.7%
Radiation	109	0.7%	672	2.7%
Genetic	19	0.1%	117	0.5%
Combination Product	24	0.1%	71	0.3%
Diagnostic Test	11	0.1%	501	2.0%

For peer review only

MR Exposure	RCT Drug Intervention	MR Outcomes	RCT Conditions
CETP	EVACETRAPIB, ANACETRAPIB	LDL cholesterol || id:300, HDL cholesterol || id:299, Total cholesterol || id:301, Triglycerides || id:302	Hyperlipidemia, Dyslipidemia, Hypercholesterolemia, Hyperlipoproteinemia Type II
HMGCR	ATORVASTATIN, PRAVASTATIN, SIMVASTATIN	LDL cholesterol || id:300, Total cholesterol || id:301, Non-cancer illness code, self-reported: high cholesterol || id:UKB-b:10912	Hyperlipidemia, Dyslipidemia, Hypercholesterolemia, Mixed Dyslipidemia etc.
GUCY1A3 and GUCY1B3	RIOCIGUAT	Diastolic blood pressure automated reading || id:UKB-a:359, Vascular/heart problems diagnosed by doctor: High blood pressure || id:UKB-b:14177	Pulmonary Hypertension, Pulmonary Arterial Hypertension
IGF1R	MECASERMIN	Height || id:89	Growth Disorders|Insulin-Like Growth Factor-1 Deficiency
IL2RA	DACLIZUMAB	Type 1 diabetes || id:285	Diabetes Mellitus, Type 1
KCNJ11	GLIMEPIRIDE	Diabetes diagnosed by doctor || id:UKB-b:10753	Diabetes Mellitus, Type 2, Diabetes, Pre-diabetes
ACE	ENALAPRIL, LISINOPRIL, PERINDOPRIL	Vascular/heart problems diagnosed by doctor: High blood pressure || id:UKB-b:14177	Hypertension, Essential Hypertension, Hypertension, Pulmonary|Ventricular Dysfunction, Left
PDE3A	ANAGRELIDE	Platelet count || id:1008	Thrombocythemia
CD86	ABATACEPT	Multiple sclerosis || id:1025	Multiple Sclerosis, Relapsing-Remitting
ESR1	FULVESTRANT, TAMOXIFEN	Breast cancer (Combined Oncoarray; iCOGS; GWAS meta analysis) || id:1126	Breast Cancer, Estrogen Receptor-positive (ER+) Breast Cancer etc.

For peer review only

ESR1	ESTRADIOL, POLYESTRADIOL PHOSPHATE	Femoral neck bone mineral density || id:980, Lumbar spine bone mineral density || id:982, Heel bone mineral density (BMD) T-score automated || id:UKB-a:500	Bone Mineral Density
PARP3	OLAPARIB, VELIPARIB, RUCAPARIB	Ovarian cancer || id:1120	Ovarian Neoplasms, Ovarian Cancer etc.
MS4A1	OCRELIZUMAB, OFATUMUMAB, RITUXIMAB	Rheumatoid arthritis || id:833, Type 1 Diabetes Mellitus	Rheumatoid Arthritis, Type 1 Diabetes Mellitus
JAK2	TOFACITINIB	Crohn's disease || id:12, Inflammatory bowel disease || id:294	Ulcerative Colitis, Crohn's Disease
TYK2	TOFACITINIB, PEFICITINIB, UPADACITINIB	Rheumatoid arthritis || id:833, Psoriasis || id:282	Rheumatoid arthritis, Psoriasis

For peer review only

Matching trials	Concordant direction of effect?	xQTL	Notes
7	Yes	eQTL	
50	Yes	eQTL	
7	Yes	eQTL	Main drug target is GUCY1A2
1	No	eQTL	
1	No - no significant effect seen in RCT	eQTL	Opposite direction of effect seen depending on the 2 instruments used in MR. This suggests hidden pleiotropy in at least one of the variants. Expect to see inhibition of IL2RA resulting in less disease based on direction seen in multiple sclerosis (main drug indication).
63	Yes	eQTL	
3	Yes	eQTL	
1	Yes	eQTL	
1	No - no significant effect seen in RCT	eQTL	
9	Yes*	eQTL	*RCTs focussed on use of tamoxifen as a control, and not intervention so no effect observed. MR estimate is in agreement with tissue-specific agonist and antagonist effect of tamoxifen (Gallo and Kaufman 1997) on estrogen receptor (agonist observed in MR).

For peer review only

1	No*	eQTL	*RCT, carried out in adolescents, found no significant effect. MR estimate is in disagreement with agonist role on estrogen receptor leading to increase in bone mineral density, which is found in published RCTs in the literature (Ettinger et al. 2004).
7	No	eQTL	
10	No	eQTL	
6	No	eQTL	
34	No	eQTL	tofacitinib moderately inhibits TYK2, the main drug targets are JAK1, JAK2 and JAK3

For peer review only

subject	object	MR
Lipoproteins	Cardiovascular Diseases	8
Lipoproteins	Coronary heart disease	4
Obesity	Diabetes Mellitus, Non-Insulin-Dependent	4
C-reactive protein	Diabetes Mellitus, Non-Insulin-Dependent	3
High Density Lipoproteins	Coronary Arteriosclerosis	3
Pharmaceutical Preparations	Cardiovascular Diseases	3
Vitamin D	Disease	3
Diabetes Mellitus, Non-Insulin-Dependent	Disease	3
Disease	Diabetes Mellitus, Non-Insulin-Dependent	3
Insulin	Obesity	3
Diabetes Mellitus, Non-Insulin-Dependent	Diabetes	3
Insulin	Diabetes Mellitus, Non-Insulin-Dependent	3
Adiponectin	Diabetes Mellitus, Non-Insulin-Dependent	2
Amino Acids, Branched-Chain	Diabetes Mellitus, Non-Insulin-Dependent	2
Asthma	Obesity	2
Cardiovascular Diseases	Disease	2
Diabetic Nephropathy	Diabetes Mellitus, Insulin-Dependent	2
LPA	Coronary heart disease	2
testosterone	cardiovascular risk factor	2
vitamin B12	Body mass index	2
ethanol	Disease	2
homocysteine	Alzheimer's Disease	2
Vitamin D	Vitamin D Deficiency	2
Obesity	Asthma	2
Diabetes Mellitus, Non-Insulin-Dependent	Cardiovascular Diseases	2
Pharmaceutical Preparations	Disease	2
Pharmaceutical Preparations	Diabetes	2
Alanine Transaminase	Alkaline Phosphatase	1
Amino Acids, Branched-Chain	Insulin	1
Anti-Inflammatory Agents	Disease	1
Anticoagulants	Myocardial Ischemia	1
APOE	Alzheimer's Disease	1
Apolipoprotein A-I	Coronary heart disease	1
Apolipoprotein E	Alzheimer's Disease	1
Atherosclerosis	Cardiovascular Diseases	1
Atherosclerosis	Diabetes Mellitus, Non-Insulin-Dependent	1
ATP Citrate (pro-S)-Lyase	Low-Density Lipoproteins	1
beta carotene	Diabetes Mellitus, Non-Insulin-Dependent	1
Body mass index	Diabetes	1
C-reactive protein	Vitamin D	1
Cardiovascular Diseases	Coronary heart disease	1
cardiovascular risk factor	Atherosclerosis	1
Cerebrovascular accident	Cardiovascular Diseases	1
coenzyme Q10	Disease	1
Complement System Proteins	Disease	1
Coronary heart disease	Obesity	1
Crohn Disease	Disease	1
DEFA1A3	Periodontitis	1
Disease	Cerebrovascular accident	1
Disease	Fatty Liver	1
Disease	Liver diseases	1

For peer review only

Enzymes	Vitamin D	1
ethanol	Cardiovascular Diseases	1
Free thyroxin	thyrotropin	1
Genes	Dyslipidemias	1
Genes	interleukin-6	1
homocysteine	Cerebrovascular accident	1
Hydroxymethylglutaryl-CoA Reductase Inhibitor:	Atherosclerosis	1
Hydroxymethylglutaryl-CoA Reductase Inhibitor:	Coronary heart disease	1
Hyperglycemia	Diabetes Mellitus, Non-Insulin-Dependent	1
Hyperlipidemia	Disease	1
Inflammatory Bowel Diseases	Crohn Disease	1
Insulin	Myocardial Infarction	1
interleukin-6	Coronary heart disease	1
interleukin-6	CRP	1
interleukin-6	Disease	1
interleukin-6	Hypertensive disease	1
LOC107984137	Periodontitis	1
Low-Density Lipoproteins	High Density Lipoproteins	1
MTND1P5	Periodontitis	1
Nitrous Oxide	vitamin B12	1
Non-alcoholic Fatty Liver Disease	Fatty Liver	1
Pharmaceutical Preparations	Atherosclerosis	1
Pharmaceutical Preparations	Ischemic stroke	1
SIGLEC5	Periodontitis	1
testosterone	Obesity	1
testosterone	Vitamin D	1
Vitamin D Deficiency	Cardiovascular Diseases	1
Vitamin D	Adiponectin	1
Vitamin D	Alzheimer's Disease	1
Vitamin D	Asthma	1
Vitamin D	cardiovascular risk factor	1
Vitamin D	Genes	1
Age related macular degeneration	Blindness	1
Angiotensin-Converting Enzyme Inhibitors	Angiotensin Receptor	1
cardiovascular risk factor	Hypertensive disease	1
cardiovascular risk factor	Obesity	1
Disease	Obesity	1
Glycosylated hemoglobin A	Diabetes Mellitus, Non-Insulin-Dependent	1
Hydroxymethylglutaryl-CoA Reductase Inhibitor:	COG2	1
IL6	C-reactive protein	1
Metabolic syndrome	Diabetes	1
Myocardial Infarction	Heart failure	1
Non-alcoholic Fatty Liver Disease	Diabetes Mellitus, Non-Insulin-Dependent	1
Obesity	Diabetes	1
Obesity	Hypertensive disease	1
Pharmaceutical Preparations	Coronary Arteriosclerosis	1
Vitamin D	Cardiovascular Diseases	1
Vitamin D	Diabetes Mellitus, Non-Insulin-Dependent	1
C-reactive protein	Obesity	1
Cardiovascular Diseases	Diabetes Mellitus, Non-Insulin-Dependent	1
Diabetes	Obesity	1
EEF1A2	COG2	1

For peer review only

Hydroxymethylglutaryl-CoA Reductase Inhibitor:	Dyslipidemias	1
Hyperandrogenism	Polycystic Ovary Syndrome	1
Hypertensive disease	Obesity	1
IGHE	Disease	1
Metabolic syndrome	Obesity	1
Obesity	Chronic disease	1
Obesity	Disease	1
Pharmaceutical Preparations	Cerebrovascular accident	1
STN gene	COG2	1
vitamin B12	homocysteine	1
vitamin E	Disease	1
folate	homocysteine	1
mullerian-inhibiting hormone	Polycystic Ovary Syndrome	1
Obesity	Cardiovascular Diseases	1
Pharmaceutical Preparations	Alzheimer's Disease	1
tocilizumab	Rheumatoid Arthritis	1
cardiovascular risk factor	Diabetes Mellitus, Non-Insulin-Dependent	1
homocysteine	Cardiovascular Diseases	1
interleukin-6	C-reactive protein	1
metformin	Diabetes	1
Hypertensive disease	Cardiovascular Diseases	1
Pharmaceutical Preparations	Asthma	1

For peer review only

RCT
1
1
4
1
1
1
1
2
2
4
5
40
1
1
1
1
1
1
1
1
2
2
3
4
8
9
13
1
1
1
1
1
1
1
1
1
1
1
1
1
1
1
1
1
1
1
1
1
1
1
1

For peer review only

1
1
1
1
1
1
1
1
1
1
1
1
1
1
1
1
1
1
1
1
1
1
1
1
1
1
1
1
1
1
1
1
2
2
2
2
2
2
2
2
2
2
2
2
2
2
2
2
3
3
3
3

For peer review only

3
3
3
3
3
3
3
3
3
3
3
4
4
4
4
4
5
6
6
6
15
18

For peer review only

subject	MR	RCT
Pharmaceutical Preparations	13	50
Lipoproteins	12	2
Obesity	11	21
Vitamin D	11	13
Diabetes Mellitus, Non-Insulin-Dependen	8	15
Insulin	7	44
Disease	6	7
C-reactive protein	5	5
homocysteine	4	9
Hydroxymethylglutaryl-CoA Reductase In	4	6
testosterone	4	3
interleukin-6	3	9
Cardiovascular Diseases	3	5
vitamin B12	3	4
ethanol	3	3
High Density Lipoproteins	3	1
Hypertensive disease	2	17
cardiovascular risk factor	2	10
Metabolic syndrome	2	5
Non-alcoholic Fatty Liver Disease	2	3
Amino Acids, Branched-Chain	2	2
Atherosclerosis	2	2
Genes	2	2
Adiponectin	2	1
Asthma	2	1
Diabetic Nephropathy	2	1
LPA	2	1
metformin	1	6
folate	1	4
mullerian-inhibiting hormone	1	4
tocilizumab	1	4
Diabetes	1	3
EEF1A2	1	3
Hyperandrogenism	1	3
IGHE	1	3
STN gene	1	3
vitamin E	1	3
Age related macular degeneration	1	2
Angiotensin-Converting Enzyme Inhibitor	1	2
Glycosylated hemoglobin A	1	2
IL6	1	2
Myocardial Infarction	1	2
Alanine Transaminase	1	1
Anti-Inflammatory Agents	1	1
Anticoagulants	1	1
APOE	1	1
Apolipoprotein A-I	1	1
Apolipoprotein E	1	1
ATP Citrate (pro-S)-Lyase	1	1
beta carotene	1	1
Body mass index	1	1

For peer review only

Cerebrovascular accident	1	1
coenzyme Q10	1	1
Complement System Proteins	1	1
Coronary heart disease	1	1
Crohn Disease	1	1
DEFA1A3	1	1
Enzymes	1	1
Free thyroxin	1	1
Hyperglycemia	1	1
Hyperlipidemia	1	1
Inflammatory Bowel Diseases	1	1
LOC107984137	1	1
Low-Density Lipoproteins	1	1
MTND1P5	1	1
Nitrous Oxide	1	1
SIGLEC5	1	1
Vitamin D Deficiency	1	1

For peer review only

object	MR	RCT
Diabetes Mellitus, Non-Insulin-Dependent	23	62
Disease	20	30
Cardiovascular Diseases	16	41
Obesity	13	20
Coronary heart disease	9	5
Diabetes	6	29
Alzheimer's Disease	5	8
Coronary Arteriosclerosis	4	3
Asthma	3	23
Cerebrovascular accident	3	5
Hypertensive disease	3	5
Atherosclerosis	3	3
Vitamin D	3	3
cardiovascular risk factor	3	2
Polycystic Ovary Syndrome	2	7
COG2	2	5
Dyslipidemias	2	4
Vitamin D Deficiency	2	3
Body mass index	2	1
Diabetes Mellitus, Insulin-Dependent	2	1
homocysteine	1	7
C-reactive protein	1	6
Rheumatoid Arthritis	1	4
Chronic disease	1	3
Angiotensin Receptor	1	2
Blindness	1	2
Heart failure	1	2
Adiponectin	1	1
Alkaline Phosphatase	1	1
Crohn Disease	1	1
CRP	1	1
Fatty Liver	1	1
Genes	1	1
High Density Lipoproteins	1	1
Insulin	1	1
interleukin-6	1	1
Ischemic stroke	1	1
Liver diseases	1	1
Low-Density Lipoproteins	1	1
Myocardial Infarction	1	1
Myocardial Ischemia	1	1
Periodontitis	1	1
thyrotropin	1	1
vitamin B12	1	1

For peer review only

BMJ Open

Systematic comparison of Mendelian randomization studies and randomized controlled trials using electronic databases

Journal:	*BMJ Open*
Manuscript ID	bmjopen-2023-072087.R1
Article Type:	Original research
Date Submitted by the Author:	06-Jul-2023
Complete List of Authors:	Sobczyk, Maria; MRC Integrative Epidemiology Unit,<br>Zheng, Jie; MRC Integrative Epidemiology Unit<br>Davey Smith, George; MRC Integrative Epidemiology Unit<br>Gaunt, Tom; MRC Integrative Epidemiology Unit
**Primary Subject Heading**:	Epidemiology
Secondary Subject Heading:	Health informatics, Genetics and genomics
Keywords:	EPIDEMIOLOGIC STUDIES, EPIDEMIOLOGY, Clinical trials < THERAPEUTICS

SCHOLARONE™
Manuscripts

I, the Submitting Author has the right to grant and does grant on behalf of all authors of the Work (as defined in the below author licence), an exclusive licence and/or a non-exclusive licence for contributions from authors who are: i) UK Crown employees; ii) where BMJ has agreed a CC-BY licence shall apply, and/or iii) in accordance with the terms applicable for US Federal Government officers or employees acting as part of their official duties; on a worldwide, perpetual, irrevocable, royalty-free basis to BMJ Publishing Group Ltd ("BMJ") its licensees and where the relevant Journal is co-owned by BMJ to the co-owners of the Journal, to publish the Work in this journal and any other BMJ products and to exploit all rights, as set out in our licence.

The Submitting Author accepts and understands that any supply made under these terms is made by BMJ to the Submitting Author unless you are acting as an employee on behalf of your employer or a postgraduate student of an affiliated institution which is paying any applicable article publishing charge ("APC") for Open Access articles. Where the Submitting Author wishes to make the Work available on an Open Access basis (and intends to pay the relevant APC), the terms of reuse of such Open Access shall be governed by a Creative Commons licence – details of these licences and which Creative Commons licence will apply to this Work are set out in our licence referred to above.

Other than as permitted in any relevant BMJ Author's Self Archiving Policies, I confirm this Work has not been accepted for publication elsewhere, is not being considered for publication elsewhere and does not duplicate material already published. I confirm all authors consent to publication of this Work and authorise the granting of this licence.

For peer review only

Systematic comparison of Mendelian randomization studies and randomized controlled trials using electronic databases

Maria K. Sobczyk[1]*, Jie Zheng[1,2,3], George Davey Smith[1], Tom R. Gaunt[1]

*To whom correspondence should be addressed.

1) MRC Integrative Epidemiology Unit, Bristol Medical School, University of Bristol, Bristol BS8 2BN, UK

2) Department of Endocrine and Metabolic Diseases, Shanghai Institute of Endocrine and Metabolic Diseases, Ruijin Hospital, Shanghai Jiao Tong University School of Medicine, Shanghai, China

3) Shanghai National Clinical Research Center for Metabolic Diseases, Key Laboratory for Endocrine and Metabolic Diseases of the National Health Commission of the PR China, Shanghai Key Laboratory for Endocrine Tumor, State Key Laboratory of Medical Genomics, Ruijin Hospital, Shanghai Jiao Tong University School of Medicine, Shanghai, China

Corresponding author: Maria K. Sobczyk (maria.sobczyk@bristol.ac.uk)

Keywords:

Mendelian randomization

Randomized Controlled Trials

Data mining

Literature mining

For peer review only

Abstract

Objective: To scope the potential for (semi-)-automated triangulation of Mendelian Randomization (MR) and randomized controlled trials (RCT) evidence since the two methods have distinct assumptions that make comparisons between their results invaluable.

Methods: We mined ClinicalTrials.Gov, PubMed and EpigraphDB databases and carried out a series of 26 manual literature comparisons among 54 MR and 77 RCT publications.

Results: We found that only 13% of completed RCTs identified in ClinicalTrials.Gov submitted their results to the database. Similarly low coverage was revealed for Semantic Medline (SemMedDB) semantic triples derived from MR and RCT publications –36% and 12%, respectively. Among intervention types that can be mimicked by MR, only trials of pharmaceutical interventions could be automatically matched to MR results due to insufficient annotation with MeSH ontology. A manual survey of the literature highlighted the potential for triangulation across a number of exposure/outcome pairs if these challenges can be addressed.

Conclusions: We conclude that careful triangulation of MR with RCT evidence should involve consideration of similarity of phenotypes across study designs, intervention intensity and duration, study population demography and health status, comparator group, intervention goal and quality of evidence.

For peer review only - http://bmjopen.bmj.com/site/about/guidelines.xhtml

STRENGTHS AND LIMITATIONS OF THIS STUDY

- Mendelian randomisation (MR) has become a popular method in causal inference in genetic epidemiology, and while often used as proxy for to randomized clinical trials (RCT), little is known about scope for automatic comparison between MR and RCT results.
- Previous research has established conceptual similarities and differences between MR and RCT methodology, however, without focus on applied cases.
- The study found that a low percentage of completed RCTs were submitted to ClinicalTrials.gov and that a similarly low coverage was found for MR and RCT publications in Semantic Medline. Only trials of pharmaceutical interventions could be automatically matched to MR results due to insufficient annotation with MeSH ontology among other interventions.
- Following manual extraction of MR and RCT literature, we assessed result concordance across the two methods and discussed multiple possible reasons for discrepancies.
- Sparsity of data in electronic databases hinders the ability to automatically compare results of MR and RCT studies. In the absence of retrospective manual extraction of MR and RCT results from publications, more research effort needs to be spend developing machine-learning approaches to aid systematic comparisons. Our study helps identify study design features which need to be captured by such methods.

For peer review only

Background

Randomized controlled trials (RCTs) are deemed the “gold standard” in evaluating the efficacy of interventions and guiding practice in clinical research, with well-established methodology[1]. In RCTs, a selection of individuals intended to represent the target population is randomly assigned to a treatment or control group, allowing estimation of the intervention’s effectiveness in the absence of confounding variables and reverse causality that are present in observational studies. In the past two decades an approach to causal inference using natural genetic variation, known as Mendelian Randomization (MR) – usually implemented as an instrumental variable (IV) analyses – has gained popularity[2,3]. This approach has been referred to as “nature’s randomized trials”[4], and is based on the randomization from parents to offspring of genetic variants encapsulated in Mendel’s laws of segregation and independent assortment[2,5]. At a population level the randomization is approximate, but still allows genetic variants that are robustly associated with the measured exposure to be used to estimate the unbiased causal effect of an exposure (generally acting across life) on health outcomes, as long as certain assumptions, discussed in detail elsewhere[2,3,6], are met.

Despite drawing on observational data, the MR approach broadly aligns with that of a randomized controlled trial, where the goal is to estimate the causal effect of an intervention on the given endpoint based on groups (arms) which do not differ with respect to confounding variables (Figure 1). However, since in MR randomization takes place at conception, the time lag to the start of outcome recoding is longer[7] compared to RCTs, where median duration of phase 3 trials is 40 months[8]. Similarly to RCTs, most MR

For peer review only

analyses should be free of confounding and reverse causation bias due to variants being allocated randomly before birth and outcome condition onset.

Previous research has shown examples of evidence triangulation where MR results predicted the overall RCT results based on totally orthogonal data with an unrelated set of systematic errors and biases[9]. For instance, MR demonstrated the lack of effect of genetically-predicted concentrations of HDL-C on cardiovascular events[10–12] as well as selenium in prostate cancer prevention trials[13,14]. On the other hand, MR showed the beneficial effect of lifelong endogenous low LDL-C levels[15], HMG-CoA reductase inhibition (statin drug target) and PCSK9 inhibition on cardiovascular disease[15,16], while predicting also the increased risk of type 2 diabetes (T2D) as a side-effect of statin usage. However, three independent MR studies were at odds with later RCTs by predicting increased risk of T2D also as a side-effect of PCK9 inhibition[17].

There are several possible explanations for apparent or real discordance in the results of RCT and MR studies. These range from different durations, magnitude and time-varying nature of the exposure, origin of the study populations, and natural genetic variation imperfectly mimicking the molecular action of the drug, some of which we explore. The direct comparison of MR and RCT findings is facilitated by the use of a precisely-defined estimand[18], for example, the effect on incident coronary heart disease risk of lowering LDL cholesterol by 1 mmol/l for 5 years. Whilst RCTs will estimate something close to this, and be scalable to it, with MR studies the exposure difference associated with the genetic instruments will often exist from birth (or before) and may change in magnitude over time[19]. This is discussed further in the Supplementary Box.

For peer review only - http://bmjopen.bmj.com/site/about/guidelines.xhtml

Whilst RCTs can provide the highest-quality evidence, they may have limitations. They are often expensive to carry out, can be of small size and lack external validity[20], have short follow-up and typically take place after disease onset[21,22]. As in other study types, RCT results may be flawed due to poor design and execution, e.g. imperfect randomization, unblinding and differential loss to follow-up between study arms.

Unlike RCTs, MR studies are inexpensive and quick to perform when suitable genetic instruments are available. Therefore, they can potentially prioritize intervention-condition pairs to assess in RCTs. Moreover, it has been proposed that MR also guide the design of RCTs, improving eligibility criteria to prioritise groups most likely to benefit, suggesting diseases for composite endpoint construction, and alerting to potential side-effects[1,23]. Since MR analyses suffer a different set of biases than RCTs, MR evidence can be used to complement RCTs and other study designs in the triangulation framework to guide therapeutic development and clinical practice[24–26]. Finally, the extensive use of existing observational data for MR enables intervention targets to be evaluated in a wider range of sub-populations than is feasible for RCTs (improving generalizability), and allows comparisons to be made that might be unethical in experimental studies, for example when there is strong evidence in favour of a particular treatment.

The goal of this research is to survey the extent of concordance between MR and RCT studies to date and identify possible factors for disparities in the direction of effect, which limit the ability to extrapolate from MR results to RCTs and increase the complexity of the triangulation process. In this study, we aimed to carry out a systematic analysis of MR and RCT results using automated mining of data in the public domain, including the ClinicalTrials.Gov[27], EpigraphDB[28] and PubMed databases. We evaluate the

For peer review only - http://bmjopen.bmj.com/site/about/guidelines.xhtml

comprehensiveness and scope of the data available and potential for comparative analyses between MR and RCTs. We then go on to develop a series of case-studies looking in detail at MR and RCT comparisons across 26 exposure-outcome pairs. Throughout, we use the term “intervention” as synonymous with “exposure” and “condition” as synonymous with “outcome”.

Methods

ClinicalTrials.Gov *Data sources*

All ClinicalTrials.Gov study data is available for download as PostgreSQL database from The Database for Aggregate Analysis of ClinicalTrials.gov (AACT)[29] released by The Clinical Trials Transformation Initiative[30]. We downloaded its static release from 1st July 2023. Processing of the database files was carried out using custom Python and R scripts.

ClinicalTrials.Gov *Data filtering*

We filtered the ClinicalTrials.Gov studies using a number of criteria to identify RCTs with submitted results allowing direct comparison with MR studies. These are depicted in Figure 2a and provided in detail in Supplementary Note.

EpigraphDB *queries*

EpigraphDB[28] was used to collect information about confirmed drug-target associations which were initially sourced from the Open Targets Platform[31] and verified in DrugBank[32]. EpigraphDB was then used to retrieve eQTL and pQTL MR results previously described in Zheng et al. (2020)[33]. We also used EpigraphDB[28] to source SemMedDB[34] ver. 1.8 semantic triples associated with select MR and RCT publications identified by

For peer review only

PubMed. SemMedDB triples in EpigraphDB are prefiltered for annotation of epidemiological studies as described previously[35].

PubMed *Data harvesting*

We searched PubMed for all RCT and MR studies published before 2023 on 1st July 2023. For RCTs, we searched titles and abstracts for keywords: “randomized controlled trial” or “RCT” and we used PubMed’s in-built *Randomized Controlled Trial* label filter to obtain more specific hits, reducing the number of hits from 129,077 to 74,559. In order to retrieve potential MR studies, we used the keywords: “mendelian randomization” or “mendelian randomisation”. We also considered using a MeSH label “Mendelian Randomization Analysis” but it returned an unrealistically low number of hits (3,174), a consequence of manual indexing.

Literature searches

We used Semantic Scholar and Google Scholar to survey MR and RCT literature indexed before 1st July 2023. We queried the databases with the following search terms: “[exposure] [condition] Mendelian Randomization” and “[exposure] [condition] Randomized Controlled Trial”. The articles were initially screened by title and abstract. We considered original research MR, RCT studies as well as meta-analyses. We included 26 intervention-outcome pairs to represent a wide array of behavioural and nutritional interventions with a diverse set of common disease (cardiometabolic, neuropsychiatric, cancer, dermatological) and disease biomarker outcomes, based on our expert knowledge of the field. Our chosen exposures correspond to the top four modifiable risk factors accounting for 39% of deaths in the USA[36]: high alcohol intake, high body mass index (BMI), lack of exercise and smoking. In addition, since potentially preventative effects of nutritional factors are controversial and

For peer review only - http://bmjopen.bmj.com/site/about/guidelines.xhtml

notoriously difficult to evaluate using non-randomized study designs[37], we also included vitamins D and E as well as coffee as an intervention. We acknowledge that this choice is somewhat subjective but we believe it to be illustrative of the current MR and RCT literature.

MR and RCT studies were compared across: population characteristics (sex, ethnicity, age, health status), comparator group, goal of intervention (prevention or treatment/slowing progression), direction of effect, length of follow-up, main test statistic in the study and its impact as judged by citation number.

Patient and Public Involvement

Patients or the public were not involved in the design, or conduct, or reporting, or dissemination plans of our research.

Results

ClinicalTrials.Gov data overview

In total, we found 457,254 individual studies were registered with a unique ClinicalTrials.gov identifier. We filtered them using a number of steps to identify RCTs and facilitate comparison with MR (Figure 2a). In our analysis, we identified 218,506 RCT studies (48% of the total). To allow semi-automated comparison with MR studies, we focussed on the study subset which submitted their statistical analysis results to the database (referred to as the **main dataset,** Supplementary Dataset 1). However, we found that only 3.4% of studies – 15,752 met this criterion, along with including background information on the trial. To expand that number, we also considered an additional 28,538 RCT studies which did not

For peer review only - http://bmjopen.bmj.com/site/about/guidelines.xhtml

publish their results in ClinicalTrials.gov but instead linked to a peer-reviewed publication (referred to as the **literature dataset,** Supplementary Dataset 2).

The majority of RCTs in the main dataset followed parallel assignment of participants to treatment (Figure S1a), most were designed for treatment (n= 12,336, Figure S1b), rather than prevention (n=1,613) and the vast majority of them had been completed (Figure S1c). More trials were observed to be in phase 3 than 4 (Figure S1d), most trials included both males and females (Figure S1e), and a great majority had 2 arms (Figure S1f). The median number of primary outcomes was 1 (Figure S2a), with a median of 6 secondary outcomes (Figure S2b). Over half of studies report at least 1 result with *p*-value less than 0.05 (Figure S2c). Comparison with features of all RCTs in the database showed that our selection was broadly representative (Supplementary Dataset 3), although our dataset was enriched for completed and late-phase trials.

Suitability of MeSH annotation

In order to attempt automated matching of RCTs and MRs involving similar interventions and outcomes for RCTs and MR, we needed to first establish the quality of annotation of RCTs with MeSH (Medical Subject Headings) in ClinicalTrials.Gov. The most common intervention was Drug (Table S1). Since we were only interested in the intervention types which can be instrumented by MR, we also focussed on the 5th and 7th most popular types of interventions: Behavioral and Dietary Supplement. We found that MeSH intervention annotations were missing for only 19% and 16% of Drug interventions in the Main and Literature datasets, accordingly (Table 1). However, the overwhelming majority of RCTs in the Behavioral and Dietary Supplement category did not contain a MeSH intervention term. Due to well standardised disease taxonomy, a much lower level of missing data was found

For peer review only - http://bmjopen.bmj.com/site/about/guidelines.xhtml

for MeSH condition terms. This allowed us to proceed with automated analysis of Drug RCT data, however, for Behavioral and Dietary Supplement we were only able to do a manual screening for RCTs with corresponding MR studies.

For peer review only

For peer review only - http://bmjopen.bmj.com/site/about/guidelines.xhtml

Table 1. Completeness of MeSH term annotation amongst the chosen intervention types in the *Main* (RCT results available in ClinicalTrials.Gov) and *Literature* (RCT results unavailable in ClinicalTrials.Gov but study linked to a publication with results) datasets.

Dataset	Intervention type	Total	intervention MeSH missing	% missing	condition MeSH missing	% missing
Main	Drug	11,537	2,212	19.2%	992	8.6%
Literature	Drug	10,927	1,773	16.2%	1031	9.4%
Main	Behavioral	1,239	1,075	86.8%	242	19.5%
Literature	Behavioral	3,017	2,815	93.3%	620	20.5%
Main	Dietary Supplement	242	116	47.9%	31	12.8%
Literature	Dietary Supplement	830	581	70.0%	164	19.8%

For peer review only

Pharmaceutical interventions in RCTs and MR

Genetic instrumental variables in MR can be used as proxies for pharmaceutical interventions in RCTs. Protein (pQTL) or expression (eQTL) quantitative trait loci (QTL), i.e. variants associated with expression of protein drug targets are used to directly proxy the action of a drug. Here we use the biggest MR dataset for drug target protein-disease associations, examined in whole blood, from Zheng et al. (2020)[33]. We focussed on *cis*-acting instruments as a more specific marker for drug efficacy as *trans*- instruments are more likely to be pleiotropic, potentially leading to spurious results[23].

We matched the drug target proteins in Zheng et al. (2020) with drug-gene associations sourced from EpigraphDB[28] (**Figure 2b**). This allowed us to merge the Zheng et al. (2020) dataset with the main and literature RCT dataset via the drug listed in EpigraphDB and MeSH drug intervention term, accordingly. For the outcome, we were then able to match RCTs and MR manually due to the reasonably low number of hits. The results, displayed in Table 2 show overlap of the RCT and MR datasets. We found 4 drugs: evolocumab/alirocumab, ustekinumab and mipomersen that share support from both MR and RCT studies. evolocumab/alirocumab and mipomersen inhibit key players (PCSK9 and apoB) in lipid transport helping to lower plasma LDL-C levels[38]. The Zheng et al. (2020) MR study showed a negative effect of reduced PCSK9 levels on high cholesterol in the UK Biobank, while in 25 RCT studies drug-induced abrogation of PCSK9 activity led to positive outcomes in the treatment of hyperlipidemia, hypercholesterolemia and dyslipidemias in general. Similarly, reduced expression/activity of apoB in MR and 6 RCT studies resulted in genetically predicted lower levels of LDL cholesterol and total cholesterol in the UK Biobank as well as improved outcomes in the treatment of dyslipidemias, respectively. The third

For peer review only

Table 2. Drug target-disease matches supported by evidence from MR (blood pQTL instruments, Zheng et al. 2020) and RCT studies (*Main dataset* from ClinicalTrials.Gov).

MR Exposure	RCT Drug Intervention	MR Outcomes	RCT Conditions	Matching trials	Concordant direction of effect?	xQTL
PCSK9	EVOLOCUMAB, ALIROCUMAB	Non-cancer illness code self-reported: high cholesterol || id:UKB-a:108	Hyperlipidemia, Dyslipidemia, Hypercholesterolemia, Mixed Dyslipidemia	25	Yes	pQTL
APOB	MIPOMERSEN	LDL cholesterol || id:300, HDL cholesterol || id:299, Triglycerides || id:302, Non-cancer illness code self-reported: high cholesterol || id:UKB-a:108, Total cholesterol || id:301,	Hyperlipidemia, Dyslipidemia, Hypercholesterolemia, Mixed Dyslipidemia	6	Yes	pQTL
IL12B	USTEKINUMAB	Non-cancer illness code self-reported: psoriasis || id:UKB-a:100; Ulcerative colitis || id:970; Crohn's disease || id:12; Inflammatory bowel disease || id:294	Psoriasis, Psoriatic Arthritis, Crohn's Disease, Colitis, Inflammatory Bowel Disease	21	Yes	pQTL

For peer review only

example of a good match between RCT and MR studies concerns inhibition of the p40 subunit of interleukin 12 and 23 (IL12B)[39]. Both MR and 21 RCTs show benefit of inhibition of p40 on immune-mediated disease: psoriasis and inflammatory bowel disease.

In general, pQTL MR-based prediction of drug target-condition pairs offered good recall when compared with the pairs in the Open Targets Platform for the proteins with MR evidence. The only drug target indications missing included conditions not analysed in the MR study, such as CD33 protein being the drug target for treatment of leukemia, with the exception of ACHE (acetylocholinoesterase) whose inhibitors (galantamine, donepezil, rivastigmine) are used for treatment of cognitive decline in Alzheimer's disease[40].

We also compared the RCT dataset with Zheng et al. blood transcript expression (eQTL)-derived MR analysis (available in EpigraphDB: https://epigraphdb.org/xqtl, Figure S3). In total, we identified 15 drug target-disease matches in the eQTL dataset (Table S2), although unlike in the pQTL matches, the direction of effect in MR was incorrect in 8 cases. Nevertheless, the eQTL MR results agreed with some well-known drug effects: HDL-C and LDL-C lowering action of CETP and HMGCR inhibitors[41], respectively, and blood pressure-lowering action of ACE inhibitors[42].

PubMed-sourced MR and RCT studies

In addition to searching through the ClincalTrials.Gov database, we also queried PubMed for RCT and MR publications. In total, we found 5,135 MR studies published since mid-2000s and 73,306 RCTs published since 1970 until 2022 (Figure 3).

Semantic analysis with SemMedDB

For peer review only

We subsequently wanted to establish the thematic overlap between MR and RCT studies using an alternative method involving semantic analysis. SemMedDB[34] provides a vast repository of semantic predications (subject-predicate-object triple, e.g. LDL-C causes ischemic heart disease). We linked the MR and RCT publications identified by our PubMed search to their corresponding SemMed triples in EpigraphDB using PubMed ID (Supplementary Dataset 4). Overall, only 12% and 36% of RCT and MR papers, respectively, had a semantic triple associated with them (Figure 3). When ignoring the predicate, and focusing only on the subject and object, we found a total of 15,113 unique exposure-outcome pairs (Figure S4), discussed in detail in the Supplementary Note. However, only 221 of these were found to be shared across MR and RCT studies.

We then investigated the 221 matching subject-object pairs between MR and RCT studies (Table S3), as well as individual top counts among subjects (Table S4) and objects (Table S5). T2D, insulin and obesity were found among the top shared risk factors, along with lipids and vitamin D. Top outcomes included T2D, cardiovascular disease, COVID-19, and Alzheimer's disease.

Case studies of matching MR and RCTs

Since our semi-automatic mining of MR and RCT literature brought limited results for behavioural and nutritional interventions, we selected 26 intervention-outcome case-studies by manual mining of the literature representing common lifestyle risk factors, dietary and behavioural exposures, paired with common cardiovascular, glycemic, neuropsychiatric, musculoskeletal, autoimmune and cancer outcome phenotypes. In total, we surveyed 54 MR and 77 randomized controlled trial publications (RCTs and meta-analysis of RCTs, Supplementary Dataset 5, Figure 4) which were systematically compared across

For peer review only - http://bmjopen.bmj.com/site/about/guidelines.xhtml

several criteria shown in sample Table S6, and encompass those in the popular PICO (Population, Intervention, Comparison, and Outcome) framework[43].

There, we compare an MR study and two RCT meta-analyses on the effect of vitamin D supplementation in multiple sclerosis (MS)[44–46]. Whilst the RCTs looked at potential therapeutic effect of vitamin D in diagnosed MS patients over 6 months-2 years: measured disability (EDSS score) and recorded relapses as outcomes, the MR study took place in the general population and measured the causal effect of genetically predicted lifetime circulating vitamin D concentrations on prevention of MS. The conclusions of MR and RCT studies did not align well, with MR analyses providing evidence for reduced risk of MS conferred by higher vitamin D levels, but no significant therapeutic effect of vitamin D in existing MS was found in the 5 small meta-analyzed trials. Differences which may impact on the ability for MR to complement RCT studies are summarised in Table 3 and discussed below based on this series of case-studies.

For peer review only

For peer review only - http://bmjopen.bmj.com/site/about/guidelines.xhtml

Table 3. Overview of discussed criteria for assessment of alignment of MR and RCT study features.

Match criterion	Issues to consider	Example
Exposure (intervention)	similarity between analyzed exposures	different types of exercise
	intervention intensity	vitamin D dosage
Intervention goal	prevention or treatment	schizophrenia onset or treatment
Outcome (condition)	single or composite outcome	single or composite cardiovascular outcomes
	binary versus categorical outcome	depression or rating on depression assesment scale, such as Hamilton Depression Rating Scale
	similarity between analyzed outcomes	different measures of adiposity
Source population	demographics	young adults or elderly
	health status	diabetic or healthy
Comparator group	exposure-naïve or previously exposed	ex-smoker or never-smoker
	active intervention or placebo	statin as comparator or placebo
Duration of intervention/follow-up	length of intensive intervention and follow-up	short duration of intervention (< 6 months) or long duration and follow-up (>3 years) in RCT and MR
	not uniform intervention intensity or duration	weekly counselling during the initial phase of the trial or throughout

For peer review only

1) Exposure/Intervention

We found that overlapping MR and RCT interventions are often not perfectly identical which may impact on the estimated direction of effect. For example, MR exercise exposures are based on genetic variants associated with self-reported physical activity (moderate-to-vigorous and vigorous)[47,48] in studies assessing the effect on both lipids and bone mineral density (BMD). However, the corresponding RCTs used particular types of exercise, such as walking[49], aerobic exercise[50,51], progressive resistance training[52] and maximal strength training[53] as interventions. While a MR study[48] and two trials[53,54] showed concordant (Figure 4), positive effect of exercise on BMD, we found that the effect of exercise on lipids did not match between MR and RCTs, with MR study[47] reporting null effect and trials generally finding positive effects on HDL-C concentration and negative on LDL-C, total cholesterol and triglycerides blood levels[50–52,55].

Furthermore, intensity of intervention can affect the comparative value of MR and RCT study conclusions. The MR study of vitamin D levels on bone fractures[56] was only able to assess linear effects of the normal range of circulating 25-hydroxyvitamin D concentrations. Consequently, the positive effect of high-concentration vitamin D (≥700 IU daily) on bone fractures in the elderly seen in RCTs[57–59] may not have been accessible in the MR study.

2) Intervention goal

The intervention goal between MR and RCT studies can match (both prevention or treatment) or be misaligned which can potentially impact the ultimate conclusions of the study. We found the latter to be the case for the effect of exercise on schizophrenia. Two MR studies found a null preventative effect of exercise on schizophrenia[60,61], while 3

For peer review only - http://bmjopen.bmj.com/site/about/guidelines.xhtml

For peer review only

meta-analyses of RCTs found a consistent effect of a variety of exercise types on improving total and negative symptoms of schizophrenia[62–64] (Figure 4).

3) Outcome

The short duration of RCTs mean some outcomes (e.g. myocardial infarction) do not accumulate enough events to detect a significant effect, therefore composite measures grouping related diseases are often used. When comparing the effect of systolic blood pressure (SBP) on cardiovascular disease (CVD) outcomes, we found matching conclusions with elevated SBP increasing the risk of CVD both in RCT[65–67] and MR[68–70] studies (Figure 4), with MR studies using both single disease outcomes and a composite outcome. However, MR studies analysing the impact of BMI on cardiovascular disease found reduced adiposity led to reduction in arterial hypertension, CVD and stroke[71,72], which contrasted with the results of one of the biggest RCTs to date. The Look AHEAD RCT in older type 2 diabetics found no preventative effect of weight loss on a composite outcome relating to mortality from cardiovascular causes, non-fatal myocardial infarction, non-fatal stroke or hospitalization for angina[73].

Secondly, RCT outcomes, are often on a quantitative scale measuring symptom strength according to established metrics, (e.g. depressive symptoms on Hamilton Depression Rating Scale[74]). However, the best disease GWAS used to identify MR instruments often represent binary disease outcomes, which could potentially lead to differential conclusions due to reduced power to detect subtler therapeutic effects. While exercise is causally associated with reduced depression and depressive symptoms both in MR[75,76] and RCTs[62,74,77–80], the differences in outcome phenotypes could potentially contribute to

For peer review only - http://bmjopen.bmj.com/site/about/guidelines.xhtml

null MR results[81,82] and positive effect of vitamin D on attenuating eczema symptoms in RCTs[83–86].

4) Source population

MR studies are likely to draw from a wider demographic than RCTs due to use of biobanks and GWAS consortia, while RCTs focus on high risk groups[23]. For example, while in the MR study conducted in general population, there was no strong significant effect of exercise on glycemic markers: HbA1c, fasting glucose and HOMA-IR[87], a significant reduction was found in the meta-analysis of 32 RCTs involving patients with T2D[88].

On the other hand, 5 Mendelian randomization[89–93] studies along with 3 large RCTs[94–96] consistently provide evidence that weight loss is causally associated with reduced risk of T2D (Figure 4), despite MR including the general population and RCTs focussing on at-risk individuals with impaired glucose tolerance.

As another example of possible demographics-driven differences in trial and MR results, MR studies on the relationship between vitamin D levels and atopic dermatitis were conducted in the general population[81,82], while RCTs were conducted separately in children in Mongolia[83] and Boston, USA[84] with winter atopic dermatitis and in adolescent and adult Iranians[85,86].

5) Comparator group

Firstly, due to ethical considerations, trials of harmful behaviours such as alcohol drinking and smoking focus on cessation or reduction in existing users, and do not include never-smokers or never-drinkers as controls, unlike MR studies, which can potentially lead to differences in effect[23]. Nevertheless, the two outcomes analysed here: hypertension for

For peer review only - http://bmjopen.bmj.com/site/about/guidelines.xhtml

alcohol intake and lipids for smoking showed generally congruent results across study types (Supplementary Dataset 5).

Secondly, where it would be unethical to withhold already available efficacious treatments, trials will often include another active intervention in the comparator group[23], for example statins in the trials of effect of PCSK9 inhibitors[97,98] on LDL-C and cardiovascular events. Such RCT design can be mimicked by factorial MR estimating the interaction of multiple exposures, as shown in matching results of the equivalent MR study[16].

6) Duration of intervention

While the magnitude of effect seen in trials with long (> 3 years: weight loss to treat hypertension[99,100]/type 2 diabetes[94–96], blood pressure reduction to lower cardiovascular disease risk[65–67]) and short (< 6 months: alcohol intake reduction to lower blood pressure[101–104], exercise to benefit bone mineral density[53]/depression[62,74,78–80,105]) intervention may vary, we find both can result in directional effects consistent with MR results (Figure 4), although with exceptions[73,106].

Triangulation of MR and RCT results

Combining RCT and MR results can offer complimentary evidence on the effectiveness of interventions. Powerful examples include congruence of positive effect of high BMI on hypertension across MR[107–110] and RCT[99,100] studies, high BMI on T2D risk in MR[89–93,111] and RCTs[94–96], and the null effect of vitamin D on various glycemic markers in diverse populations in MR[112], RCTs[113,114] and RCT systematic review[115].

We also found cases, where the majority of studies pointed to one direction of effect, with one MR or RCT identified as an outlier study. In these cases, having a wide array of MR and

For peer review only - http://bmjopen.bmj.com/site/about/guidelines.xhtml

RCT studies (ideally meta-analysed) can be helpful in establishing the likely true causal direction of effect. For instance, 2 MR studies[116,117], a meta-analysis of 5 RCT studies[118] and two RCTs[119,120] indicate no effect of vitamin E on prostate cancer incidence with one outlier RCT[121] showing benefit of vitamin E supplementation in older smokers. Similar contrary findings were found for 1 RCT[122] showing beneficial effect of vitamin D on preventing depressive symptoms, as opposed to null effect in 4 MR studies[123–126] and 2 RCTs[127,128].

On the other hand, MR analyses can show spurious disagreement with the rest of the evidence base. For instance, 2 MR papers[129,130] and a meta-analysis of 16 RCT[130] studies reveal no significant effect of vitamin D on blood pressure in the general population, with the exception of one MR study[131] that indicated a blood pressure-lowering effect of higher vitamin D status. Similarly, a range of study types: one MR analysis[132], one RCT[133] and a meta-analysis of 27 prospective cohorts[134] (only some of them RCTs) confirm a negative impact of smoking on HDL-C levels, bar one MR study showing no significant effect[135]. A series of RCT meta-analyses[136–138] support an effect of coffee consumption (especially unfiltered) on unfavourable blood profile, although this is likely explained by diterpenes[139,140] rather than caffeine, as the latter shows evidence of cardioprotective effects[141]. However, only the recent biggest MR study[140] to date found a significant effect of coffee consumption on LDL-C and total-C levels, unlike two previous smaller MR analyses[142,143], which found a non-significant directionally-consistent relationship.

For peer review only - http://bmjopen.bmj.com/site/about/guidelines.xhtml

Discussion

Our study highlighted that sparsity of data in the electronic databases seriously hampers the ability to automatically parse and compare results of MR and RCT studies. Released for the first time in 2000, ClinicalTrials.Gov is the most comprehensive resource for modern RCT (only <1,000 studies, out of ~218,000 analysed RCTs were started before 2000). Nonetheless, we found that only 13% of all completed RCTs submitted their results to ClinicalTrials.Gov, with median trial start date in 2012. Despite 2007 legislation requiring submission of RCT results to ClinicalTrials.gov within 1 year of completion (with exceptions)[144], only 38% of eligible trials for 2008-2012 submitted their results at any time[145] which rose to 64% for 2018-2019[146]. Furthermore, 60% of studies for failed agents are reported not to be published in peer reviewed journals[147], and in the work presented here we found MeSH annotations were missing from the majority of complex, behavioural and dietary interventions. These factors significantly hamper efforts to systematically triangulate RCT evidence with other studies.

Next, semantic triples describing conclusions of MR and RCT studies automatically extracted from literature abstracts using rule-based methods also had low coverage, with only 36% of MR and 12% of RCT studies associated with >=1 triples. Consequently, we instead decided to focus on a detailed qualitative investigation of a series of case studies to identify the issues associated with triangulating MR and RCT studies

Combining RCT and MR results can offer complimentary evidence on the effectiveness of interventions. Powerful examples include congruence of positive effect of high BMI on hypertension across MR[107–110] and RCT[99,100] studies, high BMI on T2D risk in MR[89–93,111] and RCTs[94–96], and the null effect of vitamin D on various glycemic markers in

For peer review only - http://bmjopen.bmj.com/site/about/guidelines.xhtml

diverse populations in MR[112], RCTs[113,114] and RCT systematic review[115]. We also found cases, where the majority of studies pointed to one direction of effect, with one MR or RCT identified as an outlier study. In these cases, having a wide array of MR and RCT studies (ideally meta-analysed) can be helpful in establishing the likely true causal direction of effect.

Our analysis of genetically predicted effects of perturbation of drug target protein expression on a number of conditions with trials submitted to ClinicalTrials.Gov revealed good concordance with established therapeutics for pQTLs. However, due to the limited number of proteins (n=1,002) and phenotypes (n=225, many non-diseases *per se*) in Zheng et al. (2020)[148], the comparison is necessarily very preliminary. We identify only true positive cases, as false positives and true negatives are difficult to evaluate due to sparsity of drug clinical trial results in ClinicalTrials.Gov/literature[147] and inclusion of non-disease phenotypes in MR analysis. Anecdotally, we found no MR evidence that decreased expression of PLA2G2A leads to reduced cardiovascular disease, which agrees with lack of efficacy of PLA2G2A inhibitor in clinical trials[149–151].

The mixed reliability of eQTL instruments in predicting direction of effect on the outcome could be due to a number of factors such as: less than perfect correlation between mRNA and protein levels[152], hidden pleiotropy in single instruments used in the MR analysis (directly observed for IL2RA)[153], presence of negative feedback loop involved in the drug mechanism[154], translation into protein isoforms with distinct biological effects[155] and differential cell-type specific drug effect[156].

The duration of intervention varies between RCTs and MR studies, with the former spanning no more than the duration of the trial, whilst the latter can represent durations as long as

For peer review only - http://bmjopen.bmj.com/site/about/guidelines.xhtml

the entire lifetime (although many exposures, such as alcohol intake, will be over a shorter time period)[7]. Moreover, intervention in RCTs with long follow-up is not necessarily similarly intensive throughout its duration, or may cease altogether after some time[74,99], i.e. duration of follow-up is longer than duration of intervention in order to allow accumulation of enough events and/or confirm durability of intervention effect. Examples include lifestyle interventions, such as exercise[74] or weight loss programmes[94] like the Look AHEAD trial, with median follow-up of 9.6 years, where group and individual counselling sessions took place weekly in the first 6 months and tapered off over time[73]. That is why our analysis focussed on comparing direction of effect, while ignoring magnitude of effect[157]. However, in certain cases when enough reference data is available, it is feasible to compare MR and RCT effects on the same exposure difference scale[158].

Further impediments to direct comparison between MR and RCTs include differences in outcome definition (composite[65] versus single conditions[70]). Access to rare subpopulations with existing conditions, such as cancer patients receiving specific therapy[159] which are routinely exclusively enrolled into RCTs can be difficult in MR due to the size of GWAS biobanks relative to *N* required for good power.

There are also a number of interventions and outcomes with no single phenotype which could be instrumented with GWAS variants, making MR approaches difficult, although sometimes possible with innovative MR approaches[160]. This is especially true of lifestyle interventions – such as different forms of psychological therapy, complex diet regimens[161] and fasting. Absent or limited heritability of a number of interventions and conditions, such as rehabilitation and traumatic injury makes MR approaches inaccessible.

For peer review only - http://bmjopen.bmj.com/site/about/guidelines.xhtml

The majority of MR studies track the onset rather than progression of disease due to availability of GWAS phenotypes[162] which are often a (binary) single measurement, as opposed to multiple quantitative outcomes frequently measured in RCTs[163]. For that reason, triangulation of MR of onset with RCTs whose intervention is targeting progression of disease, may or may not result in agreement, as seen in our comparison of the effect of exercise on schizophrenia onset/progression (discordant) or depression (concordant) and vitamin D effect on atopic dermatitis onset/progression (discordant).

Many MR studies may be underpowered due to large sample required in indirect estimation[164] as these studies are typically studies of convenience. This bias is less common in RCTs due to pre-registration of study design including power analysis[1], uncommon in MR[165]. Null effect in MR studies may be therefore spurious and not predictive of RCTs for that reason, as seen in two smaller MR studies[142,143] out of three[140] investigating the effect of coffee intake on blood lipids, contrasting with strong clinical trial[136–138] and biochemical evidence[139,140,166].

Furthermore, the presented literature survey used a simple heuristic of reported statistically significant evidence (p-value < 0.05 after multiple testing correction) to compare conclusions across MR and RCT studies, which has well-known limitations[167,168]. Inclusion of the full-spectrum of scaled point-estimates along with their confidence intervals will reveal a more detailed picture in triangulation of MR and RCT evidence (Supplementary Box).

Overall, we find that due to difficulty in identifying sufficient number of MR-RCT pairs matched for the same exposure and outcome, we cannot derive a numerical model to quantify reliability and importance of features of MR analysis in predicting the outcome of a

For peer review only - http://bmjopen.bmj.com/site/about/guidelines.xhtml

future RCT. However, we make several general observations regarding usefulness of triangulation[169] of RCT with MR to guide MR studies. If an RCT shows a causal relationship between an intervention and an outcome which corresponds to the one observed in MR, it can help validate the use of these genetic variants as instruments in future MR studies. Moreover, RCTs can inform MR analyses about the plausible effect sizes and so can be useful for power calculations in MR. RCTs can help identify important interactions and subgroup effects, which can further inform MR study design. For instance, if an RCT identifies that a treatment has a stronger effect in a particular subgroup of individuals (e.g., females, children), they could be analysed separately using one-sample MR.

Conclusions

Our research highlights the challenges and benefits of triangulation of MR with RCT evidence. Future efforts, outside of the scope of this work, will focus on fully quantitative approaches towards triangulation, involving magnitude of effect size and not just its presence and direction[25]. Developers of such methods will need to be mindful of discrepancies in research hypothesis, duration and intensity of exposure, outcome measures, intervention aim, underlying population characteristics, violations of test assumptions as well as statistical power of the analysis. Furthermore, automated triangulation based on electronic databases requires intensive effort towards structured capture of both MR and RCT study results and associated meta-data, as well as annotation with shared ontologies, which is still challenging using current natural language processing methods, despite constant progress[43,170,171].

For peer review only - http://bmjopen.bmj.com/site/about/guidelines.xhtml

List of abbreviations

BMD – Bone mineral density

BMI – Body mass index

CVD – Cardiovascular disease

eQTL – Expression quantitative trait loci

IV – Instrumental variable

MR – Mendelian randomization

MS – Multiple sclerosis

pQTL – Protein quantitative trait loci

SBP – Systolic blood pressure

RCT – Randomized controlled trial

T2D – Type 2 diabetes

Declarations

Ethics approval statement

This study does not involve human participants and ethical approval was not required.

Data sharing statement

All data generated or analysed during this study are included in this published article and its supplementary information files. Code used to carry out the analysis is available on GitHub: https://github.com/marynias/mr-rct.

For peer review only - http://bmjopen.bmj.com/site/about/guidelines.xhtml

ClinicalTrials.Gov data was accessed via AACT: https://aact.ctti-clinicaltrials.org/download and analysed data subset is featured in Supplementary Datasets 1 & 2.

pQTL and eQTL MR analysis results are available via EpigraphDB: https://epigraphdb.org/xqtl

PubMed database can be accessed on https://pubmed.ncbi.nlm.nih.gov/ and analysed data subset is featured in Supplementary Dataset 4.

SemMedDB can be accessed on https://lhncbc.nlm.nih.gov/ii/tools/SemRep_SemMedDB_SKR/SemMedDB_download.html and analysed data subset is featured in Supplementary Dataset 4.

Case series of MR and RCT studies with matching exposures (interventions) and outcomes (conditions) is featured in Supplementary Dataset 5.

Supplementary Datasets 1-5 are available for download on Zenodo: https://doi.org/10.5281/zenodo.8104176

Competing interests

JZ, GDS and TRG receive funding from Biogen for unrelated research. MKS has no competing interests.

Funding information

We would like to acknowledge UK Medical Research Council [mc_uu_00011/4] which provided funding for our work carried out at the University of Bristol MRC Integrative Epidemiology Unit. Our funder played no role in the design of the study, analysis, and interpretation of data and in writing of the manuscript.

For peer review only - http://bmjopen.bmj.com/site/about/guidelines.xhtml

Contributorship

TRG conceived and supervised the study as well as acquired funding. MKS curated the data and performed the main analyses. JZ inspired the study and supplied xQTL analyses. MKS, GDS, JZ and TRG wrote the manuscript.

Acknowledgements

Not applicable

For peer review only

References

1 Ference BA, Holmes M V, Smith GD. Using Mendelian Randomization to Improve the Design of Randomized Trials. *Cold Spring Harb Perspect Med* 2021;**11**.http://perspectivesinmedicine.cshlp.org/content/11/7/a040980.abstract

2 Davey Smith G, Ebrahim S. 'Mendelian randomization': can genetic epidemiology contribute to understanding environmental determinants of disease?*. *Int J Epidemiol* 2003;**32**:1–22. doi:10.1093/ije/dyg070

3 Sanderson E, Glymour MM, Holmes M V, *et al.* Mendelian randomization. *Nat Rev Methods Prim* 2022;**2**:6. doi:10.1038/s43586-021-00092-5

4 Hingorani A, Humphries S. Nature's randomised trials. *Lancet* 2005;**366**:1906–8. doi:10.1016/S0140-6736(05)67767-7

5 Davey Smith G, Holmes M V, Davies NM, *et al.* Mendel's laws, Mendelian randomization and causal inference in observational data: substantive and nomenclatural issues. *Eur J Epidemiol* 2020;**35**:99–111. doi:10.1007/s10654-020-00622-7

6 Davies NM, Holmes M V., Davey Smith G. Reading Mendelian randomisation studies: A guide, glossary, and checklist for clinicians. *BMJ* 2018;**362**. doi:10.1136/bmj.k601

7 Swanson SA, Tiemeier H, Ikram MA, *et al.* Nature as a Trialist?: Deconstructing the Analogy Between Mendelian Randomization and Randomized Trials. *Epidemiology* 2017;**28**:653–9. doi:10.1097/EDE.0000000000000699

8 Martin L, Hutchens M, Hawkins C. Trial watch: Clinical trial cycle times continue to increase despite industry efforts. *Nat Rev Drug Discov* 2017;**16**:157. doi:10.1038/nrd.2017.21

9 Egger M, Buitrago-Garcia D, Smith GD. Systematic Reviews of Epidemiological Studies of Etiology and Prevalence. In: Egger M, Higgins JPT, Smith GD, eds. *Systematic Reviews in Health Care: Meta-analyses in Context*. Wiley-Blackwell 2022. Chapter 19.

10 Voight BF, Peloso GM, Orho-Melander M, *et al.* Plasma HDL cholesterol and risk of myocardial infarction: a mendelian randomisation study. *Lancet* 2012;**380**:572–80.

For peer review only - http://bmjopen.bmj.com/site/about/guidelines.xhtml

doi:https://doi.org/10.1016/S0140-6736(12)60312-2

11 Verdoia M, Schaffer A, Suryapranata H, *et al.* Effects of HDL-modifiers on cardiovascular outcomes: A meta-analysis of randomized trials. *Nutr Metab Cardiovasc Dis* 2015;**25**:9–23. doi:https://doi.org/10.1016/j.numecd.2014.09.003

12 Davey Smith G, Phillips AN. Correlation without a cause: an epidemiological odyssey. *Int J Epidemiol* 2020;**49**:4–14. doi:10.1093/ije/dyaa016

13 Yarmolinsky J, Bonilla C, Haycock PC, *et al.* Circulating Selenium and Prostate Cancer Risk: A Mendelian Randomization Analysis. *JNCI J Natl Cancer Inst* 2018;**110**:1035–8. doi:10.1093/jnci/djy081

14 Lippman SM, Klein EA, Goodman PJ, *et al.* Effect of Selenium and Vitamin E on Risk of Prostate Cancer and Other Cancers: The Selenium and Vitamin E Cancer Prevention Trial (SELECT). *JAMA* 2009;**301**:39–51. doi:10.1001/jama.2008.864

15 A. FB, Wonsuk Y, Issa A, *et al.* Effect of Long-Term Exposure to Lower Low-Density Lipoprotein Cholesterol Beginning Early in Life on the Risk of Coronary Heart Disease. *J Am Coll Cardiol* 2012;**60**:2631–9. doi:10.1016/j.jacc.2012.09.017

16 Ference BA, Robinson JG, Brook RD, *et al.* Variation in PCSK9 and HMGCR and Risk of Cardiovascular Disease and Diabetes. *N Engl J Med* 2016;**375**:2144–53. doi:10.1056/NEJMoa1604304

17 Hegele RA. Editorial comment: when Mendelian randomization goes astray. *Curr Opin Lipidol* 2021;**32**.https://journals.lww.com/co-lipidology/Fulltext/2021/04000/Editorial_comment__when_Mendelian_randomization.2.aspx

18 Turner RM, Spiegelhalter DJ, Smith GCS, *et al.* Bias modelling in evidence synthesis. *J R Stat Soc Ser A (Statistics Soc* 2009;**172**:21–47. doi:https://doi.org/10.1111/j.1467-985X.2008.00547.x

19 Morris TT, Heron J, Sanderson E, *et al.* Interpretation of mendelian randomization using one measure of an exposure that varies over time. *medRxiv* Published Online First: 2021. doi:10.1101/2021.11.18.21266515

20 Victora CG, Habicht J-P, Bryce J. Evidence-Based Public Health: Moving Beyond Randomized Trials. *Am J Public Health* 2004;**94**:400–5. doi:10.2105/AJPH.94.3.400

21 Chekroud AM. A Mendelian Randomization Approach for Assessing the Relationship Between Physical Activity and Depression. *JAMA Psychiatry* 2019;**76**:361–2. doi:10.1001/jamapsychiatry.2018.3870

22 Harrison S, Dixon P, Jones HE, *et al.* Long-term cost-effectiveness of interventions for obesity: A mendelian randomisation study. *PLOS Med* 2021;**18**:e1003725.https://doi.org/10.1371/journal.pmed.1003725

23 Holmes M V, Richardson TG, Ference BA, *et al.* Integrating genomics with biomarkers and therapeutic targets to invigorate cardiovascular drug development. *Nat Rev Cardiol* 2021;**18**:435–53. doi:10.1038/s41569-020-00493-1

24 Borén J, Chapman MJ, Krauss RM, *et al.* Low-density lipoproteins cause atherosclerotic cardiovascular disease: pathophysiological, genetic, and therapeutic insights: a consensus statement from the European Atherosclerosis Society Consensus Panel. *Eur Heart J* 2020;**41**:2313–30. doi:10.1093/eurheartj/ehz962

For peer review only - http://bmjopen.bmj.com/site/about/guidelines.xhtml

25 Munafò MR, Higgins JPT, Smith GD. Triangulating Evidence through the Inclusion of Genetically Informed Designs. *Cold Spring Harb Perspect Med* 2021;**11**. doi:10.1101/cshperspect.a040659

26 Lawlor DA, Tilling K, Smith GD. Triangulation in aetiological epidemiology. *Int J Epidemiol* 2016;**45**:1866–86. doi:10.1093/ije/dyw314

27 Zarin DA, Tse T, Williams RJ, *et al.* The ClinicalTrials.gov Results Database — Update and Key Issues. *N Engl J Med* 2011;**364**:852–60. doi:10.1056/NEJMsa1012065

28 Liu Y, Elsworth B, Erola P, *et al.* EpiGraphDB: a database and data mining platform for health data science. *Bioinformatics* 2020;:0–0.https://academic.oup.com/bioinformatics/advance-article/doi/10.1093/bioinformatics/btaa961/5962087

29 Tasneem A, Aberle L, Ananth H, *et al.* The Database for Aggregate Analysis of ClinicalTrials.gov (AACT) and Subsequent Regrouping by Clinical Specialty. *PLoS One* 2012;**7**:e33677.https://doi.org/10.1371/journal.pone.0033677

30 Tenaerts P, Madre L, Archdeacon P, *et al.* The Clinical Trials Transformation Initiative: innovation through collaboration. *Nat Rev Drug Discov* 2014;**13**:797–8. doi:10.1038/nrd4442

31 Ochoa D, Hercules A, Carmona M, *et al.* Open Targets Platform: supporting systematic drug–target identification and prioritisation. *Nucleic Acids Res* 2020;**49**:D1302–10. doi:10.1093/nar/gkaa1027

32 Wishart DS, Feunang YD, Guo AC, *et al.* DrugBank 5.0: a major update to the DrugBank database for 2018. *Nucleic Acids Res* 2017;**46**:D1074–82. doi:10.1093/nar/gkx1037

33 Zheng J, Haberland V, Baird D, *et al.* Phenome-wide Mendelian randomization mapping the influence of the plasma proteome on complex diseases. *Nat Genet* 2020;**52**:1122–31. doi:10.1038/s41588-020-0682-6

34 Kilicoglu H, Shin D, Fiszman M, *et al.* SemMedDB: a PubMed-scale repository of biomedical semantic predications. *Bioinformatics* 2012;**28**:3158–60. doi:10.1093/bioinformatics/bts591

35 Elsworth B, Gaunt TR. MELODI Presto: a fast and agile tool to explore semantic triples derived from biomedical literature. *Bioinformatics* 2020;**37**:583–5. doi:10.1093/bioinformatics/btaa726

36 Mokdad AH, Marks JS, Stroup DF, *et al.* Actual Causes of Death in the United States, 2000. *JAMA* 2004;**291**:1238–45. doi:10.1001/jama.291.10.1238

37 Carnegie R, Zheng J, Sallis HM, *et al.* Mendelian randomisation for nutritional psychiatry. *The Lancet Psychiatry* 2020;**7**:208–16. doi:https://doi.org/10.1016/S2215-0366(19)30293-7

38 Cicero AFG, Tartagni E, Ertek S. Safety and tolerability of injectable lipid-lowering drugs: a review of available clinical data. *Expert Opin Drug Saf* 2014;**13**:1023–30. doi:10.1517/14740338.2014.932348

39 Luo J, Wu S-J, Lacy ER, *et al.* Structural Basis for the Dual Recognition of IL-12 and IL-23 by Ustekinumab. *J Mol Biol* 2010;**402**:797–812. doi:https://doi.org/10.1016/j.jmb.2010.07.046

40 Hansen RA, Gartlehner G, Webb AP, *et al.* Efficacy and safety of donepezil, galantamine, and rivastigmine for the treatment of Alzheimer's disease: a systematic review and meta-analysis. *Clin Interv Aging* 2008;**3**:211–25.https://pubmed.ncbi.nlm.nih.gov/18686744

41 Ference BA, Kastelein JJP, Ginsberg HN, *et al.* Association of Genetic Variants Related to CETP Inhibitors and Statins With Lipoprotein Levels and Cardiovascular Risk. *JAMA* 2017;**318**:947–

For peer review only - http://bmjopen.bmj.com/site/about/guidelines.xhtml

For peer review only

56. doi:10.1001/jama.2017.11467

42 Piepho RW. Overview of the angiotensin-converting-enzyme inhibitors. *Am J Heal Pharm* 2000;**57**:S3–7. doi:10.1093/ajhp/57.suppl_1.S3

43 Marshall IJ, Nye B, Kuiper J, *et al.* Trialstreamer: A living, automatically updated database of clinical trial reports. *J Am Med Informatics Assoc* 2020;**27**:1903–12. doi:10.1093/jamia/ocaa163

44 Mokry LE, Ross S, Ahmad OS, *et al.* Vitamin D and Risk of Multiple Sclerosis: A Mendelian Randomization Study. *PLOS Med* 2015;**12**:e1001866.https://doi.org/10.1371/journal.pmed.1001866

45 James E, Dobson R, Kuhle J, *et al.* The effect of vitamin D-related interventions on multiple sclerosis relapses: a meta-analysis. *Mult Scler J* 2013;**19**:1571–9. doi:10.1177/1352458513489756

46 Hempel S, Graham GD, Fu N, *et al.* A systematic review of the effects of modifiable risk factor interventions on the progression of multiple sclerosis. *Mult Scler J* 2017;**23**:513–24. doi:10.1177/1352458517690271

47 Zhuang Z, Gao M, Yang R, *et al.* Association of physical activity, sedentary behaviours and sleep duration with cardiovascular diseases and lipid profiles: a Mendelian randomization analysis. *Lipids Health Dis* 2020;**19**:86. doi:10.1186/s12944-020-01257-z

48 Xu F, Zhang Q, Wang L-K, *et al.* Estimates of the effects of physical activity on osteoporosis using multivariable Mendelian randomization analysis. *Osteoporos Int* 2021;**32**:1359–67. doi:10.1007/s00198-020-05786-2

49 Kelley GA, Kelley KS, Tran ZV. Walking, lipids, and lipoproteins: a meta-analysis of randomized controlled trials. *Prev Med (Baltim)* 2004;**38**:651–61. doi:https://doi.org/10.1016/j.ypmed.2003.12.012

50 Kelley GA, Kelley KS. Aerobic exercise and lipids and lipoproteins in men: a meta-analysis of randomized controlled trials. *J Men's Heal Gend* 2006;**3**:61–70. doi:10.1016/j.jmhg.2005.09.003

51 Kelley GA, Kelley KS, Tran ZV. Aerobic Exercise and Lipids and Lipoproteins in Women: A Meta-Analysis of Randomized Controlled Trials. *J Women's Heal* 2004;**13**:1148–64. doi:10.1089/jwh.2004.13.1148

52 Kelley GA, Kelley KS. Impact of progressive resistance training on lipids and lipoproteins in adults: A meta-analysis of randomized controlled trials. *Prev Med (Baltim)* 2009;**48**:9–19. doi:https://doi.org/10.1016/j.ypmed.2008.10.010

53 Mosti MP, Carlsen T, Aas E, *et al.* Maximal Strength Training Improves Bone Mineral Density and Neuromuscular Performance in Young Adult Women. *J Strength Cond Res* 2014;**28**.https://journals.lww.com/nsca-jscr/Fulltext/2014/10000/Maximal_Strength_Training_Improves_Bone_Mineral.30.aspx

54 Allison SJ, Folland JP, Rennie WJ, *et al.* High impact exercise increased femoral neck bone mineral density in older men: A randomised unilateral intervention. *Bone* 2013;**53**:321–8. doi:https://doi.org/10.1016/j.bone.2012.12.045

55 Katzel LI, Bleecker ER, Colman EG, *et al.* Effects of weight loss vs aerobic exercise training on risk factors for coronary disease in healthy, obese, middle-aged and older men. A randomized controlled trial. *JAMA* 1995;**274**:1915–21.

For peer review only - http://bmjopen.bmj.com/site/about/guidelines.xhtml

doi:10.1001/jama.1995.03530240025035

56 Çolak Y, Afzal S, Nordestgaard BG. 25-Hydroxyvitamin D and Risk of Osteoporotic Fractures: Mendelian Randomization Analysis in 2 Large Population-Based Cohorts. *Clin Chem* 2020;**66**:676–85. doi:10.1093/clinchem/hvaa049

57 Bischoff-Ferrari HA, Willett WC, Orav EJ, *et al.* A Pooled Analysis of Vitamin D Dose Requirements for Fracture Prevention. *N Engl J Med* 2012;**367**:40–9. doi:10.1056/NEJMoa1109617

58 Bischoff-Ferrari HA, Willett WC, Wong JB, *et al.* Prevention of Nonvertebral Fractures With Oral Vitamin D and Dose Dependency: A Meta-analysis of Randomized Controlled Trials. *Arch Intern Med* 2009;**169**:551–61. doi:10.1001/archinternmed.2008.600

59 Bischoff-Ferrari HA, Willett WC, Wong JB, *et al.* Fracture Prevention With Vitamin D SupplementationA Meta-analysis of Randomized Controlled Trials. *JAMA* 2005;**293**:2257–64. doi:10.1001/jama.293.18.2257

60 Papiol S, Schmitt A, Maurus I, *et al.* Association Between Physical Activity and Schizophrenia: Results of a 2-Sample Mendelian Randomization Analysis. *JAMA Psychiatry* 2021;**78**:441–4. doi:10.1001/jamapsychiatry.2020.3946

61 Sun H, Gao X, Que X, *et al.* The causal relationships of device-measured physical activity with bipolar disorder and schizophrenia in adults: A 2-Sample mendelian randomization study. *J Affect Disord* 2020;**263**:598–604. doi:https://doi.org/10.1016/j.jad.2019.11.034

62 Rosenbaum S, Tiedemann A, Sherrington C, *et al.* Physical activity interventions for people with mental illness: A systematic review and meta-analysis. J. Clin. Psychiatry. 2014;**75**:964–74. doi:10.4088/JCP.13r08765

63 Firth J, Cotter J, Elliott R, *et al.* A systematic review and meta-analysis of exercise interventions in schizophrenia patients. *Psychol Med* 2015;**45**:1343–1361. doi:10.1017/S0033291714003110

64 Sabe M, Kaiser S, Sentissi O. Physical exercise for negative symptoms of schizophrenia: Systematic review of randomized controlled trials and meta-analysis. *Gen Hosp Psychiatry* 2020;**62**:13–20. doi:https://doi.org/10.1016/j.genhosppsych.2019.11.002

65 Williamson JD, Supiano MA, Applegate WB, *et al.* Intensive vs Standard Blood Pressure Control and Cardiovascular Disease Outcomes in Adults Aged ≥75 Years: A Randomized Clinical Trial. *JAMA* 2016;**315**:2673–82. doi:10.1001/jama.2016.7050

66 Brunström M, Carlberg B. Association of Blood Pressure Lowering With Mortality and Cardiovascular Disease Across Blood Pressure Levels: A Systematic Review and Meta-analysis. *JAMA Intern Med* 2018;**178**:28–36. doi:10.1001/jamainternmed.2017.6015

67 Rahimi K, Bidel Z, Nazarzadeh M, *et al.* Age-stratified and blood-pressure-stratified effects of blood-pressure-lowering pharmacotherapy for the prevention of cardiovascular disease and death: an individual participant-level data meta-analysis. *Lancet* 2021;**398**:1053–64. doi:10.1016/S0140-6736(21)01921-8

68 Gill D, Georgakis MK, Zuber V, *et al.* Genetically Predicted Midlife Blood Pressure and Coronary Artery Disease Risk: Mendelian Randomization Analysis. *J Am Heart Assoc* 2020;**9**:e016773. doi:10.1161/JAHA.120.016773

69 Malik R, Georgakis MK, Vujkovic M, *et al.* Relationship Between Blood Pressure and Incident Cardiovascular Disease: Linear and Nonlinear Mendelian Randomization Analyses.

For peer review only - http://bmjopen.bmj.com/site/about/guidelines.xhtml

Hypertension 2021;**77**:2004–13. doi:10.1161/HYPERTENSIONAHA.120.16534

70 Wan EYF, Fung WT, Schooling CM, *et al.* Blood Pressure and Risk of Cardiovascular Disease in UK Biobank. *Hypertension* 2021;**77**:367–75. doi:10.1161/HYPERTENSIONAHA.120.16138

71 Riaz H, Khan MS, Siddiqi TJ, *et al.* Association Between Obesity and Cardiovascular Outcomes: A Systematic Review and Meta-analysis of Mendelian Randomization Studies. *JAMA Netw Open* 2018;**1**:e183788–e183788. doi:10.1001/jamanetworkopen.2018.3788

72 Larsson SC, Bäck M, Rees JMB, *et al.* Body mass index and body composition in relation to 14 cardiovascular conditions in UK Biobank: a Mendelian randomization study. *Eur Heart J* 2020;**41**:221–6. doi:10.1093/eurheartj/ehz388

73 Cardiovascular Effects of Intensive Lifestyle Intervention in Type 2 Diabetes. *N Engl J Med* 2013;**369**:145–54. doi:10.1056/NEJMoa1212914

74 Mather AS, Rodriguez C, Guthrie MF, *et al.* Effects of exercise on depressive symptoms in older adults with poorly responsive depressive disorder: Randomised controlled trial. *Br J Psychiatry* 2002;**180**:411–415. doi:10.1192/bjp.180.5.411

75 Choi KW, Chen C-Y, Stein MB, *et al.* Assessment of Bidirectional Relationships Between Physical Activity and Depression Among Adults: A 2-Sample Mendelian Randomization Study. *JAMA Psychiatry* 2019;**76**:399–408. doi:10.1001/jamapsychiatry.2018.4175

76 Choi KW, Stein MB, Nishimi KM, *et al.* An Exposure-Wide and Mendelian Randomization Approach to Identifying Modifiable Factors for the Prevention of Depression. *Am J Psychiatry* 2020;**177**:944–54. doi:10.1176/appi.ajp.2020.19111158

77 Veale D, Le Fevre K, Pantelis C, *et al.* Aerobic exercise in the adjunctive treatment of depression: a randomized controlled trial. *J R Soc Med* 1992;**85**:541–4.https://pubmed.ncbi.nlm.nih.gov/1433121

78 Schuch FB, Vancampfort D, Richards J, *et al.* Exercise as a treatment for depression: A meta-analysis adjusting for publication bias. *J Psychiatr Res* 2016;**77**:42–51. doi:https://doi.org/10.1016/j.jpsychires.2016.02.023

79 Kvam S, Kleppe CL, Nordhus IH, *et al.* Exercise as a treatment for depression: A meta-analysis. *J Affect Disord* 2016;**202**:67–86. doi:https://doi.org/10.1016/j.jad.2016.03.063

80 Gordon BR, McDowell CP, Hallgren M, *et al.* Association of Efficacy of Resistance Exercise Training With Depressive Symptoms: Meta-analysis and Meta-regression Analysis of Randomized Clinical Trials. *JAMA Psychiatry* 2018;**75**:566–76. doi:10.1001/jamapsychiatry.2018.0572

81 Manousaki D, Paternoster L, Standl M, *et al.* Vitamin D levels and susceptibility to asthma, elevated immunoglobulin E levels, and atopic dermatitis: A Mendelian randomization study. *PLOS Med* 2017;**14**:1–16. doi:10.1371/journal.pmed.1002294

82 Drodge DR, Budu-Aggrey A, Paternoster L. Causal Analysis Shows Evidence of Atopic Dermatitis Leading to an Increase in Vitamin D Levels. *J Invest Dermatol* 2021;**141**:1339–41. doi:https://doi.org/10.1016/j.jid.2020.09.013

83 Camargo CA, Ganmaa D, Sidbury R, *et al.* Randomized trial of vitamin D supplementation for winter-related atopic dermatitis in children. *J Allergy Clin Immunol* 2014;**134**:831-835.e1. doi:https://doi.org/10.1016/j.jaci.2014.08.002

84 Sidbury R, Sullivan AF, Thadhani RI, *et al.* Randomized controlled trial of vitamin D supplementation for winter-related atopic dermatitis in Boston: a pilot study. *Br J Dermatol*

For peer review only - http://bmjopen.bmj.com/site/about/guidelines.xhtml

2008;**159**:245—247. doi:10.1111/j.1365-2133.2008.08601.x

85 Amestejani M, Salehi BS, Vasigh M, *et al.* Vitamin D supplementation in the treatment of atopic dermatitis: a clinical trial study. *J Drugs Dermatology* 2012;**11**.

86 Javanbakht MH, Keshavarz SA, Djalali M, *et al.* Randomized controlled trial using vitamins E and D supplementation in atopic dermatitis. *J Dermatolog Treat* 2011;**22**:144–50. doi:10.3109/09546630903578566

87 Meisinger C, Linseisen J, Leitzmann M, *et al.* Association of physical activity and sedentary behavior with type 2 diabetes and glycemic traits: a two-sample Mendelian randomization study. *BMJ Open Diabetes Res Care* 2020;**8**. doi:10.1136/bmjdrc-2020-001896

88 Shah SZA, Karam JA, Zeb A, *et al.* Movement is Improvement: The Therapeutic Effects of Exercise and General Physical Activity on Glycemic Control in Patients with Type 2 Diabetes Mellitus: A Systematic Review and Meta-Analysis of Randomized Controlled Trials. *Diabetes Ther* 2021;**12**:707–32. doi:10.1007/s13300-021-01005-1

89 Corbin LJ, Richmond RC, Wade KH, *et al.* BMI as a Modifiable Risk Factor for Type 2 Diabetes: Refining and Understanding Causal Estimates Using Mendelian Randomization. *Diabetes* 2016;**65**:3002–7. doi:10.2337/db16-0418

90 Dale CE, Fatemifar G, Palmer TM, *et al.* Causal Associations of Adiposity and Body Fat Distribution With Coronary Heart Disease, Stroke Subtypes, and Type 2 Diabetes Mellitus. *Circulation* 2017;**135**:2373–88. doi:10.1161/CIRCULATIONAHA.116.026560

91 Cheng L, Zhuang H, Ju H, *et al.* Exposing the causal effect of body mass index on the risk of type 2 diabetes mellitus: a mendelian randomization study. *Front Genet* 2019;**10**:94.

92 Wainberg M, Mahajan A, Kundaje A, *et al.* Homogeneity in the association of body mass index with type 2 diabetes across the UK Biobank: A Mendelian randomization study. *PLOS Med* 2019;**16**:e1002982.https://doi.org/10.1371/journal.pmed.1002982

93 Xu H, Jin C, Guan Q. Causal Effects of Overall and Abdominal Obesity on Insulin Resistance and the Risk of Type 2 Diabetes Mellitus: A Two-Sample Mendelian Randomization Study. *Front Genet* 2020;**11**:603. doi:10.3389/fgene.2020.00603

94 Pan X-R, Li G-W, Hu Y-H, *et al.* Effects of Diet and Exercise in Preventing NIDDM in People With Impaired Glucose Tolerance: The Da Qing IGT and Diabetes Study. *Diabetes Care* 1997;**20**:537–44. doi:10.2337/diacare.20.4.537

95 Tuomilehto J, Lindström J, Eriksson JG, *et al.* Prevention of Type 2 Diabetes Mellitus by Changes in Lifestyle among Subjects with Impaired Glucose Tolerance. *N Engl J Med* 2001;**344**:1343–50. doi:10.1056/NEJM200105033441801

96 Reduction in the Incidence of Type 2 Diabetes with Lifestyle Intervention or Metformin. *N Engl J Med* 2002;**346**:393–403. doi:10.1056/NEJMoa012512

97 Sabatine MS, Giugliano RP, Wiviott SD, *et al.* Efficacy and Safety of Evolocumab in Reducing Lipids and Cardiovascular Events. *N Engl J Med* 2015;**372**:1500–9. doi:10.1056/NEJMoa1500858

98 Sabatine MS, Giugliano RP, Keech AC, *et al.* Evolocumab and Clinical Outcomes in Patients with Cardiovascular Disease. *N Engl J Med* 2017;**376**:1713–22. doi:10.1056/NEJMoa1615664

99 He J, Whelton PK, Appel LJ, *et al.* Long-Term Effects of Weight Loss and Dietary Sodium Reduction on Incidence of Hypertension. *Hypertension* 2000;**35**:544–9. doi:10.1161/01.HYP.35.2.544

For peer review only - http://bmjopen.bmj.com/site/about/guidelines.xhtml

100 Long-Term Weight Loss and Changes in Blood Pressure: Results of the Trials of Hypertension Prevention, Phase II. *Ann Intern Med* 2001;**134**:1–11. doi:10.7326/0003-4819-134-1-200101020-00007

101 Puddey IB, Beilin LJ, Vandongen R, *et al.* Evidence for a direct effect of alcohol consumption on blood pressure in normotensive men. A randomized controlled trial. *Hypertension* 1985;**7**:707–13. doi:10.1161/01.HYP.7.5.707

102 Puddey IB, Beilin LJ, Vandongen R. REGULAR ALCOHOL USE RAISES BLOOD PRESSURE IN TREATED HYPERTENSIVE SUBJECTS: A Randomised Controlled Trial. *Lancet* 1987;**329**:647–51. doi:https://doi.org/10.1016/S0140-6736(87)90413-2

103 Xin X, He J, Frontini MG, *et al.* Effects of Alcohol Reduction on Blood Pressure. *Hypertension* 2001;**38**:1112–7. doi:10.1161/hy1101.093424

104 Puddey IB, Parker M, Beilin LJ, *et al.* Effects of alcohol and caloric restrictions on blood pressure and serum lipids in overweight men. *Hypertension* 1992;**20**:533–41. doi:10.1161/01.HYP.20.4.533

105 Helgadóttir B, Hallgren M, Ekblom Ö, *et al.* Training fast or slow? Exercise for depression: A randomized controlled trial. *Prev Med (Baltim)* 2016;**91**:123–31. doi:https://doi.org/10.1016/j.ypmed.2016.08.011

106 Ma C, Avenell A, Bolland M, *et al.* Effects of weight loss interventions for adults who are obese on mortality, cardiovascular disease, and cancer: systematic review and meta-analysis. *BMJ* 2017;**359**. doi:10.1136/bmj.j4849

107 Timpson NJ, Harbord R, Davey Smith G, *et al.* Does Greater Adiposity Increase Blood Pressure and Hypertension Risk? *Hypertension* 2009;**54**:84–90. doi:10.1161/HYPERTENSIONAHA.109.130005

108 Lyall DM, Celis-Morales C, Ward J, *et al.* Association of Body Mass Index With Cardiometabolic Disease in the UK Biobank: A Mendelian Randomization Study. *JAMA Cardiol* 2017;**2**:882–9. doi:10.1001/jamacardio.2016.5804

109 Lee M-R, Lim Y-H, Hong Y-C. Causal association of body mass index with hypertension using a Mendelian randomization design. *Medicine (Baltimore)* 2018;**97**:e11252–e11252. doi:10.1097/MD.0000000000011252

110 van Oort S, Beulens JWJ, van Ballegooijen AJ, *et al.* Association of Cardiovascular Risk Factors and Lifestyle Behaviors With Hypertension. *Hypertension* 2020;**76**:1971–9. doi:10.1161/HYPERTENSIONAHA.120.15761

111 Rivera CM, Ren B. Mapping human epigenomes. *Cell* 2013;**155**:39–55. doi:10.1016/j.cell.2013.09.011

112 Wang N, Wang C, Chen X, *et al.* Vitamin D, prediabetes and type 2 diabetes: bidirectional Mendelian randomization analysis. *Eur J Nutr* 2020;**59**:1379–88. doi:10.1007/s00394-019-01990-x

113 Salehpour A, Shidfar F, Hosseinpanah F, *et al.* Does vitamin D3 supplementation improve glucose homeostasis in overweight or obese women? A double-blind, randomized, placebo-controlled clinical trial. *Diabet Med* 2013;**30**:1477–81. doi:https://doi.org/10.1111/dme.12273

114 Sollid ST, Hutchinson MYS, Fuskev\r ag OM, *et al.* No Effect of High-Dose Vitamin D Supplementation on Glycemic Status or Cardiovascular Risk Factors in Subjects With

For peer review only - http://bmjopen.bmj.com/site/about/guidelines.xhtml

Prediabetes. *Diabetes Care* 2014;**37**:2123–31. doi:10.2337/dc14-0218

115 Zuk A, Fitzpatrick T, Rosella LC. Effect of Vitamin D3 Supplementation on Inflammatory Markers and Glycemic Measures among Overweight or Obese Adults: A Systematic Review of Randomized Controlled Trials. *PLoS One* 2016;**11**:e0154215.https://doi.org/10.1371/journal.pone.0154215

116 Wright ME, Peters U, Gunter MJ, *et al.* Association of Variants in Two Vitamin E Transport Genes with Circulating Vitamin E Concentrations and Prostate Cancer Risk. *Cancer Res* 2009;**69**:1429–38. doi:10.1158/0008-5472.CAN-08-2343

117 Cheng W-W, Wang Z-K, Shangguan H-F, *et al.* Are vitamins relevant to cancer risks? A Mendelian randomization investigation. *Nutrition* 2020;**78**:110870. doi:https://doi.org/10.1016/j.nut.2020.110870

118 Jiang L, Yang K, Tian J, *et al.* Efficacy of Antioxidant Vitamins and Selenium Supplement in Prostate Cancer Prevention: A Meta-Analysis of Randomized Controlled Trials. *Nutr Cancer* 2010;**62**:719–27. doi:10.1080/01635581.2010.494335

119 Gaziano JM, Glynn RJ, Christen WG, *et al.* Vitamins E and C in the Prevention of Prostate and Total Cancer in Men: The Physicians' Health Study II Randomized Controlled Trial. *JAMA* 2009;**301**:52–62. doi:10.1001/jama.2008.862

120 Wang L, Sesso HD, Glynn RJ, *et al.* Vitamin E and C supplementation and risk of cancer in men: posttrial follow-up in the Physicians' Health Study II randomized trial. *Am J Clin Nutr* 2014;**100**:915–23. doi:10.3945/ajcn.114.085480

121 Heinonen OP, Koss L, Albanes D, *et al.* Prostate Cancer and Supplementation With α-Tocopherol and β-Carotene: Incidence and Mortality in a Controlled Trial. *JNCI J Natl Cancer Inst* 1998;**90**:440–6. doi:10.1093/jnci/90.6.440

122 Vieth R, Kimball S, Hu A, *et al.* Randomized comparison of the effects of the vitamin D3adequate intake versus 100 mcg (4000 IU) per day on biochemical responses and the wellbeing of patients. *Nutr J* 2004;**3**:8. doi:10.1186/1475-2891-3-8

123 Michaëlsson K, Melhus H, Larsson SC. Serum 25-Hydroxyvitamin D Concentrations and Major Depression: A Mendelian Randomization Study. *Nutrients* 2018;**10**. doi:10.3390/nu10121987

124 Libuda L, Laabs B-H, Ludwig C, *et al.* Vitamin D and the Risk of Depression: A Causal Relationship? Findings from a Mendelian Randomization Study. *Nutrients* 2019;**11**. doi:10.3390/nu11051085

125 Mulugeta A, Lumsden A, Hyppönen E. Relationship between Serum 25(OH)D and Depression: Causal Evidence from a Bi-Directional Mendelian Randomization Study. *Nutrients* 2021;**13**. doi:10.3390/nu13010109

126 Milaneschi Y, Peyrot WJ, Nivard MG, *et al.* A role for vitamin D and omega-3 fatty acids in major depression? An exploration using genomics. *Transl Psychiatry* 2019;**9**:219. doi:10.1038/s41398-019-0554-y

127 Bertone-Johnson ER, Powers SI, Spangler L, *et al.* Vitamin D Supplementation and Depression in the Women's Health Initiative Calcium and Vitamin D Trial. *Am J Epidemiol* 2012;**176**:1–13. doi:10.1093/aje/kwr482

128 Kjærgaard M, Waterloo K, Wang CEA, *et al.* Effect of vitamin D supplement on depression scores in people with low levels of serum 25-hydroxyvitamin D: nested case—control study and randomised clinical trial. *Br J Psychiatry* 2012;**201**:360–368.

For peer review only - http://bmjopen.bmj.com/site/about/guidelines.xhtml

doi:10.1192/bjp.bp.111.104349

129 Skaaby T, Husemoen LLN, Martinussen T, *et al.* Vitamin D Status, Filaggrin Genotype, and Cardiovascular Risk Factors: A Mendelian Randomization Approach. *PLoS One* 2013;**8**:e57647.https://doi.org/10.1371/journal.pone.0057647

130 Kunutsor SK, Burgess S, Munroe PB, *et al.* Vitamin D and high blood pressure: causal association or epiphenomenon? *Eur J Epidemiol* 2014;**29**:1–14. doi:10.1007/s10654-013-9874-z

131 Vimaleswaran KS, Cavadino A, Berry DJ, *et al.* Association of vitamin D status with arterial blood pressure and hypertension risk: a mendelian randomisation study. *Lancet Diabetes Endocrinol* 2014;**2**:719–29. doi:https://doi.org/10.1016/S2213-8587(14)70113-5

132 Rosoff DB, Davey Smith G, Mehta N, *et al.* Evaluating the relationship between alcohol consumption, tobacco use, and cardiovascular disease: A multivariable Mendelian randomization study. *PLOS Med* 2020;**17**:e1003410.https://doi.org/10.1371/journal.pmed.1003410

133 Gepner AD, Piper ME, Johnson HM, *et al.* Effects of smoking and smoking cessation on lipids and lipoproteins: Outcomes from a randomized clinical trial. *Am Heart J* 2011;**161**:145–51. doi:https://doi.org/10.1016/j.ahj.2010.09.023

134 Maeda K, Noguchi Y, Fukui T. The effects of cessation from cigarette smoking on the lipid and lipoprotein profiles: a meta-analysis. *Prev Med (Baltim)* 2003;**37**:283–90. doi:https://doi.org/10.1016/S0091-7435(03)00110-5

135 Åsvold BO, Bjørngaard JH, Carslake D, *et al.* Causal associations of tobacco smoking with cardiovascular risk factors: a Mendelian randomization analysis of the HUNT Study in Norway. *Int J Epidemiol* 2014;**43**:1458–70. doi:10.1093/ije/dyu113

136 Jee SH, He J, Appel LJ, *et al.* Coffee Consumption and Serum Lipids: A Meta-Analysis of Randomized Controlled Clinical Trials. *Am J Epidemiol* 2001;**153**:353–62. doi:10.1093/aje/153.4.353

137 Cai L, Ma D, Zhang Y, *et al.* The effect of coffee consumption on serum lipids: a meta-analysis of randomized controlled trials. *Eur J Clin Nutr* 2012;**66**:872–7. doi:10.1038/ejcn.2012.68

138 Du Y, Lv Y, Zha W, *et al.* Effect of coffee consumption on dyslipidemia: A meta-analysis of randomized controlled trials. *Nutr Metab Cardiovasc Dis* 2020;**30**:2159–70. doi:https://doi.org/10.1016/j.numecd.2020.08.017

139 Urgert R, Katan MB. THE CHOLESTEROL-RAISING FACTOR FROM COFFEE BEANS. *Annu Rev Nutr* 1997;**17**:305–24. doi:10.1146/annurev.nutr.17.1.305

140 Zhou A, Hyppönen E. Habitual coffee intake and plasma lipid profile: Evidence from UK Biobank. *Clin Nutr* 2021;**40**:4404–13. doi:https://doi.org/10.1016/j.clnu.2020.12.042

141 Lebeau PF, Byun JH, Platko K, *et al.* Caffeine blocks SREBP2-induced hepatic PCSK9 expression to enhance LDLR-mediated cholesterol clearance. *Nat Commun* 2022;**13**:770. doi:10.1038/s41467-022-28240-9

142 Kwok MK, Leung GM, Schooling CM. Habitual coffee consumption and risk of type 2 diabetes, ischemic heart disease, depression and Alzheimer's disease: a Mendelian randomization study. *Sci Rep* 2016;**6**:36500. doi:10.1038/srep36500

143 Nordestgaard AT, Thomsen M, Nordestgaard BG. Coffee intake and risk of obesity, metabolic syndrome and type 2 diabetes: a Mendelian randomization study. *Int J Epidemiol*

For peer review only

2015;**44**:551–65. doi:10.1093/ije/dyv083

144 Zarin DA, Tse T, Williams RJ, *et al.* Trial Reporting in ClinicalTrials.gov — The Final Rule. *N Engl J Med* 2016;**375**:1998–2004. doi:10.1056/NEJMsr1611785

145 Anderson ML, Chiswell K, Peterson ED, *et al.* Compliance with Results Reporting at ClinicalTrials.gov. *N Engl J Med* 2015;**372**:1031–9. doi:10.1056/NEJMsa1409364

146 DeVito NJ, Bacon S, Goldacre B. Compliance with legal requirement to report clinical trial results on ClinicalTrials.gov: a cohort study. *Lancet* 2020;**395**:361–9. doi:10.1016/S0140-6736(19)33220-9

147 Hwang TJ, Carpenter D, Lauffenburger JC, *et al.* Failure of Investigational Drugs in Late-Stage Clinical Development and Publication of Trial Results. *JAMA Intern Med* 2016;**176**:1826–33. doi:10.1001/jamainternmed.2016.6008

148 Giri A, Hellwege JN, Keaton JM, *et al.* Trans-ethnic association study of blood pressure determinants in over 750,000 individuals. *Nat Genet* 2019;**51**:51–62. doi:10.1038/s41588-018-0303-9

149 Holmes M V, Simon T, Exeter HJ, *et al.* Secretory Phospholipase A_2-IIA and Cardiovascular Disease. *J Am Coll Cardiol* 2013;**62**:1966–76. doi:10.1016/j.jacc.2013.06.044

150 Rosenson RS, Hislop C, Elliott M, *et al.* Effects of varespladib methyl on biomarkers and major cardiovascular events in acute coronary syndrome patients. *J Am Coll Cardiol* 2010;**56**:1079–88. doi:10.1016/j.jacc.2010.06.015

151 Nicholls SJ, Kastelein JJP, Schwartz GG, *et al.* Varespladib and cardiovascular events in patients with an acute coronary syndrome: the VISTA-16 randomized clinical trial. *JAMA* 2014;**311**:252–62. doi:10.1001/jama.2013.282836

152 Liu Y, Beyer A, Aebersold R. On the Dependency of Cellular Protein Levels on mRNA Abundance. *Cell* 2016;**165**:535–50. doi:https://doi.org/10.1016/j.cell.2016.03.014

153 Verbanck M, Chen C-Y, Neale B, *et al.* Detection of widespread horizontal pleiotropy in causal relationships inferred from Mendelian randomization between complex traits and diseases. *Nat Genet* 2018;**50**:693–8. doi:10.1038/s41588-018-0099-7

154 Foulds CE. Disrupting a negative feedback loop drives endocrine therapy-resistant breast cancer. *Proc Natl Acad Sci* 2018;**115**:8236–8. doi:10.1073/pnas.1811263115

155 Cupido AJ, Asselbergs FW, Natarajan P, *et al.* Dissecting the IL-6 pathway in cardiometabolic disease: A Mendelian randomization study on both IL6 and IL6R. *Br J Clin Pharmacol*;**n/a**. doi:https://doi.org/10.1111/bcp.15191

156 Gallo MA, Kaufman D. Antagonistic and agonistic effects of tamoxifen: significance in human cancer. *Semin Oncol* 1997;**24**:S1—71—S1—80.http://europepmc.org/abstract/MED/9045319

157 Ference BA. How to use Mendelian randomization to anticipate the results of randomized trials. *Eur Heart J* 2017;**39**:360–2. doi:10.1093/eurheartj/ehx462

158 Holmes M V, Smith GD. Revealing the effect of CETP inhibition in cardiovascular disease. *Nat Rev Cardiol* 2017;**14**:635–6. doi:10.1038/nrcardio.2017.156

159 Kearns AE, Northfelt DW, Dueck AC, *et al.* Osteoporosis prevention in prostate cancer patients receiving androgen ablation therapy: placebo-controlled double-blind study of estradiol and risedronate: N01C8. *Support Care Cancer* 2010;**18**:321–8. doi:10.1007/s00520-009-0655-x

For peer review only - http://bmjopen.bmj.com/site/about/guidelines.xhtml

160 Li J, Guasch-Ferré M, Chung W, *et al.* The Mediterranean diet, plasma metabolome, and cardiovascular disease risk. *Eur Heart J* 2020;**41**:2645–56. doi:10.1093/eurheartj/ehaa209

161 Verfürden ML, Gilbert R, Lucas A, *et al.* Effect of nutritionally modified infant formula on academic performance: linkage of seven dormant randomised controlled trials to national education data. *BMJ* 2021;**375**:e065805. doi:10.1136/bmj-2021-065805

162 Paternoster L, Tilling K, Davey Smith G. Genetic epidemiology and Mendelian randomization for informing disease therapeutics: Conceptual and methodological challenges. *PLOS Genet* 2017;**13**:e1006944.https://doi.org/10.1371/journal.pgen.1006944

163 Stubbs B, Vancampfort D, Hallgren M, *et al.* EPA guidance on physical activity as a treatment for severe mental illness: a meta-review of the evidence and Position Statement from the European Psychiatric Association (EPA), supported by the International Organization of Physical Therapists in Mental . *Eur Psychiatry* 2018;**54**:124–44. doi:DOI: 10.1016/j.eurpsy.2018.07.004

164 Schooling CM, Freeman G, Cowling BJ. Mendelian Randomization and Estimation of Treatment Efficacy for Chronic Diseases. *Am J Epidemiol* 2013;**177**:1128–33. doi:10.1093/aje/kws344

165 Lor GCY, Risch HA, Fung WT, *et al.* Reporting and guidelines for mendelian randomization analysis: A systematic review of oncological studies. *Cancer Epidemiol* 2019;**62**:101577. doi:https://doi.org/10.1016/j.canep.2019.101577

166 Poole R, Kennedy OJ, Roderick P, *et al.* Coffee consumption and health: umbrella review of meta-analyses of multiple health outcomes. *BMJ* 2017;**359**. doi:10.1136/bmj.j5024

167 Altman DG, Bland JM. Statistics notes: Absence of evidence is not evidence of absence. *BMJ* 1995;**311**:485. doi:10.1136/bmj.311.7003.485

168 Sterne JAC, Smith GD. Sifting the evidence—what's wrong with significance tests? *Phys Ther* 2001;**81**:1464–9. doi:10.1093/ptj/81.8.1464

169 Lawlor DA, Tilling K, Davey Smith G. Triangulation in aetiological epidemiology. *Int J Epidemiol* 2016;**45**:1866–86. doi:10.1093/ije/dyw314

170 Nye BE, DeYoung J, Lehman E, *et al.* Understanding Clinical Trial Reports: Extracting Medical Entities and Their Relations. *AMIA Jt Summits Transl Sci proceedings AMIA Jt Summits Transl Sci* 2021;**2021**:485–94.https://pubmed.ncbi.nlm.nih.gov/34457164

171 Whitton J, Hunter A. Automated tabulation of clinical trial results: A joint entity and relation extraction approach with transformer-based language representations. 2021.

For peer review only - http://bmjopen.bmj.com/site/about/guidelines.xhtml

Figure legends

Figure 1. **Comparison of Mendelian Randomization and Randomized Controlled Trial design.** After: Nitsch *et al.* (2006), Ebrahim & Smith (2008), Ference (2018).

Figure 2. a) Filtering steps applied to ClinicalTrials.Gov database. Filtering was designed to identify RCT trials whose final results statistics were uploaded to the database (*Main dataset*). In addition, other RCTs which published their findings in scientific journals were identified (*Literature dataset*). **b) Filtering steps applied to EpigraphDB database.** Filtering was designed to identify protein QTL MR studies with intervention and exposure matching those of RCT published on ClinicalTrials.Gov.

Figure 3. Popularity of MR and RCT studies over time. We compare counts of MR and RCT papers indexed by PubMed (solid lines) with number of semantic triples derived from them using SemMedDb (dashed lines).

Figure 4. Summary of case series of MR and RCT studies with matching exposures (interventions) and outcomes (conditions). The values correspond to the number of analysed studies in a given category, while the cell background colour indicates summary direction of effect on the outcome when exposure is increased – we report direction of effect found either in all analysed studies or their majority (>50%).

For peer review only

For peer review only - http://bmjopen.bmj.com/site/about/guidelines.xhtml

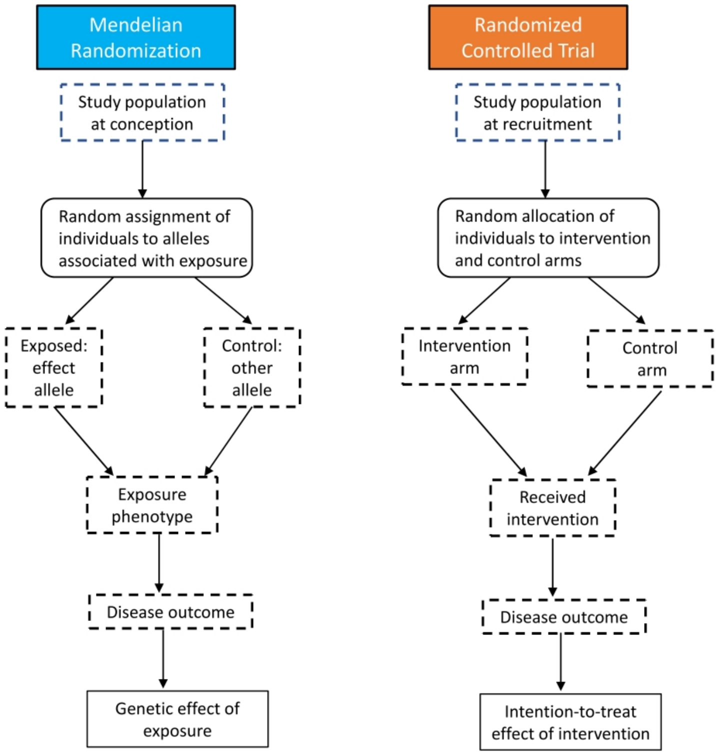

Comparison of Mendelian Randomization and Randomized Controlled Trial design. After: Nitsch et al. (2006), Ebrahim & Smith (2008), Ference (2018).

484x699mm (236 x 236 DPI)

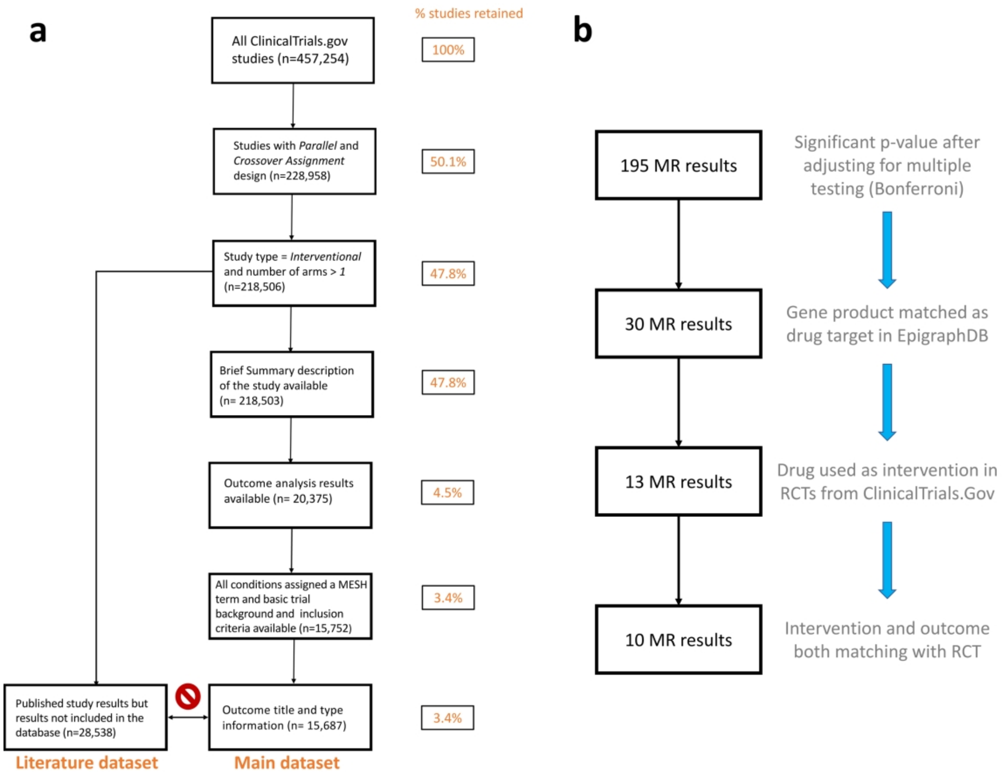

a) Filtering steps applied to ClinicalTrials.Gov database. Filtering was designed to identify RCT trials whose final results statistics were uploaded to the database (Main dataset). In addition, other RCTs which published their findings in scientific journals were identified (Literature dataset). b) Filtering steps applied to EpigraphDB database. Filtering was designed to identify protein QTL MR studies with intervention and exposure matching those of RCT published on ClinicalTrials.Gov.

268x207mm (300 x 300 DPI)

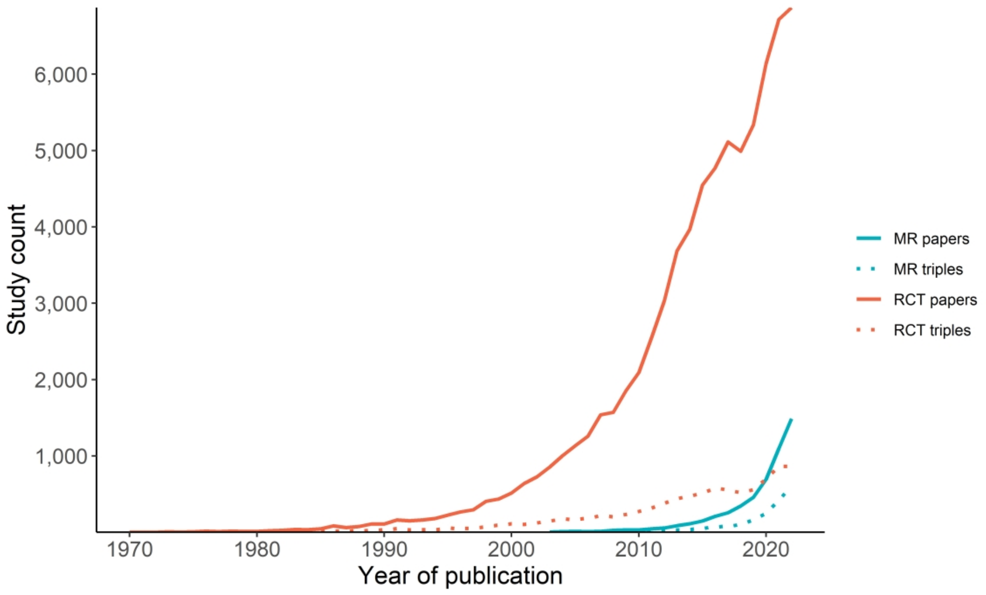

Popularity of MR and RCT studies over time. We compare counts of MR and RCT papers indexed by PubMed (solid lines) with number of semantic triples derived from them using SemMedDb (dashed lines).

508x305mm (236 x 236 DPI)

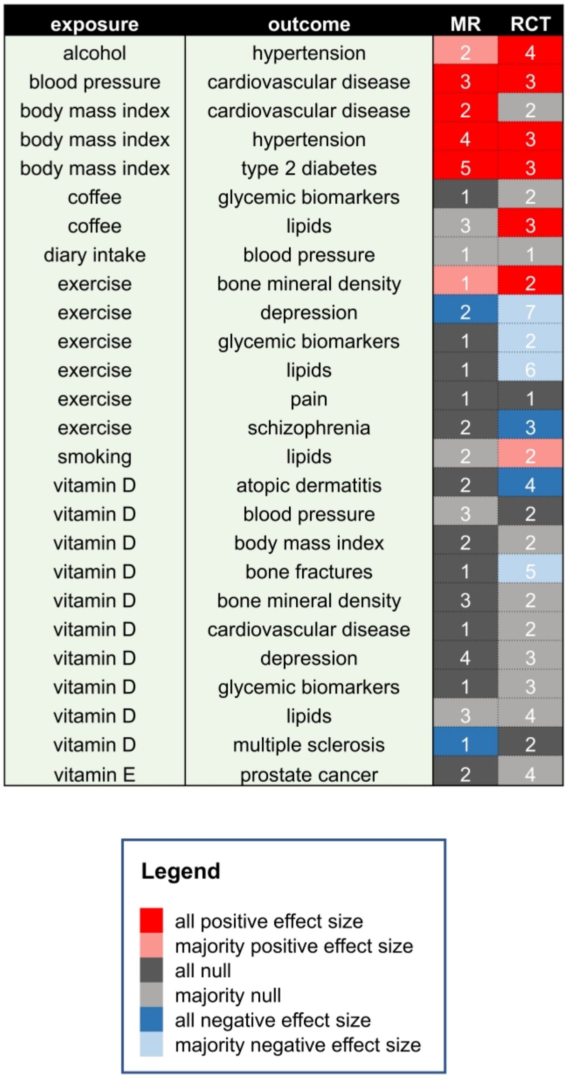

exposure	outcome	MR	RCT
alcohol	hypertension	2	4
blood pressure	cardiovascular disease	3	3
body mass index	cardiovascular disease	2	2
body mass index	hypertension	4	3
body mass index	type 2 diabetes	5	3
coffee	glycemic biomarkers	1	2
coffee	lipids	3	3
diary intake	blood pressure	1	1
exercise	bone mineral density	1	2
exercise	depression	2	7
exercise	glycemic biomarkers	1	2
exercise	lipids	1	6
exercise	pain	1	1
exercise	schizophrenia	2	3
smoking	lipids	2	2
vitamin D	atopic dermatitis	2	4
vitamin D	blood pressure	3	2
vitamin D	body mass index	2	2
vitamin D	bone fractures	1	5
vitamin D	bone mineral density	3	2
vitamin D	cardiovascular disease	1	2
vitamin D	depression	4	3
vitamin D	glycemic biomarkers	1	3
vitamin D	lipids	3	4
vitamin D	multiple sclerosis	1	2
vitamin E	prostate cancer	2	4

Summary of case series of MR and RCT studies with matching exposures (interventions) and outcomes (conditions). The values correspond to the number of analysed studies in a given category, while the cell background colour indicates summary direction of effect on the outcome when exposure is increased – we report direction of effect found either in all analysed studies or their majority (>50%).

484x861mm (236 x 236 DPI)

Supplementary Box

It is anticipated that the longer duration of exposure differences seen in MR studies will generate larger effect sizes than will relatively short-term modification of the exposure in RCTs. For example, the genetic variants related to non-HDL cholesterol (henceforth "cholesterol", the target of cholesterol lowering drugs such as the statins) have been shown to relate to relatively stable differences from early childhood to late adulthood, thus generating a lifetime of differential exposure to circulating cholesterol. The RCTs of cholesterol lowering drugs designed to show effects on coronary heart disease (CHD) events last ~5 years. Atherosclerosis is a disease process that develops from childhood onwards, and the CHD it generates would not be expected to be abolished by a few years of cholesterol lowering in middle age or older (the usual age included in the RCTs). As there are several cholesterol lowering drugs which target different genes it is possible to compare MR studies using genetic variants in those genes that are robustly related to cholesterol with RCTs of drugs that target those genes. Figure SB1 below combines data from such MR analyses with the RCTs.

As anticipated the RCTs produce about 40% of the risk reduction seen with a lifetime difference in exposure levels[1]. This scaling of the effects predicted from MR studies and seen in the matching trials can be applied to MR/RCT comparisons for other exposures, as the time course of effects being produced may be quicker than seen in the case of cholesterol, or take longer, or indeed there may be no effect in the RCTs if the effect of the exposure acts during a critical period in earlier life and sets in train a disease process that is not reversible by later modification of the exposure[2].

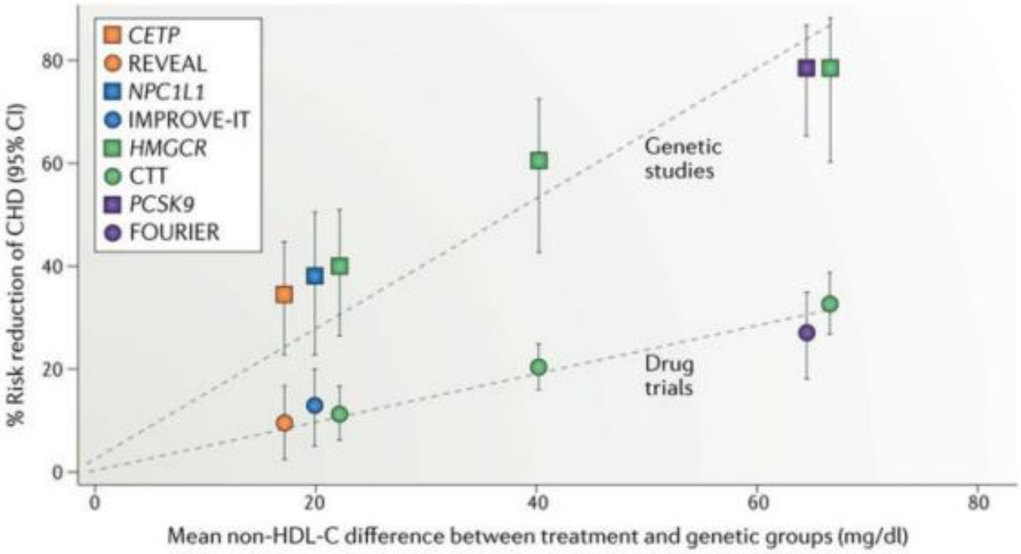

Figure SB1. Drug treatment (circles) and genetic proxy (squares) effects on reducing cholesterol levels and the corresponding reduction in risk of from matching drug RCTs and MR analyses. The colours indicate the gene from which variants are taken in the MR studies and the target of the drug used in the named RCTs. *Abbreviations: CETP, cholesteryl ester transfer protein; HMGCR, 3-hydroxy-3-methylglutaryl-CoA reductase; NPC1L1, Niemann-Pick C1-like protein 1; PCSK9, proprotein convertase subtilisin/kexin type 9.* Figure reproduced with permission from[3].

References

1. Smith GD, Ebrahim S. Mendelian randomization: prospects, potentials, and limitations. Int J Epidemiol. 2004;33: 30–42. doi:10.1093/ije/dyh132

2. Holmes M V, Ala-Korpela M, Smith GD. Mendelian randomization in cardiometabolic disease: challenges in evaluating causality. Nat Rev Cardiol. 2017;14: 577–590. doi:10.1038/nrcardio.2017.78

3. Holmes M V, Smith GD. Revealing the effect of CETP inhibition in cardiovascular disease. Nat Rev Cardiol. 2017;14: 635–636. doi:10.1038/nrcardio.2017.156

For peer review only

Supplementary Note

ClinicalTrials.Gov *Data filtering*

We only included studies with most common designs suitable for comparison with published MR studies: *Parallel Assignment* and *Crossover Assignment* (eliminating: *Single Group Assignment, Sequential Assignment, Factorial Assignment*) intervention model in the Designs table with *Randomized* allocation to allow for selection of RCT. We further used the study type field (=*Interventional*) with a minimum number of arms = *2* in the Studies table as an additional filtering criterion to arrive at a set of RCT studies. The next stage of filtering concerned background information and study results. We first filtered on the presence of a study description in the Brief Summaries table. The key criterion was then presence of results in the Outcome Analyses table, where we selected the variables: *param_type, param_value, p_value* and *method.* Next, we needed all conditions to have at least 1 Medical Subject Headings (MeSH) term assigned in all_conditions view to facilitate automatic comparison with external data sources. We dropped that requirement for interventions, as especially behavioural interventions could not be assigned a MeSH term (see Results). Finally, a range of basic reference and eligibility criteria were required: *brief_title, study_type, overall_status, phase, number_of_arms, enrolment* in the Studies table, *gender, criteria* in the Eligibilities table and *outcome title* and *type* in the Outcomes table.

Additionally, we extracted a subset of studies which did not supply any results in the Outcome Analyses table and therefore did not contribute to the *Main* dataset above but were RCT studies with published literature records in the Study References table (*reference type* = result).

Overview of top SemMed triples for MR and RCT studies

An overview of the top subjects in MR (Figure S4a) and RCTs (Figure S4b) revealed a high number of terms related to adiposity (*obesity, body mass index, adiponectin*), lipid biology (*lipoproteins, hydroxymethylglutaryl-CoA reductase inhibitors, PCKSK9, high density lipoproteins*) as well as *type 2 diabetes* and *vitamin D,* and recently *COVID-19*. Terms related to type 2 diabetes (*insulin, metformin, diabetes*) and *vitamin D* were also found in RCT triples. As expected by the preponderance of drug interventions in ClinicalTrials.Gov, there was a noticeable bias towards pharmaceutical preparations amongst the top 10 subjects in RCT triples with terms such as *methotrexate*, *aspirin*, *clomiphene citrate*, *dexamethasone*, *prednisolone* and *cyclosporine.* SemMedDB identified *associated with* as a top predicate in MR studies (Figure S4c), followed by *predisposes*, *coexists with* and *affects* which underlines the skew of current MR studies towards identifying risk factors for disease. On the other hand, RCT studies lean towards identifying treatments (Figure S4d) which is reflected in the top 1 predicate *treats* (*n*=7,791, second-best *coexists with* n=2,149). Among the top objects in MR studies (Figure S4e), we found cardiovascular diseases (*coronary arteriosclerosis*, *coronary heart disease*, *myocardial ischemia/infarction*, *hypertensive disease*, *ischemic stroke*). High frequency of a smaller number of cardiovascular disease terms (Figure S4f) was also found among RCT objects (*hypertensive disease, cardiovascular disease*). Both MR and

For peer review only

RCT showed type 2 diabetes, obesity, Alzheimer's disease and COVID-19 as commonly studied conditions. RCT objects included also two infectious diseases: malaria and hepatitis C.

For peer review only

Supplementary Figures

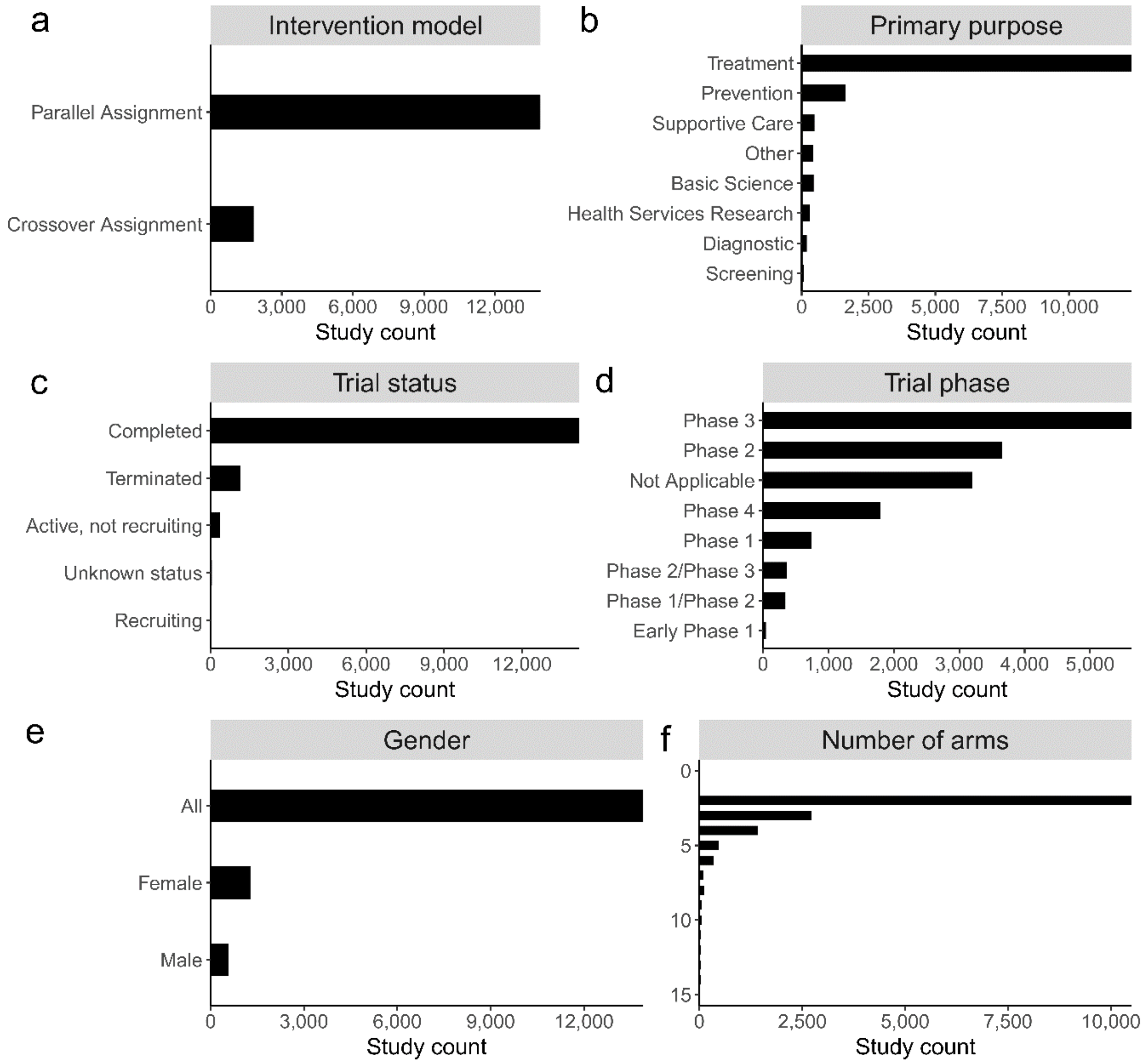

Figure S1. Overview of RCT general features in the Main dataset by their frequency: a) intervention model, b) primary purpose, c) trial status, d) trial phase, e) gender, f) number of arms.

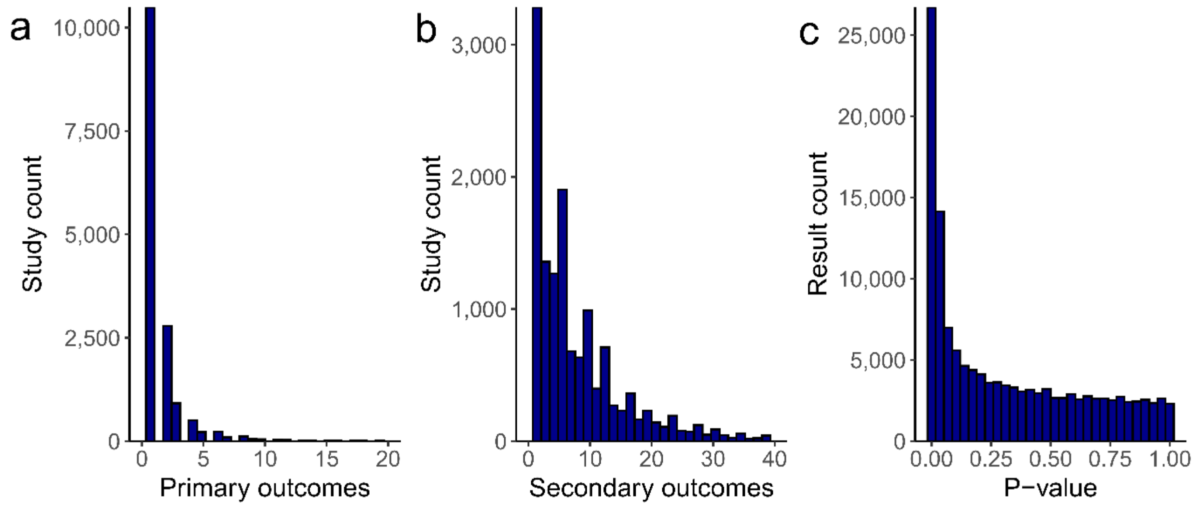

Figure S2. Distribution of: a) primary outcome number among RCT studies, b) secondary outcome number among RCT studies, c) P-value among study results.

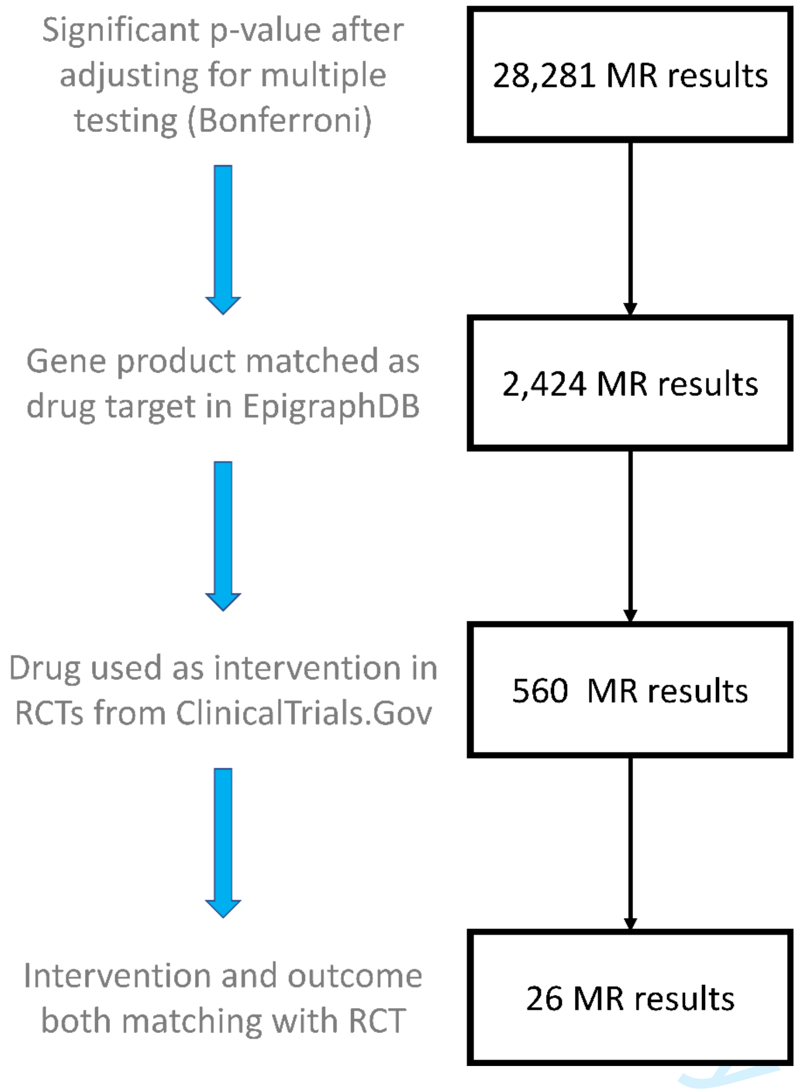

Figure S3. Filtering steps applied to EpigraphDB database. Filtering was designed to identify expression QTL MR studies with intervention and exposure matching those of RCT published on ClinicalTrials.Gov.

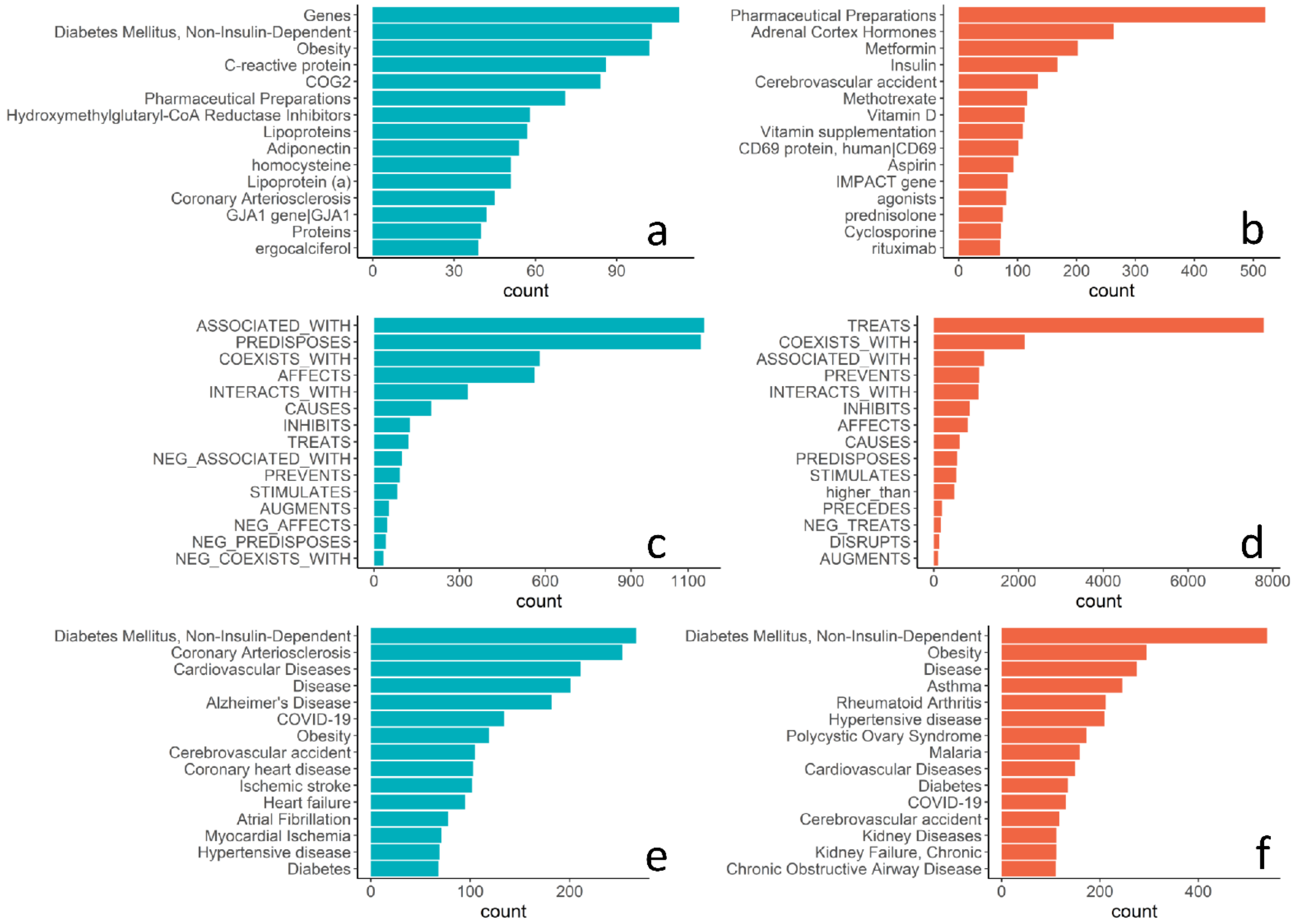

Figure S4. Overview of SemMedDb semantic triples derived from MR (blue, left-hand panels) and RCT (orange, right-hand panels) studies: a) & b) top 15 subjects, c) & d) top 15 predicates, d) & e) top 15 objects.

Supplementary Tables legends

Table S1. Comparison of the most common intervention types in the *Main* and *Literature* datasets.

Table S2. Drug target-disease matches supported by evidence from MR (blood eQTL instruments, https://epigraphdb.org/xqtl) and RCT studies (*Main dataset* from ClinicalTrials.Gov).

Table S3. Matching subject-object pairs between MR and RCT studies in semantic triples identified by SemMedDb.

Table S4. Matching subjects between MR and RCT studies in semantic triples identified by SemMedDb.

Table S5. Matching objects between MR and RCT studies in semantic triples identified by SemMedDb.

Table S6. Sample comparison of MR and RCT studies with matching exposures and outcomes across a range of criteria.

Supplementary Dataset 1. Select ClinicalTrials.Gov data fields for the RCT studies identified as the *main dataset*.

Supplementary Dataset 2. Select ClinicalTrials.Gov data fields for the RCT studies identified as the *literature dataset*.

Supplementary Dataset 3. Comparison of frequency of RCT general features between the *Main dataset* and background of all RCTs available in ClinicalTrials.Gov.

Supplementary Dataset 4. PubMed and SemMedDB data for published MR and RCT studies.

Supplementary Dataset 5. Case series of MR and RCT studies with matching exposures (interventions) and outcomes (conditions).

For peer review only

Table S1

Intervention	Main	Main %	Literature	Literature %
Drug	11,537	73.5%	10,927	38.3%
Other	2,054	13.1%	5,005	17.5%
Biological	1,497	9.5%	1,665	5.8%
Behavioral	1,239	7.9%	3,017	10.6%
Device	1,427	9.1%	3,242	11.4%
Procedure	481	3.1%	3,597	12.6%
Dietary Supplement	242	1.5%	830	2.9%
Radiation	134	0.9%	764	2.7%
Genetic	21	0.1%	145	0.5%
Combination Product	42	0.3%	135	0.5%
Diagnostic Test	15	0.1%	842	3.0%

For peer review only

Table S2

MR Exposure	RCT Drug Intervention	MR Outcomes	RCT Conditions	Matching trials	Concordant direction of effect?	xQTL
CETP	EVACETRAPIB, ANACETRAPIB	LDL cholesterol || id:300, HDL cholesterol || id:299, Total cholesterol || id:301, Triglycerides || id:302	Hyperlipidemia, Dyslipidemia, Hypercholesterolemia, Hyperlipoproteinemia Type II	7	Yes	eQTL
HMGCR	ATORVASTATIN, PRAVASTATIN, SIMVASTATIN	LDL cholesterol || id:300, Total cholesterol || id:301, Non-cancer illness code, self-reported: high cholesterol || id:UKB-b:10912	Hyperlipidemia, Dyslipidemia, Hypercholesterolemia, Mixed Dyslipidemia etc.	50	Yes	eQTL
GUCY1A3 and GUCY1B3	RIOCIGUAT	Diastolic blood pressure automated reading || id:UKB-a:359, Vascular/heart problems diagnosed by doctor: High blood pressure || id:UKB-b:14177	Pulmonary Hypertension, Pulmonary Arterial Hypertension	7	Yes	eQTL
IGF1R	MECASERMIN	Height || id:89	Growth Disorders|Insulin-Like Growth Factor-1 Deficiency	1	No	eQTL
IL2RA	DACLIZUMAB	Type 1 diabetes || id:285	Diabetes Mellitus, Type 1	1	No - no significant effect seen in RCT	eQTL
KCNJ11	GLIMEPIRIDE	Diabetes diagnosed by doctor || id:UKB-b:10753	Diabetes Mellitus, Type 2, Diabetes, Pre-diabetes	63	Yes	eQTL

For peer review only

ACE	ENALAPRIL, LISINOPRIL, PERINDOPRIL	Vascular/heart problems diagnosed by doctor: High blood pressure || id:UKB-b:14177	Hypertension, Essential Hypertension, Hypertension, Pulmonary|Ventricular Dysfunction, Left	3	Yes	eQTL
PDE3A	ANAGRELIDE	Platelet count || id:1008	Thrombocythemia	1	Yes	eQTL
CD86	ABATACEPT	Multiple sclerosis || id:1025	Multiple Sclerosis, Relapsing-Remitting	1	No - no significant effect seen in RCT	eQTL
ESR1	FULVESTRANT, TAMOXIFEN	Breast cancer (Combined Oncoarray; iCOGS; GWAS meta analysis) || id:1126	Breast Cancer, Estrogen Receptor-positive (ER+) Breast Cancer etc.	9	Yes*	eQTL
ESR1	ESTRADIOL, POLYESTRADIOL PHOSPHATE	Femoral neck bone mineral density || id:980, Lumbar spine bone mineral density || id:982, Heel bone mineral density (BMD) T-score automated || id:UKB-a:500	Bone Mineral Density	1	No*	eQTL
PARP3	OLAPARIB, VELIPARIB, RUCAPARIB	Ovarian cancer || id:1120	Ovarian Neoplasms, Ovarian Cancer etc.	7	No	eQTL

For peer review only

MS4A1	OCRELIZUMAB, OFATUMUMAB, RITUXIMAB	Rheumatoid arthritis || id:833, Type 1 Diabetes Mellitus	Rheumatoid Arthritis, Type 1 Diabetes Mellitus	10	No	eQTL
JAK2	TOFACITINIB	Crohn's disease || id:12, Inflammatory bowel disease || id:294	Ulcerative Colitis, Crohn's Disease	6	No	eQTL
TYK2	TOFACITINIB, PEFICITINIB, UPADACITINIB	Rheumatoid arthritis || id:833, Psoriasis || id:282	Rheumatoid arthritis, Psoriasis	34	No	eQTL

For peer review only

Table S3

subject	object	MR	RCT
Obesity	Disease	9	5
Lipoproteins	Cardiovascular Diseases	8	1
Diabetes Mellitus, Non-Insulin-Dependent	Disease	7	2
Obesity	Diabetes Mellitus, Non-Insulin-Dependent	7	6
Asthma	Obesity	6	1
Insulin	Obesity	6	8
Adiponectin	Diabetes Mellitus, Non-Insulin-Dependent	5	1
Coronary Arteriosclerosis	Cardiovascular Diseases	5	1
COVID-19	Disease	5	1
Diabetes Mellitus, Non-Insulin-Dependent	Diabetes	5	5
Disease	Diabetes Mellitus, Non-Insulin-Dependent	5	2
Myocardial Infarction	Cardiovascular Diseases	5	1
Obesity	Asthma	5	4
Pharmaceutical Preparations	Disease	5	14
C-reactive protein	Cardiovascular Diseases	4	1
Cerebrovascular accident	Cardiovascular Diseases	4	1
cytokine	Disease	4	1
ergocalciferol	COVID-19	4	1
Hypertensive disease	Cardiovascular Diseases	4	18
Insulin	Diabetes Mellitus, Non-Insulin-Dependent	4	36
Lipids	Pharmaceutical Preparations	4	1
Lipoproteins	Coronary heart disease	4	1
Pharmaceutical Preparations	Coronary Arteriosclerosis	4	4
Amino Acids, Branched-Chain	Diabetes Mellitus, Non-Insulin-Dependent	3	1
C-reactive protein	Diabetes Mellitus, Non-Insulin-Dependent	3	1
CD69 protein, human|CD69	Cerebrovascular accident	3	1
Diabetes Mellitus, Non-Insulin-Dependent	Cardiovascular Diseases	3	10
Genes	Pharmaceutical Preparations	3	1
High Density Lipoproteins	Coronary Arteriosclerosis	3	1
Hydroxymethylglutaryl-CoA Reductase Inhibitors	Coronary Arteriosclerosis	3	2
Hydroxymethylglutaryl-CoA Reductase Inhibitors	HMGCR gene|HMGCR	3	1
IMPACT gene	Cardiovascular Diseases	3	2
mullerian-inhibiting hormone	Polycystic Ovary Syndrome	3	4
Obesity	Cardiovascular Diseases	3	7
Obesity	COVID-19	3	2
Obesity	Hypertensive disease	3	3
Pharmaceutical Preparations	Cardiovascular Diseases	3	1
Polycystic Ovary Syndrome	Disease	3	1
Vitamin D Deficiency	Cardiovascular Diseases	3	1
Vitamin D	Disease	3	1
Apolipoprotein E|APOE	Alzheimer's Disease	2	2
Atrial Fibrillation	Ischemic stroke	2	1
Cardiovascular Diseases	Diabetes Mellitus, Non-Insulin-Dependent	2	3
Cardiovascular Diseases	Disease	2	1
Coronary Arteriosclerosis	Disease	2	1
Coronary Arteriosclerosis	Heart failure	2	2
COVID-19	Obesity	2	2

For peer review only

Diabetes Mellitus, Non-Insulin-Dependent	COVID-19	2	1
Diabetes Mellitus, Non-Insulin-Dependent	Heart failure	2	1
Diabetic Nephropathy	Diabetes Mellitus, Insulin-Dependent	2	1
Disease	Cerebrovascular accident	2	1
Disease	Multiple Sclerosis	2	1
Disease	Obesity	2	2
Dyslipidemias	Coronary Arteriosclerosis	2	1
ergocalciferol	Cardiovascular Diseases	2	1
ergocalciferol	Disease	2	1
Ethanol	Disease	2	2
GJA1 gene|GJA1	Obesity	2	1
Heart failure	Ischemic stroke	2	1
homocysteine	Alzheimer's Disease	2	2
homocysteine	Cardiovascular Diseases	2	6
Hyperandrogenism	Polycystic Ovary Syndrome	2	5
Hypertensive disease	Cerebrovascular accident	2	4
IMPACT gene	COVID-19	2	2
Myocardial Infarction	Heart failure	2	2
Obesity	Polycystic Ovary Syndrome	2	1
Obesity	Psoriasis	2	1
Pharmaceutical Preparations	Alzheimer's Disease	2	5
Pharmaceutical Preparations	Atherosclerosis	2	1
Pharmaceutical Preparations	Diabetes	2	14
Pharmaceutical Preparations	Parkinson Disease	2	1
Testosterone	Risk factor, cardiovascular	2	1
Vitamin B 12	Body mass index	2	1
Vitamin D	Vitamin D Deficiency	2	3
Vitamin supplementation	Cardiovascular Diseases	2	3
Vitamin supplementation	COVID-19	2	1
Vitamin supplementation	Diabetes Mellitus, Non-Insulin-Dependent	2	3
Vitamin supplementation	Vitamin D Deficiency	2	3
acylcarnitine	Fatty Acids	1	1
Adipokines	Diabetes Mellitus, Non-Insulin-Dependent	1	1
Adipokines	Obesity	1	2
Adiponectin	Obesity	1	1
Age related macular degeneration	Blind Vision	1	2
Alanine Transaminase	Alkaline Phosphatase	1	1
Analgesics	Degenerative polyarthritis	1	4
Angiotensin-Converting Enzyme Inhibitors	Angiotensin Receptor	1	2
Anti-Inflammatory Agents	Disease	1	2
Anticoagulants	Myocardial Ischemia	1	1
Antioxidants	Coronary Arteriosclerosis	1	1
Antioxidants	Rheumatoid Arthritis	1	1
Apolipoproteins B	Apolipoprotein A-I	1	1
Apolipoproteins	Alzheimer's Disease	1	1
ascorbic acid	COVID-19	1	1
Atherosclerosis	Cardiovascular Diseases	1	1
Atherosclerosis	Diabetes Mellitus, Non-Insulin-Dependent	1	1
Atrial Fibrillation	Sleep Apnea, Obstructive	1	1
bempedoic acid	Hypercholesterolemia	1	1

For peer review only

Beta Carotene	Diabetes Mellitus, Non-Insulin-Dependent	1	1
Biological Markers	Alzheimer's Disease	1	1
Body mass index	Diabetes	1	1
C-reactive protein	Disease	1	1
C-reactive protein	Obesity	1	3
Cardiovascular Diseases	Coronary heart disease	1	1
Cardiovascular Diseases	Polycystic Ovary Syndrome	1	1
Carotid Intima-Media Thickness	Arteriosclerosis	1	1
CD69 protein, human|CD69	Alzheimer's Disease	1	1
CD69 protein, human|CD69	Cardiovascular Diseases	1	1
CD69 protein, human|CD69	Hypertensive disease	1	1
CD69 protein, human|CD69	Obesity	1	2
Cerebrovascular accident	Carotid Atherosclerosis	1	1
Cerebrovascular accident	Disease	1	3
Cerebrovascular accident	Ischemic stroke	1	1
Cerebrovascular accident	Myocardial Infarction	1	1
Cigarette smoke (substance)	Tobacco	1	1
coenzyme Q10	Disease	1	1
Complement System Proteins	Disease	1	1
Coronary Arteriosclerosis	Periodontitis	1	1
Coronary heart disease	Obesity	1	1
COVID-19	Critical Illness	1	1
COVID-19	Respiratory Distress Syndrome, Adult	1	2
Crohn's disease	Disease	1	1
cytokine	Rheumatoid Arthritis	1	1
DEFA1A3	Periodontitis	1	1
dexamethasone	COVID-19	1	1
Diabetes Mellitus, Non-Insulin-Dependent	Alzheimer's Disease	1	1
Diabetes Mellitus, Non-Insulin-Dependent	Sleep Apnea, Obstructive	1	2
Diabetes	Obesity	1	4
Diabetes	Polycystic Ovary Syndrome	1	1
Disease	Fatty Liver	1	1
Disease	Liver diseases	1	1
Disease	Lupus Erythematosus, Systemic	1	1
Dyslipidemias	Cardiovascular Diseases	1	2
Enzymes	Vitamin D	1	1
ergocalciferol	calcifediol	1	2
ergocalciferol	Hypertensive disease	1	1
ergocalciferol	Vitamin D Deficiency	1	3
Ethanol	Cardiovascular Diseases	1	1
Folate	homocysteine	1	4
Free thyroxin	Thyrotropin	1	1
Galectin 3	Heart failure	1	1
Gastroesophageal reflux disease	Asthma	1	1
Genes	Dyslipidemias	1	1
Genes	Hypertensive disease	1	1
GJA1 gene|GJA1	Asthma	1	1
GJA1 gene|GJA1	Hypertensive disease	1	1
Glaucoma	Disease	1	1
Glycosylated hemoglobin A	Diabetes Mellitus, Non-Insulin-Dependent	1	2

For peer review only

Hemoglobin	Anemia	1	2
homocysteine	Cerebrovascular accident	1	1
Hydroxymethylglutaryl-CoA Reductase Inhibitors	Atherosclerosis	1	1
Hydroxymethylglutaryl-CoA Reductase Inhibitors	COG2	1	2
Hydroxymethylglutaryl-CoA Reductase Inhibitors	Coronary heart disease	1	1
Hydroxymethylglutaryl-CoA Reductase Inhibitors	Dyslipidemias	1	4
Hyperglycemia	Diabetes Mellitus, Non-Insulin-Dependent	1	1
Hyperlipidemia	Diabetes	1	1
Hyperlipidemia	Disease	1	1
Hypertensive disease	Cardiovascular morbidity	1	3
Hypertensive disease	Myocardial Ischemia	1	1
Hypertensive disease	Obesity	1	4
Hypertensive disease	Sleep Apnea, Obstructive	1	4
Hyperuricemia	Gout	1	1
icosapent ethyl	Hypertriglyceridemia	1	1
IGHE	Disease	1	3
IMPACT gene	Cerebrovascular accident	1	2
IMPACT gene	Disease	1	3
IMPACT gene	Non-alcoholic Fatty Liver Disease	1	1
Inflammatory Bowel Diseases	Crohn's disease	1	1
Insulin-Like Growth Factor Binding Protein 3	Insulin-Like Growth Factor I	1	1
Insulin	Heart failure	1	1
Insulin	Myocardial Infarction	1	1
Insulin	Pancreatitis	1	1
Interleukin-6	Hypertensive disease	1	1
Ischemic stroke	Atrial Fibrillation	1	1
LOC107984137	Periodontitis	1	1
Low-Density Lipoproteins	High Density Lipoproteins	1	1
Malnutrition	Disease	1	1
Metabolic syndrome	Diabetes	1	2
Metabolic syndrome	Obesity	1	3
Metformin	Diabetes	1	6
MTND1P5	Periodontitis	1	1
Myocardial Infarction	Venous Thromboembolism	1	1
Nitrous Oxide	Vitamin B 12	1	1
Non-alcoholic Fatty Liver Disease	Obesity	1	2
Non-alcoholic fatty liver	Diabetes Mellitus, Non-Insulin-Dependent	1	2
Non-alcoholic fatty liver	Fatty Liver	1	1
Obesity	Chronic Disease	1	3
Obesity	Diabetes	1	3
Obesity	Metabolic Diseases	1	1
Periodontitis	Cardiovascular Diseases	1	2
Pharmaceutical Preparations	Asthma	1	18
Pharmaceutical Preparations	Cerebrovascular accident	1	4
Pharmaceutical Preparations	COVID-19	1	2
Pharmaceutical Preparations	Heart failure	1	3
Pharmaceutical Preparations	Hypertensive disease	1	26
Pharmaceutical Preparations	Ischemic stroke	1	1
Pharmaceutical Preparations	Rheumatoid Arthritis	1	5
Proteins	Diabetes	1	1

For peer review only

Rheumatoid Arthritis	Osteoporosis	1	1
Risk factor, cardiovascular	Atherosclerosis	1	1
Risk factor, cardiovascular	Diabetes Mellitus, Non-Insulin-Dependent	1	5
Risk factor, cardiovascular	Hypertensive disease	1	2
Risk factor, cardiovascular	Obesity	1	2
SIGLEC5	Periodontitis	1	1
STN gene|EEF1A2	COG2	1	3
Testosterone	Obesity	1	1
Testosterone	Vitamin D	1	1
tocilizumab	Rheumatoid Arthritis	1	4
Virus Diseases	Common Cold	1	2
Vitamin B 12	homocysteine	1	3
vitamin B12	Vitamin B 12 Deficiency	1	1
Vitamin D	Adiponectin	1	1
Vitamin D	Alzheimer's Disease	1	1
Vitamin D	Asthma	1	1
Vitamin D	Cardiovascular Diseases	1	2
Vitamin D	Diabetes Mellitus, Non-Insulin-Dependent	1	2
Vitamin D	Genes	1	1
Vitamin D	Risk factor, cardiovascular	1	1
Vitamin E	Disease	1	3
Vitamin supplementation	Alzheimer's Disease	1	1
Vitamins	homocysteine	1	1
zinc	COVID-19	1	1

For peer review only

Table S4

subject	MR	RCT
Obesity	36	33
Pharmaceutical Preparations	26	92
Diabetes Mellitus, Non-Insulin-Dependent	20	21
Insulin	13	45
Disease	13	9
Lipoproteins	12	2
Vitamin D	11	13
ergocalciferol	11	8
Hypertensive disease	10	33
Hydroxymethylglutaryl-CoA Reductase Inhibitors	10	10
Coronary Arteriosclerosis	10	5
C-reactive protein	9	6
COVID-19	9	6
Vitamin supplementation	8	11
IMPACT gene	8	10
Cerebrovascular accident	8	7
Myocardial Infarction	7	4
CD69 protein, human|CD69	6	6
Adiponectin	6	2
Asthma	6	1
homocysteine	5	9
Cardiovascular Diseases	5	6
Genes	5	3
cytokine	5	2
GJA1 gene|GJA1	4	3
Testosterone	4	3
Lipids	4	1
mullerian-inhibiting hormone	3	4
Vitamin B 12	3	4
Dyslipidemias	3	3
Ethanol	3	3
Atrial Fibrillation	3	2
Amino Acids, Branched-Chain	3	1
High Density Lipoproteins	3	1
Polycystic Ovary Syndrome	3	1
Vitamin D Deficiency	3	1
Risk factor, cardiovascular	2	10
Diabetes	2	5
Hyperandrogenism	2	5
Metabolic syndrome	2	5
Non-alcoholic fatty liver	2	3
Antioxidants	2	2
Apolipoprotein E|APOE	2	2
Atherosclerosis	2	2
Hyperlipidemia	2	2
Diabetic Nephropathy	2	1
Heart failure	2	1

For peer review only

Metformin	1	6
Analgesics	1	4
Folate	1	4
tocilizumab	1	4
IGHE	1	3
STN gene|EEF1A2	1	3
Vitamin E	1	3
Adipokines	1	2
Age related macular degeneration	1	2
Angiotensin-Converting Enzyme Inhibitors	1	2
Anti-Inflammatory Agents	1	2
Glycosylated hemoglobin A	1	2
Hemoglobin	1	2
Non-alcoholic Fatty Liver Disease	1	2
Periodontitis	1	2
Virus Diseases	1	2
acylcarnitine	1	1
Alanine Transaminase	1	1
Anticoagulants	1	1
Apolipoproteins	1	1
Apolipoproteins B	1	1
ascorbic acid	1	1
bempedoic acid	1	1
Beta Carotene	1	1
Biological Markers	1	1
Body mass index	1	1
Carotid Intima-Media Thickness	1	1
Cigarette smoke (substance)	1	1
coenzyme Q10	1	1
Complement System Proteins	1	1
Coronary heart disease	1	1
Crohn's disease	1	1
DEFA1A3	1	1
dexamethasone	1	1
Enzymes	1	1
Free thyroxin	1	1
Galectin 3	1	1
Gastroesophageal reflux disease	1	1
Glaucoma	1	1
Hyperglycemia	1	1
Hyperuricemia	1	1
icosapent ethyl	1	1
Inflammatory Bowel Diseases	1	1
Insulin-Like Growth Factor Binding Protein 3	1	1
Interleukin-6	1	1
Ischemic stroke	1	1
LOC107984137	1	1
Low-Density Lipoproteins	1	1
Malnutrition	1	1
MTND1P5	1	1

For peer review only

Nitrous Oxide	1	1
Proteins	1	1
Rheumatoid Arthritis	1	1
SIGLEC5	1	1
vitamin B12	1	1
Vitamins	1	1
zinc	1	1

For peer review only

Table S5

object	MR	RCT
Disease	50	51
Cardiovascular Diseases	43	62
Diabetes Mellitus, Non-Insulin-Dependent	37	63
Obesity	29	36
COVID-19	16	12
Alzheimer's Disease	12	14
Coronary Arteriosclerosis	12	9
Cerebrovascular accident	10	13
Diabetes	10	33
Hypertensive disease	10	36
Asthma	8	25
Heart failure	8	9
Polycystic Ovary Syndrome	8	10
Pharmaceutical Preparations	7	2
Coronary heart disease	6	3
Ischemic stroke	6	4
Vitamin D Deficiency	5	9
Atherosclerosis	4	3
Rheumatoid Arthritis	4	11
HMGCR gene|HMGCR	3	1
Risk factor, cardiovascular	3	2
Body mass index	2	1
COG2	2	5
Diabetes Mellitus, Insulin-Dependent	2	1
Dyslipidemias	2	5
homocysteine	2	8
Multiple Sclerosis	2	1
Myocardial Infarction	2	2
Myocardial Ischemia	2	2
Parkinson Disease	2	1
Periodontitis	2	2
Psoriasis	2	1
Sleep Apnea, Obstructive	2	7
Vitamin D	2	2
Adiponectin	1	1
Alkaline Phosphatase	1	1
Anemia	1	2
Angiotensin Receptor	1	2
Apolipoprotein A-I	1	1
Arteriosclerosis	1	1
Atrial Fibrillation	1	1
Blind Vision	1	2
calcifediol	1	2
Cardiovascular morbidity	1	3
Carotid Atherosclerosis	1	1
Chronic Disease	1	3
Common Cold	1	2

For peer review only

Critical Illness	1	1
Crohn's disease	1	1
Degenerative polyarthritis	1	4
Fatty Acids	1	1
Fatty Liver	1	1
Genes	1	1
Gout	1	1
High Density Lipoproteins	1	1
Hypercholesterolemia	1	1
Hypertriglyceridemia	1	1
Insulin-Like Growth Factor I	1	1
Liver diseases	1	1
Lupus Erythematosus, Systemic	1	1
Metabolic Diseases	1	1
Non-alcoholic Fatty Liver Disease	1	1
Osteoporosis	1	1
Pancreatitis	1	1
Respiratory Distress Syndrome, Adult	1	2
Thyrotropin	1	1
Tobacco	1	1
Venous Thromboembolism	1	1
Vitamin B 12	1	1
Vitamin B 12 Deficiency	1	1

For peer review only

Table S6

	MR	RCT	
	Mokry 2015	James 2013	Hempel 2017
**Exposure:**	vitamin D	vitamin D	vitamin D
**Outcome(s):**	multiple sclerosis	multiple sclerosis relapse	multiple sclerosis as measured with EDSS score
**Population (Sex, ethnicity and age):**	mixed, European	mixed, European	mixed, European
**Population (health status):**	all	meta-analysis of 5 RCTs	meta-analysis of 5 RCTs
**Comparator group:**	NA	low dose vitamin D, placebo	low dose vitamin D, placebo
**Prevention or Treatment:**	prevention	treatment	treatment
**Direction of effect:**	negative	null	null
**Intervention duration:**	lifetime	range: 6 months-96 weeks	range: 6 months-24 months
**Statistic:**	odds ratio	odds ratio	standardized mean difference
**Citations no:**	340	122	38

For peer review only
